# Population Genomics of a Rare and a Common Wood–Inhabiting Fungal Species Across Europe

**DOI:** 10.1111/mec.70260

**Published:** 2026-02-06

**Authors:** Franz‐Sebastian Krah, Mathias Scharmann, Alfons R. Weig, Jaqueline Hess, Harald Kellner, Antonis Athanasiadis, Enrico Büttner, Daniel Dvořák, Jan Holec, Reda Iršėnaitė, Kaisa Junninen, Irmgard Krisai‐Greilhuber, Vladimír Kunca, Sundy Maurice, Johannes Meier, Armin Mešić, Otto Miettinen, Kadri Runnel, Pablo Schäfer, Zdenko Tkalčec, Václav Pouska, Hermann Voglmayr, Max Zibold, Claus Bässler

**Affiliations:** ^1^ Fungal Ecology and BayCEER University of Bayreuth Bayreuth Germany; ^2^ Global Change Research Institute of the Czech Academy of Sciences Brno Czech Republic; ^3^ Plant Systematics and BayCEER University of Bayreuth Bayreuth Germany; ^4^ Genomics and Bioinformatics and BayCEER, Bayreuth Center of Ecology and Environmental Research University of Bayreuth Bayreuth Germany; ^5^ Department of Soil Ecology Helmholtz Centre for Environmental Research Halle Germany; ^6^ Department of Bio‐ and Environmental Sciences, International Institute Zittau Technische Universität Dresden Zittau Germany; ^7^ Thessaloniki Greece; ^8^ Department of Botany and Zoology Masaryk University Brno Czech Republic; ^9^ National Museum Prague Nové Město Czech Republic; ^10^ Laboratory of Mycology Nature Research Centre Vilnius Lithuania; ^11^ Metsähallitus, Parks & Wildlife Finland Vantaa Finland; ^12^ Department of Botany and Biodiversity Research University of Vienna Wien Austria; ^13^ Department of Applied Ecology, Faculty of Ecology and Environmental Sciences Technical University in Zvolen Zvolen Slovakia; ^14^ Section for Genetics and Evolutionary Biology (Evogene), Department of Biosciences University of Oslo Oslo Norway; ^15^ Rüstorf Austria; ^16^ Division for Marine and Environmental Research, Laboratory for Biological Diversity Ruđer Bošković Institute Zagreb Croatia; ^17^ University of Helsinki, Finnish Museum of Natural History Helsinki Finland; ^18^ Institute of Ecology and Earth Sciences University of Tartu Tartu Estonia; ^19^ Mannheim Germany; ^20^ Czech University of Life Sciences Prague Prague Czech Republic; ^21^ Naturkundemuseum Karlsruhe Karlsruhe Germany; ^22^ Bavarian Forest National Park Grafenau Germany

**Keywords:** dead‐wood saprotrophs, dispersal, fungal conservation, mycoparasite, population genomics, red‐list

## Abstract

Many species have become threatened during the Anthropocene, requiring conservation strategies based on biological evidence. Wood‐inhabiting fungi face multiple threats due to a complex interplay of a short lifespan, removal of dead wood as a resource and climate change. Furthermore, rare fruiting events might restrict dispersal via spores, leading to a significant population genetic structure. Yet, little is known about the genetic structure of both rare and common wood‐inhabiting fungal species across Europe. Here, we investigate the rare polypore fungus *Antrodiella citrinella,* which co‐occurs with the common wood‐decay fungus *Fomitopsis pinicola*. We analysed a total of 149 individuals of both species across 13 countries, sequenced their genomes and analysed single‐nucleotide polymorphisms. Based on a broad set of analyses, we found a very weak population structure in *A. citrinella,* suggesting historically wide dispersal and effective gene flow across Europe. In contrast, we found support for two moderately differentiated populations following a southwest‐northeast separation in *F. pinicola,* possibly due to dispersal limitation through its relatively larger spores, a more intense forest use history in southern Europe and a post‐glacial history of co‐immigration with the main host tree species, Norway spruce. While the weak to moderate genetic structure of wood‐inhabiting fungi suggests historically sufficient habitat connectivity, conservation measures should consider strategies providing deadwood as an important habitat to restore and maintain connectivity throughout Europe.

## 1 Introduction

Over centuries, European forest ecosystems have been heavily transformed by humans to optimise forest products like timber (Brunet et al. 2010; Grove 2002; Kaplan et al. 2009). Today, forest ecosystems are strongly fragmented and structurally modified (Kaplan et al. 2009). In particular, the alteration in tree species composition and the reduction in deadwood have caused a tremendous decrease in forest biodiversity (Grove 2002; Paillet et al. 2010). We require evidence for efficient conservation concepts to prevent forest biodiversity from further loss and to guide forest management. One crucial element is to acquire knowledge about the species' population structure to guide conservation efforts to determine relevant spatial scales. Our understanding of the conservation status of many rare and endangered species is still rudimentary, particularly for the fungal kingdom (Heilmann‐Clausen et al. 2015). Here, we assess the population genomic structure of the red‐listed *Antrodiella citrinella* Niemelä & Ryvarden (= *Flaviporus citrinellus* (Niemelä &s Ryvarden) Ginns) and the associated common species *Fomitopsis pinicola* (Sw.) P. Karst. across Europe.

**FIGURE 1:**
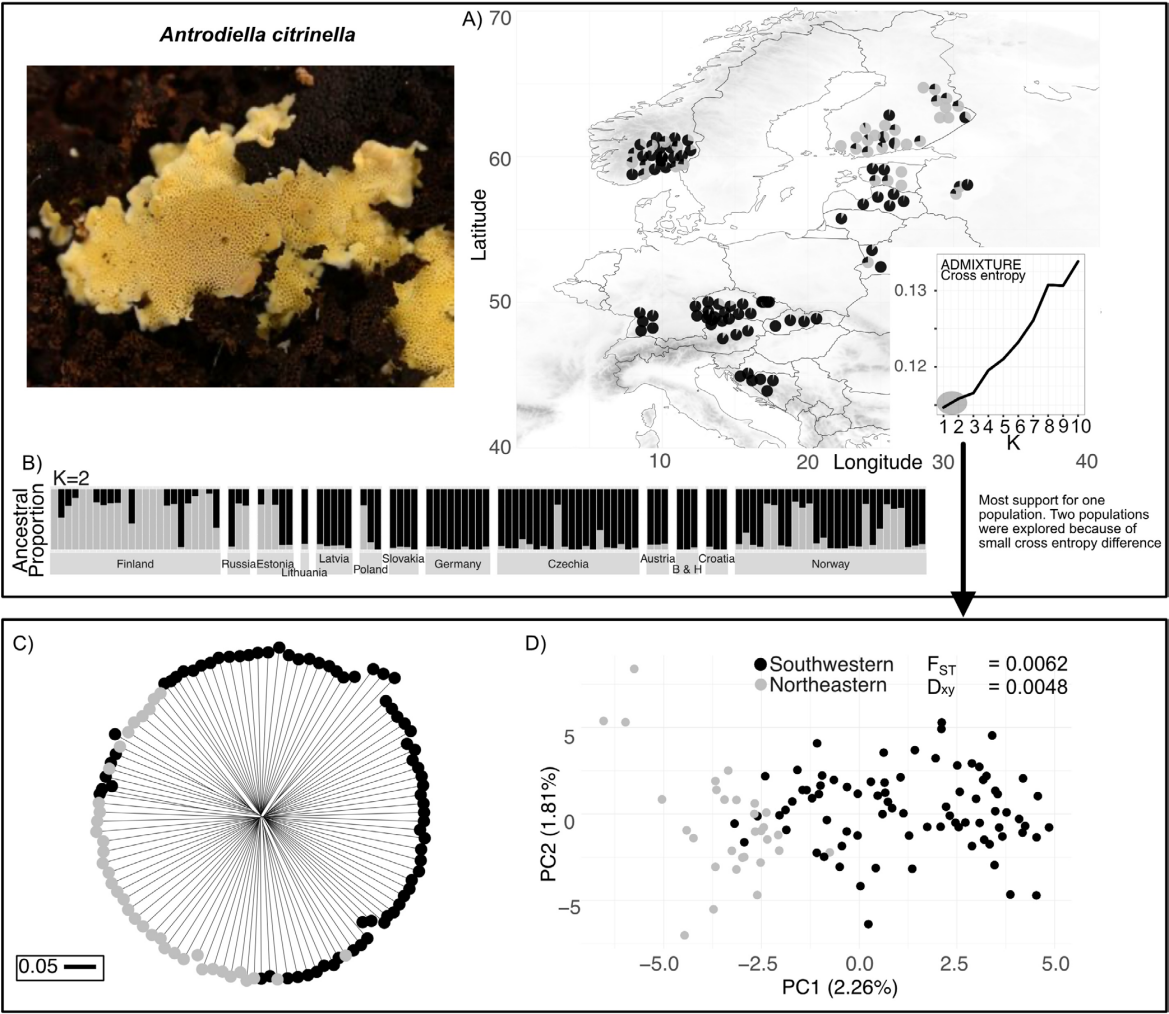
(A) Maps with sample locations across Europe for *Antrodiella citrinella* (Photographs by Peter Karasch) with analysis of the population structure (Pie charts on the map; jittered for visibility). The maps display elevation to help visualise potential barriers. The inset shows the cross‐entropy evaluation over a range of ancestral populations (K) by ADMIXTURE. We used black and grey colours to indicate weak support for putative southwestern and northeastern populations. (B) The barplot shows the individual ancestry proportions for two populations (*K* = 2) as estimated by ADMIXTURE. Results for higher K are shown in Figure S1. (C) Neighbour‐joining tree with the putative southwestern and northeastern ADMIXTURE clusters in black and grey. The scale shows the branch lengths of 0.05 arbitrary length. (D) Principal component analyses showing the putative southwestern and northeastern ADMIXTURE clusters.

*Antrodiella citrinella* is a polypore (Steccherinaceae) with a lemon to bright yellow pore surface (hymenium) (Figure 1). It is mainly distributed throughout Europe and occurs in natural forests dominated by or intermixed with Norway spruce ( *Picea abies* ) (Ryvarden and Melo 2014). *Antrodiella citrinella* is included in Red Lists in most countries, as well as classified as ‘endangered’ in the global IUCN Red List (Krisai‐Greilhuber 2019). A recent compilation of occurrence data showed that *A. citrinella* prefers deadwood‐rich natural and near‐natural forest stands, i.e., old‐growth forests, which are scarce throughout Europe (Holec et al. 2018). A threshold analysis revealed that *A. citrinella* requires more than 134 m^3^ ha^−1^ of deadwood in the temperate zone, similar to primeval forests (Bässler and Müller 2010). We lack information about minimum dead wood requirements in the boreal zone, where thresholds are likely lower. In the case of dead wood accumulation after disturbance and retention of dead wood, *A. citrinella* could even be recovered in former commercial forests (Bässler and Müller 2010). It is unclear if the resource alone is sufficient for the establishment or whether the currently regionally restricted occurrences are also limited by dispersal capability. Other rare polypore species were suggested to be dispersal‐limited even at small spatial scales (Moor et al. 2021; Norros et al. 2012).

The population genetic structure of a species can be influenced by the availability and spatial distribution of the required habitat, biotic interactions (e.g., antagonism, synergism), abiotic conditions (e.g., climate constraints) and dispersal limitation (Frankham 2002). First, due to forest fragmentation and forest use intensity, habitats for *A. citrinella* are scarce and patchily distributed throughout Europe (Holec et al. 2018; Sabatini et al. 2021). Second, *A. citrinella* obligatorily co‐occurs with another polypore fungus, namely *Fomitopsis pinicola*, the red‐belted bracket fungus. Fruit bodies of *A. citrinella* are found most exclusively in the presence of and on dead fruit bodies of *F. pinicola* (Wieners et al. 2016), while *F. pinicola* is widespread regardless of *A. citrinella* . Those field observations suggest mycoparasitism of *A. citrinella* on *F. pinicola* . Laboratory experiments of both species in culture showed antagonistic interactions, resulting in the death of the mycelium of *F. pinicola* , providing support for a mycoparasitic lifestyle (Wieners et al. 2023). Since *A. citrinella* mycelium could also grow independently of *F. pinicola* in culture (Wieners et al. 2023), we will use the term ‘hosts’ for both dead wood colonised by *F. pinicola* and fruit bodies on which *A. citrinella* occurs. If the primary host (spruce) of *F. pinicola* is phylogeographically structured, the fungus might also show a non‐random geographic population structure (Yguel et al. 2011). Norway spruce has been demonstrated to exhibit a southwest–northeast gradient in population structure based on mtDNA haplotypes (Thompson et al. 2005; Tsuda et al. 2016). Hence, if the geographic pattern of the host (spruce) influences the genetic population structure of *F. pinicola* , it might likewise affect the genetic structure of *A. citrinella* . We would thus expect a southwest‐northeast population structure in both fungal species due to their host relationships. Third, expectations about the dispersal capability of *A. citrinella* are not straightforward. The spore size of most fungi ranges from 2 to 50 μm in length (Patel et al. 2018), orders of magnitude smaller compared to, e.g., plant seeds (Aguilar‐Trigueros et al. 2023). Long‐distance dispersal (LDD) has been suggested (Golan and Pringle 2017; Hallenberg and Kuffer 2001; Moncalvo and Buchanan 2008), but it is unclear whether spores survive LDD and can successfully germinate and find a compatible mate after reaching a suitable habitat (Golan and Pringle 2017; Norros et al. 2015). Within an experimental setting, a rare wood‐decay fungus was shown to be dispersal‐limited on small geographical scales (Norros et al. 2012), while a review suggests that dispersal becomes limited only above the landscape scale (Komonen and Müller 2018). Parasites have often been shown to have more substantial population structuring than their hosts, especially when they have small effective population sizes, limited dispersal or narrow host ranges (Brandt et al. 2007; Delmotte et al. 1999), although contrasting results were found (Mazé‐Guilmo et al. 2016). As *A. citrinella* is considered a mycoparasite and is predominantly associated with *F. pinicola* , we hypothesise that it will show greater population differentiation than its host.

We generated a draft genome assembly for *A. citrinella* and sampled 110 and 39 individuals of *A. citrinella and F. pinicola*, respectively, throughout their distribution range across Europe. We conducted a genome‐wide single nucleotide polymorphisms (SNP) analysis of *A. citrinella* and *F. pinicola* genetic variation and placed our results in the context of genetic monitoring and conservation. We ask whether *A. citrinella* and *F. pinicola* show distinct population structures throughout Europe and whether there are signs of inbreeding or small population sizes in *A. citrinella* .

## 2 Material and Methods

### 2.1 Populations Sampled

**FIGURE 2:**
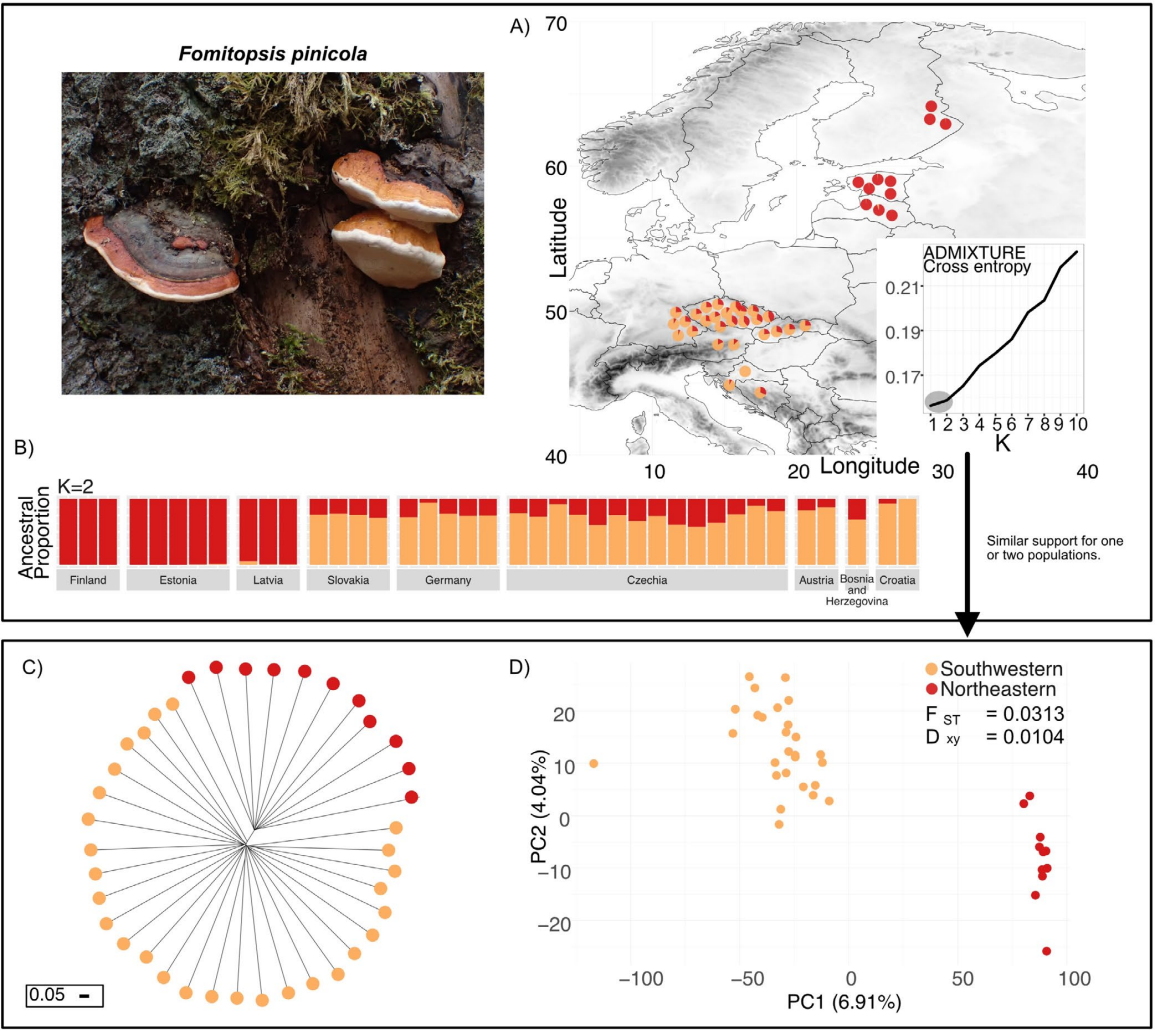
(A) Maps with sample locations across Europe for *Fomitopsis pinicola* (Photographs by Max Zibold) with analysis of the population structure (Pie charts on the map; jittered for visibility). The maps display elevation to help visualise potential barriers. The inset shows the cross‐entropy evaluation over a range of ancestral populations (K) by ADMIXTURE. We used red and orange colours to indicate moderate support for southwestern and northeastern populations. (B) The barplot shows the individual ancestry proportions for two populations (*K* = 2) as estimated by ADMIXTURE. Results for higher K are shown in Figure S2. (C) Neighbour‐joining tree with the southwestern and northeastern ADMIXTURE clusters in black and grey. The scale shows the branch lengths of 0.05 arbitrary length. (D) Principal component analyses showing the putative southwestern and northeastern ADMIXTURE clusters.

A total of 125 *A. citrinella* fruit bodies were collected from 13 different countries across Europe (Table S1, Figure 1; refer to 2.4. for the filtering of some samples). Since *A. citrinella* is a rare species and there was no prior information about population subdivisions, we used an opportunistic sampling strategy to maximise geographic coverage. At most, five fruit bodies were collected within a 10‐ha area. Fruit bodies mainly derive from the years 2017 and 2018, but also include older collections and material from the Helsinki herbarium collections (Table S1). Where available, fruit bodies of *F. pinicola* were collected at the same sites, yielding matched collections of 39 samples from 10 countries (Table S1, Figure 2). Necessary permits are available.

### 2.2 DNA Extraction and Sequencing of Samples

Small 5 × 5 mm pieces of dried fruit bodies were added to 2 mL Eppendorf tubes with tungsten beads and homogenised in a Tissue Lyzer bead mill. DNA was extracted using a cetyltrimethylammonium bromide (CTAB) extraction protocol and phenol‐chloroform purification as described in (Sønstebø et al. 2022). The extracts were cleaned up to remove PCR inhibitors using the Qiagen DNeasy PowerClean Pro cleanup kit (Qiagen, Hilden) according to the manufacturer's instructions. DNA concentrations were measured using the Qubit dsDNA BR Assay kit (Life Technologies, Carlsbad, CA, USA). Sequencing libraries were prepared using a Tn5 transposase tagmentation protocol (Picelli et al. 2014) at the Vienna Biocenter Core Facilities and sequenced in paired‐end mode for 300 cycles on an Illumina NovaSeq 6000 instrument with SP flow cells. Sequencing was performed on the Illumina NovaSeq 6000 platform, which uses patterned flow cells and two‐colour chemistry to provide high‐throughput, high‐quality short‐read data. This platform is widely used for large‐scale genomic studies due to its scalability and accuracy (Kim et al. 2021; Modi et al. 2021). Raw sequencing data are available at NCBI SRA under BioProject‐ID PRJNA1222407.

### 2.3 Sequencing and Annotation of the Reference Genome

*Antrodiella citrinella* DSM 108506 (ribosomal cistron accession no. MK503154) was collected from a rotting Norway Spruce ( *Picea abies* ) log in the Black Forest National Park (Wilder See, coordinates = 48.57258 N 8.2405 E) in November 2015 by Max Zibold (Wieners) and isolated by transferring fruit body pieces on an agar plate (cultures DSM 108506 and SBUG‐M 1723. See (Scholler and Popa 2021, 21; Wieners et al. 2016) and dried specimens in Herbarium KR, accession no. KR‐M‐0037905). The fungus was cultured in a liquid malt medium for biomass production. Genomic DNA was extracted using a standard CTAB‐based protocol. Genome sequencing was performed using the Ion Torrent PGM platform (Ion PGM Sequencing 200 kit version 2, 318v2 chip; 200 bp‐fragment library Ion Xpress Plus fragment library kit, Thermofisher, Darmstadt). The resulting reads were quality filtered (trim 3′ end; error probability limit, 0.05) and length filtered (180 to 381 bp were included) using Geneious R11. A total of 5.19 million reads of an average of 222 bp were de novo assembled using MIRA 4.0 (Chevreux et al. 1999) and in a second step with Geneious R11 (Kearse et al. 2012), to join contigs and to filter for duplicate contigs. The assembly consists of 1228 contigs with a total length of 33 Mbp (maximum contig size of 546,823 bp). The assembly was verified using QUAST v4.5 (Gurevich et al. 2013) and has a N50 of 66.9 kbp and a G + C content of 51.5%. The completeness of the assembly was assessed using BUSCO v3 (predictor: *Coprinus cinereus*, fungi dataset: Basidiomycota_odb9) and had a completeness of 98.7% (Simão et al. 2015). Gene prediction was performed using AUGUSTUS v3.2.2 (predictor *Coprinus cinereus*) (Stanke et al. 2004) and resulted in 9805 protein‐coding genes. Genes were annotated with Blast2GO v5.2.2 (BioBam, Valencia, Spain) and dbCAN3 (HMMdb v13, E‐Value < 1e‐15, coverage > 0.35) (Zheng et al. 2023). Altogether, 316 carbohydrate‐related enzymes and modules (among 27 carbohydrate‐binding modules of family 1, binding to cellulose) were identified.

### 2.4 Variant Calling and Filtering

Raw sequences from the population genomic samples were processed using Trimmomatic v0.38 (Bolger et al. 2014) to remove adapter remnants and poor‐quality bases, using ‘MAXINFO’ adaptive trimming with target length 40 and strictness of 0.2. Reads shorter than 50 bp were discarded. The read alignment was executed via the OmicsBox Version 3.3.2. Trimmed reads were aligned to the *A. citrinella* reference genome (NCBI‐GenBank GCA_004802725.1, assembly ASM480272v1, generated in this study) or the *F. pinicola* reference genome (NCBI‐GenBank GCA_001931775.1, assembly ASM193177v1, (Kancherla et al. 2017)), respectively using BWA_MEM v0.7.17 (Li and Durbin 2009). The size of the genome of *F. pinicola* is 46 Mbp. Note that within the reference genome of *A. citrinella* , seven positions coded as ‘S’ were recoded as ‘N’. We removed 12 samples of *Antrodiella citrinella* from the analysis, which had a coverage below 1× (Supporting Information S1), one sample because it was falsely labelled in the laboratory (DE_DE7Ac), and two from far eastern Russia and the USA, where species identification was unclear. We removed no sample for *F. pinicola* (Supporting Information S2). Thus, we finally analysed 110 of 125 samples for *A. citrinella* and 39 for *F. pinicola* .

We developed a custom pipeline to call and filter genetic variants from whole‐genome sequencing data (Supporting Information S3). In brief, we used BCFtools (Danecek et al. 2021) to call both bi‐allelic variants and invariant (monomorphic) sites, then filtered with the aid of VCFtools (Danecek et al. 2011) and custom scripts. Variants were filtered for a minimum quality score of 20, and those with an allele balance < 0.25 or > 0.75 (custom Python script, available at https://github.com/mscharmann/tools), as well as those appearing heterozygous in all samples, were removed. Genotypes at all sites (variant and invariant) were removed if the read depth (coverage) was lower than three and if the read depth exceeded the genome‐wide mean plus three standard deviations. This upper threshold was calculated specifically for each individual sample (custom Python script available as before). These criteria are effectively removing artefactual variants that appear due to the mapping of repetitive sequences onto the same position in the reference genome. Finally, we retained sites with a maximum of 10% missing data. For a data overview, see Table S1 and Supporting Information S4.

### 2.5 Population Structure Analyses

Population structure among the samples was investigated using four complementary methods. (i) To apply ADMIXTURE analysis, we used the SNP data matrix and loaded it into R using the vcfR R package. To run ADMIXTURE, we used the sNMF algorithm via the *snmf* function from the LEA R package (Frichot and François 2015). We extracted the genotypes using the function extract.gt and subjected them to the *snmf* function. We recoded missing data as ‘9’, which are not used by the program to estimate population structure. The optimal number of ancestral populations (denoted as ‘K’) was determined using the entropy criterion to estimate the optimal number of populations. This method identifies the K‐value with the least cross‐validation error (Alexander and Lange 2011; Frichot et al. 2014). We used the ADMIXTURE results to inform the demographic modelling of the two species (see below). (ii) Neighbour‐joining (NJ) trees were estimated with rapidNJ (Simonsen et al. 2008) from all sites (i.e., including invariants and retaining heterozygote genotypes as IUPAC ambiguity code). (iii) We conducted principal component analysis (PCA) using *smart_pca* from the smartsnp R package (Herrando‐Pérez et al. 2021), considering only the variant sites with centring. Note that our filtering allowed for missing data, which are not allowed in PCAs and are mean imputed within smart_pca. Therefore, we filtered to a maximum of 1% missing data for the PCA. We report the results for the 1% missing data in the main body and show the results with 10% missing data in Figure S3. The analyses with 1% or 10% showed a consistent pattern of population structuring. (iv) For the isolation by distance analysis, we calculated an identity by state distance matrix (IBS) between all pairs of individuals using PLINK 1.9 (Purcell et al. 2007) with the ‘‐‐distance 1‐ibs’ command. We tested the relation between IBS and the haversine geographic distance using the Multiple Regression on distance matrix (MRM) framework implemented in the ecodist R package (Goslee and Urban 2007). We report the R^2^ and *p*‐value within the scatterplot showing the relationship. Note finally that we also used a dataset for PCA and ADMIXTURE that was pruned for strong linkage. For linkage pruning, we used PLINK with default settings: a sliding window of 50 SNPs (shift of 5 SNPs) in which pairwise linkage is calculated, removing SNPs with an R^2^ greater than 0.2. Results were consistent across settings (data not shown).

### 2.6 Demographic Modelling

We studied the demographic history of both species by comparing three scenarios with Fastsimcoal2 v2.8 (Excoffier et al. 2021). We were especially interested in additional evidence for population subdivision and also in the relative population size changes, divergence times and potential migration rates. Population subdivision was guided based on our expectation about a potential southwest–northeast structuring (see Section 1) and the geographic distribution of the ADMIXTURE ancestry proportions for a K‐value of 2 across multiple runs (see above, and insets in Figures 1A and 2A). From these criteria, we defined the northeastern population containing samples from Finland for *A. citrinella* (Figure 1A). For *F. pinicola* , we defined the northeastern population to contain samples from Finland, Estonia and Lithuania (Figure 1B). The other samples were grouped as the southwestern population. Note that samples from Norway, even though located in the north, were grouped into the latter for *A. citrinella* , based on our statistical and visualisation exercise. The grouping of the three *A. citrinella* samples from Russia and Estonia was not straightforward. Since they showed strongly intermixed samples, similar to Norway, we placed them in the southwest population based on the ADMIXTURE results (see Figure 1A,B). Assigning them to the northeast population instead did not change the inferences of the demographic model. For demographic modelling, we created the input joint site frequency spectrum (folded) using easySFS (Gutenkunst et al. 2009) with the two populations (southwest and northeast), including invariant sites. We then evaluated three demographic scenarios. The first scenario assumed a single population across Europe with a constant size. The second scenario modelled a divergence into two populations at some point in the past, with no contact after the split (isolation), and constant sizes for both populations. The third scenario replicated the second but added migration rates between the split populations. To ensure comparability via likelihoods and Akaike's information criterion (AIC) between the single‐population model and those models with two populations, we used the same joint SFS input file for all three models. Since the joint SFS must contain two populations, we framed the single‐population scenario as a special case of the two‐population scenario by fixing the divergence time parameter to zero, i.e., coalescence between the (pseudo‐) populations was allowed to occur immediately, just like in a single population. We then estimated the effective population size Ne for the ancestral and recent populations with priors 100–10,000 (Sønstebø et al. 2022). Note that the upper bound is not a hard constraint in Fastsimcoal2, and the estimates obtained were entirely robust to the choice of a higher upper boundary (100,000). For the second scenario, we allowed the divergence time parameter to be estimated with a prior of 50–300, following a similar setting as another study using a polypored species (Sønstebø et al. 2022). For the third scenario, we further allowed migration (gene flow) with (soft) prior bounds of 0.0001–0.1. The spontaneous mutation rates for basidiomycetes are generally poorly known. One estimate is 1 × 10^−8^ per site per generation (Baranova et al. 2015; Hiltunen et al. 2019; Thorén et al. 2025), while a study on a polypore taxon used 1 × 10^−7^ per site per generation (Sønstebø et al. 2022). To explore the effect of this uncertainty, we repeated all analyses for these two different mutation rates. For each scenario, we ran 1000,000 coalescent simulations and 60 Expectation‐Conditional Maximisation (ECM) cycles for likelihood estimation. We calculated the Akaike Information Criterion (AIC) as 2*k* − 2(MaxEstLhood/log_10_(e)), where *k* is the number of parameters. We further calculated the relative AIC difference from the best model as ΔAIC (the best scenario shows ΔAIC = 0). We conducted three independent simulation runs and found consistent ΔAIC values (data not shown). Note that the sample size was high enough in both species to accurately estimate divergence times (McLaughlin and Winker 2020).

### 2.7 Linkage Disequilibrium Decay

Linkage disequilibrium (LD) was estimated between bi‐allelic variants with the geno‐r2 option in VCFtools (Danecek et al. 2011), applying a maximum distance of 10 kb and a window size of 5 kb. A window size of 5 kb balances resolution and computational efficiency, allowing for robust estimation of local LD patterns while limiting noise from sparsely distributed SNPs.

### 2.8 Genetic Diversity and Divergence

Within each species, we calculated genome‐wide summary statistics. We did calculations across all samples (Europe) and for the southwestern and northeastern populations. We used bi‐allelic variants and invariant sites to calculate absolute nucleotide diversity indices (*π* and *θ*) and Tajima's *D* with pixy 1.2.7. (Korunes and Samuk 2021) and a window size of 100,000 bp. We further used pixy to calculate *F*_*st*_ and *D*_*xy*_ as measures of population divergence between the putative southwest and northeast populations. We used PLINK 1.9 (Purcell et al. 2007) to calculate the individual heterozygosity from the variant sites only and averaged this measure per sample using the ‘‐‐het’ flag. Finally, to test for signs of inbreeding, we calculated the inbreeding coefficient *F*_*IS*_, as well as runs of homozygosity (ROH) for each sample to subsequently calculate the inbreeding coefficient *F*_*ROH*_ (Gibson et al. 2006). *F*_*IS*_ was calculated using PLINK with the ‘‐‐het’ flag as above for the heterozygosity. Runs of homozygosity (ROH) were also calculated using PLINK with the ‘‐‐homozyg’ flag with only runs of homozygosity containing at least 100 SNPs, and of total length ≥ 100 kilobases and a density requirement of at least one SNP per 50 kilobases. For each sample, we quantified the total ROH length and subsequently the inbreeding coefficient *F*_*ROH*_ by dividing the total ROH length by the genome size (McQuillan et al. 2008). Analyses of ROH have recently been proposed and promoted for fungal population genomics analysis (Brejon Lamartinière et al. 2024).

### 2.9 Environment Associations

Associations between genotypes and environmental variables were examined using a multivariate approach that assessed SNP variation in relation to climatic factors through redundancy discriminant analysis (RDA). Environmental data included the 19 standard bioclimatic variables from WorldClim2 (http://worldclim.org/version2) at a spatial resolution of 0.5 arc‐minutes. To minimise multicollinearity among climatic predictors in the RDA, variables with a Spearman correlation coefficient (|ρ|) greater than 0.7 (Dormann et al. 2012) were removed using the findCorrelation function from the *caret* R package. This filtering resulted in the retention of six and seven climatic variables for *Antrodiella citrinella* and *Fomitopsis pinicola*, respectively. The RDA was constrained by these environmental variables to maximise the proportion of genetic variation explained. All RDA analyses were carried out using the *vegan* R package. This approach enables the detection of outlier loci that show a significantly stronger association with the environmental variables shaping the RDA axes compared to the average locus. These outliers are more likely to be targets of selection, whereas the remaining loci are primarily shaped by neutral processes such as genetic drift and gene flow. The method has been demonstrated to produce a low rate of false positives in detecting selection, particularly in populations with weak genetic structure (Forester et al. 2018). We identified outlier loci based on their loadings on the first and second RDA axes. Loci with loadings greater than three standard deviations from the mean were classified as significantly associated with environmental gradients (Forester et al. 2018). Further, we performed a permutation test with 100 permutations using the anova.cca function in R with ‘by = “margin”’ argument to retrieve significance tests for each variable on the sites separately. We used the marginal tests because we were not interested in the relative effects, which would require a higher number of degrees of freedom, but simply in potentially significant variables. We added a significance label to the plot.

## 3 Results

For the rare species *Antrodiella citrinella*, we obtained 27.4 million sites total among which were 694,525 bi‐allelic SNPs (ca. 2.5%) in the European population as a whole (*N* = 110, Table 1). The common species *Fomitopsis pinicola* yielded a total of 30.3 million sites and was more diverse despite the much lower sample size (*N* = 39), with 1.9 million bi‐allelic SNPs over our whole sampling (ca. 6.2%; Table 1).

**TABLE 1:** Summary of genetic diversity statistics for two fungal species (*Antrodiella citrinella* and *Fomitopsis pinicola*) across European populations.

Species	*Antrodiella citrinella*			*Fomitopsis pinicola*		
Population	Europe	Northeast	Southwest	Europe	Northeast	Southwest
*Total sites*	27,403,897			30,335,713		
*SNPs*	694,525			1,907,257		
*π*	0.0049	0.0047	0.0049	0.0102	0.0103	0.0100
SE	0.0001	0.0001	0.0001	0.0002	0.0002	0.0002
*θ*	0.0058	0.0050	0.0057	0.0109	0.0107	0.0103
SE	0.0001	0.0001	0.0001	0.0002	0.0002	0.0002
Tajima's *D*	−0.54	−0.27	−0.49	−0.32	−0.28	−0.25
SE	0.02	0.02	0.02	0.02	0.02	0.02
*Het*	0.13	0.10	0.13	0.17	0.18	0.17
SE	0.00	0.01	0.01	< 0.01	< 0.01	< 0.01
*F*_*IS*_	0.043			0.013		
SE	0.02			0.02		
*F*_*ROH*_	0.0003			0.00004		
SE	< 0.0001			< 0.0001		
*F*_*ST*_		0.0062			0.0313	
SE		0.0008			0.0012	
*D*_*xy*_		0.0048			0.0104	
SE		0.0001			0.0002	

*Note:* The total number of genomic sites analysed (with and without invariant sites, i.e., SNPs), nucleotide diversity (*π*), Watterson's theta (*θ*), Tajima's *D*, the average observed heterozygosity per individual (Het), two inbreeding coefficients *F*_*IS*_ and *F*_*ROH*_, and two indices for population differentiation, the fixation index *F*_*ST*_ and absolute nucleotide divergence *D*_*xy*_. For each statistic, we also report the standard error.

### 3.1 Population Structure, Diversity and Demographic Model of *Antrodiella citrinella*

**FIGURE 3:**
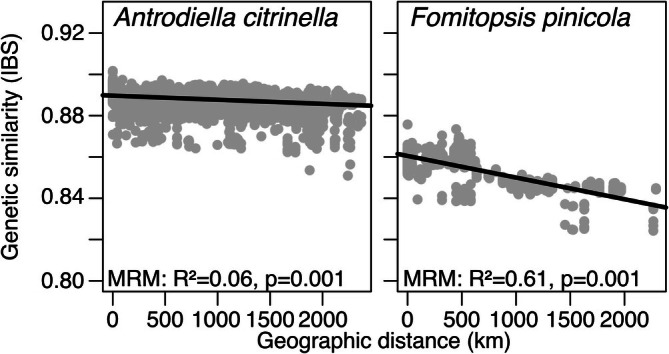
Isolation by distance pattern in the studied populations. Pairwise identity‐by‐state (IBS) values against geographic distances between individuals. The significance of the relationships was assessed using Multiple Regression on distance Matrices (MRM), which supported the presence of isolation by distance for both species.

An analysis of the population structure of *A. citrinella* with ADMIXTURE showed that the lowest cross‐entropy was obtained for *K* = 1 (Figure 1A). However, cross‐entropy showed weak differences between *K* = 1 and *K* = 2 (Figure 1A, inset). For *K* = 2, we found a structuring that roughly clustered into a northeast population and the rest of Europe. The putative northeast population includes mainly Finland (Figure 1A,B). Norwegian samples clustered more within the southwest population but showed ancestry from both clusters. Even in Finland, we found samples with some ancestry from the southwestern cluster (Figure 1A,B). Further, the Estonian and Russian samples also showed clear ancestry from both clusters. Results for *K* = 3 and *K* = 4 are shown in Figure S1. The NJ tree showed a similar clustering consistent with the southwest‐northeast interpretation for *K* = 2. Yet the tree shows extremely short internal branches, while the terminal branches of the tree are comparatively much longer, indicating that the vast majority of genetic diversity is between individual samples regardless of their sampling location. Consequently, genetic diversity is likely not between geographic populations, indicating weak population structure (Figure 1C). The PCA analyses showed two clusters for *A. citrinella* on the first PCA axis, but with overlap between the putative populations. This result was robust to the choice of missing data thresholds (1% or 10%, Figures 1D and S3). We also found a significant but weak negative slope in an isolation by distance analysis (Figure 3).

Nucleotide diversity (*π*) for *A. citrinella* in Europe as a whole was estimated at 0.0049 (SE = 0.0001), with a negative Tajima's *D* of −0.54, meaning a mild excess of rare alleles (Table 1). Waterson's *theta* (*θ*) was estimated at 0.006, and average heterozygosity was 0.13. In the southwest and northeast populations of *A. citrinella* , *π* values were slightly lower (0.0047 and 0.0049, respectively), with more negative Tajima's *D* values in the Southwest (−0.49) compared with the Northeast (−0.27). Heterozygosity was lower in the Northeast (0.10) compared to the Southwest (0.13). Inbreeding was not apparent, given the close to zero estimates of both *F*_*IS*_ and *F*_*ROH*_ (Table 2). Furthermore, populations showed very weak to zero differentiation (*F*_*ST*_) and absolute divergence (*D*_*xy*_), i.e., again no obvious population structure (Table 2).

**TABLE 2:** Demographic inference for the fungal species *Antrodiella citrinella* and *Fomitopsis pinicola*. Best model scenario based on ΔAIC is highlighted in bold.

Species	Scenario	Ne anc	Ne SW	Ne NE	Tdiv	Mig SW → NE	Mig NE → SW	MaxEst Lhood	AIC	ΔAIC
*Mutation rate: 10* ^*−7*^										
*Antrodiella citrinella*	Single population	20,318						−23,530	108,365	451
	Two pop no migration	17,108	140,709	112,337	329			−23,435	107,928	14
	**Two pop and migration**	**17,209**	**64,156**	**32,181**	**434**	**0.0096**	**0.0094**	**−23,431**	**107,914**	**0**
*Fomitopsis pinicola*	Single population	66,126						−56,505	260,223	1494
	**Two pop no migration**	**62,916**	**16,577**	**55,690**	**527**			**−56,181**	**258,729**	**0**
	Two pop and migration	62,476	79,513	83,788	182	0.0398	0.0725	−56,486	260,141	1412
*Mutation rate: 10* ^*−8*^										
*Antrodiella citrinella*	Single population	20,321						−23,528	108,365	469
	Two pop no migration	175,043	1,186,907	645,401	2921			−23,428	107,930	43
	**Two pop and migration**	**16,592**	**80,171**	**35,774**	**678**	**0.0002**	**0.0083**	**−23,424**	**107,888**	**0**
*Fomitopsis pinicola*	Single population	627,862						−56,484	260,125	2060
	Two pop no migration	651,113	40,432	40,229	935			−56,245	259,027	962
	**Two pop and migration**	**59,460**	**47,518**	**66,535**	**1948**	**0,0001**	**0,0001**	**−56,839**	**258,065**	**0**

*Note:* Point estimates generated with Fastsimcoal2 from three demographic models: (1) one population across Europe, (2) two populations with a split between southwestern (SW) and northeastern (NE) Europe with isolation and (3) two populations with a split between southwestern and northeastern Europe with migration. Ne anc is the effective population size in the ancestral population, N SW and N NE are the effective population sizes in the recent population in the Southwest and the Northeast; Tdiv is the divergence time (number of generations between populations); Mig SW → NE is the rate of migration of individuals from Southwest to Northeast and Mig NE → SW in the opposite direction.

Demographic modelling with Fastsimcoal for *A. citrinella* best supported a scenario of population divergence between southwest and northeast Europe with ongoing migration (Table 2) and was robust to the choice of the assumed mutation rate. This model infers that the populations split only relatively few generations ago (~400 and ~700 generations ago for the two mutation rates, respectively). Importantly, these split times are very shallow relative to the effective population size of the ancestral population, only ca. 0.025–0.04 generations/Ne (coalescent units), indicating that genetic drift could not have had any strong effect yet, and fully consistent with the near‐zero estimates of *F*_*ST*_ and *D*_*xy*_ above. The model with faster mutation rates found low but symmetric gene flow between regions, and the model with slower mutation rates found higher migration rates from southwest to northeast (Table 2). In both cases, the effective migration rates (ancestral Ne * m) were far greater than 1.0. Under classical population genetic theory, values Ne * m ≥ 1 are sufficient to counteract genetic drift and prevent strong population differentiation (Slatkin 1987; Whitlock and McCauley 1999). Because Ne * m is estimated over the duration of the inferred demographic history, this interpretation refers to cumulative gene flow over many generations rather than contemporary migration alone.

### 3.2 Population Structure, Diversity and Demographic Model of *Fomitopsis pinicola*

An analysis of the population structure of *F. pinicola* with ADMIXTURE showed that the lowest cross‐validation entropy was obtained for *K* = 1 (Figure 2A,B). However, similar to *A. citrinella* , cross‐validation entropy showed very weak differences between *K* = 1 and *K* = 2 (Figure 2A inset). For *K* = 2, we found a separated northeastern and southwestern population (Figure 2A,B). For *K* = 3 and 4, see Figure S2. In contrast to *A. citrinella* , *F. pinicola* had a more pronounced population structure, which might be at least partly explained by the lower number of samples and spatial coverage. The NJ tree recapitulated the *K* = 2 ADMIXTURE clusters with a southwest and northeast population, and although the terminal branches were again dominating in this species, the internal branches were relatively longer than for *A. citrinella* , once more indicating a stronger population structure (Figure 2C). The same two clusters were again clearly separated without overlap along the first principal component (Figure 2B; Figure S3). We also found a significantly negative and steep slope of genetic distance with geographic distance in this species compared to *A. citrinella* (Figure 3).

Nucleotide diversity in *F. pinicola* was more than twofold higher than in *A. citrinella* , with *π* = 0.010 and *θ* = 0.011 (Table 1). Tajima's *D* was slightly negative (−0.32) but less negative than for *A. citrinella* . Heterozygosity in European populations was 0.17. Regional breakdowns of *F. pinicola* showed comparable diversity estimates. In the Northeast, *π* was 0.0103, Tajima's *D* was −0.28, and heterozygosity reached 0.18, whereas the Southwest exhibited slightly lower diversity (*π* = 0.010, Het = 0.17) and a Tajima's *D* of −0.25. Standard errors were consistently low across all estimates. The somewhat more pronounced, but still moderate population structure in *F. pinicola* was also evident in greater *F*_*ST*_ and *D*_*xy*_ between the southwestern and northeastern populations (Table 2). As in *A. citrinella* , both inbreeding coefficients (*F*_*IS*_, *F*_*ROH*_) were close to zero (Table 2), and linkage disequilibrium decayed rapidly for both species (Figure S4).

The best‐supported demographic scenario for the higher mutation rate assumption was that with two fully isolated populations in southwest and northeast Europe, with the split estimated at ca. 500 generations ago (Table 2). The best‐supported scenario for the lower mutation rate assumption was that in which the two populations continued to exchange migrants and had a split time estimate of ca. 2000 generations ago (Table 2). Here, the split times are around 0.008, respectively 0.032 generations/Ne (coalescent units, using the ancestral Ne) ago. Together with the decreasing size of the southwestern daughter population, as found with the lower mutation rate estimate, may have allowed for drift to take stronger effects than in *A. citrinella* . Although in *F. pinicola* the effective migration rates are also greater than 1.0 and thus have potentially ample power to homogenise the southern and northern populations on evolutionary time scales, they are lower than in *A. citrinella* , consistent with the more pronounced differentiation and divergence found above.

### 3.3 Environment Associations

**FIGURE 4:**
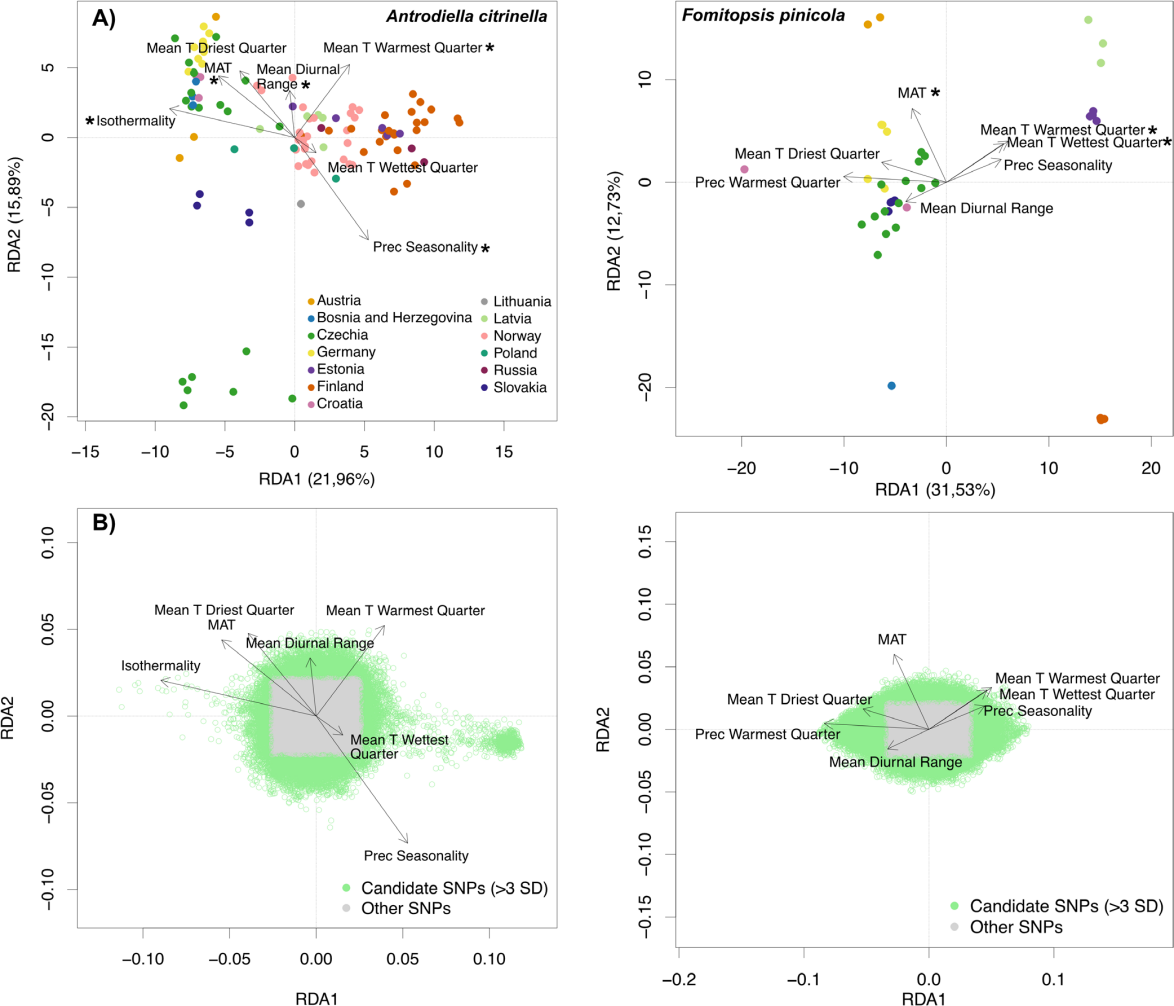
Constrained redundancy analyses of the SNP variation explained by environmental variables. (A) The two top plots show the sample results of the redundancy analysis (RDA). The ordination shows points that indicate the individuals of both species (coloured by country) inferred in the RDA analyses and projected along the first two axes. We selected WORLDCLIM variables with a correlation coefficient below 0.7 and assessed significance using a permutation test for each variable indicated by a star. (B) The two lower plots show the SNP results of the RDA. The ordination shows the projection of SNPs and explanatory environmental variables in the RDA. Significant SNPs were defined as having values greater than three standard deviations.

The RDA analysis revealed a stronger differentiation of the locations along the axes (Figure 4A) constrained by climatic variables than along the unconstrained PCA axes (Figure 1). Further, the first two RDA axes explained comparably high parts of the variation, with ca. 38% explained variability for *A. citrinella* and 44% for *F. pinicola* . For *A. citrinella* , especially isothermally, temperature for the driest quarter, mean annual temperature, mean temperature of the warmest quarter and precipitation seasonality explained variation along with the first RDA axis, which separated the samples along the latitudinal geographic axes, from Croatia via Latvia to Norway and Finland (Figure 4A). Isothermally also explained a majority of significant SNPs along with the first RDA axis for *A. citrinella* (Figure 4B). For *F. pinicola* , especially the temperature of the warmest and wettest quarter, and mean diurnal range explained variability along the first RDA axis, which corresponded also to the latitudinal geographic axes (Figure 4A). Especially, precipitation in the warmest quarter explained a majority of significant SNPs along with the first RDA axis (Figure 4B).

## 4 Discussion

Many wood‐inhabiting fungal species are rare or endangered (Nordén et al. 2020); however, the underlying mechanisms are poorly understood, prohibiting a mechanistic understanding and conservation strategies. Here, we used population genomic analyses based on SNP data and found weak structuring for the rare polypore *Antrodiella citrinella*. For the common fungal polypore *Fomitopsis pinicola*, we revealed moderate population structuring with a southwest–northeast division across Europe. Even though weak to moderate, conservation initiatives should take into consideration the continental‐scale structuring when informing conservation strategies.

### 4.1 Population Structure of the Rare Fungal Species *A. citrinella*

Results of the ADMIXTURE, neighbour joining tree and isolation by distance analysis indicated no to weak population structure. Although the PCA analysis discriminated slightly between the two populations, following roughly a southwest–northeast pattern, the clusters overlapped, and divergence estimates (*F*_*ST*_, *D*_*xy*_) were almost zero, indicating no significant structuring. The demographic modelling approach indicated that the best scenario involved two populations with rather recent divergence times and strong effective migration. Therefore, based on the different lines of evidence, we would argue for weak population structure across Europe for *A. citrinella* . Two rare wood‐inhabiting fungal species have been studied in this context. For *Fomitopsis rosea*, little spatial population structuring was found on a sub‐regional scale across Fennoscandia (Kauserud and Schumacher 2003). At the same spatial scale, no significant population structure was further found for *Phellopilus nigrolimitatus*, a rare and red‐listed wood decay fungus with spruce as primary host, based on early approaches with limited genetic information (Kauserud and Schumacher 2002). A more recent study on *Phellopilus nigrolimitatus* suggested two populations in Fennoscandia, one in Norway and one in Finland, but with rather weak structuring (Sønstebø et al. 2022). Similar to our analysis for *A*. *citrinella*, this study also found most support for a single population by ADMIXTURE analysis (Sønstebø et al. 2022). Although a limited number of studies are available, especially for rare fungal species, most findings indicate weak population structuring. One reason might be the insufficient geographic extent of sampling. We maximised the sampling extent across Europe, covering the temperate and boreal zones, and found no substantial population structuring. This indicates that the limited spatial extent of early studies did not limit the power to detect structuring, but instead that structuring is actually weak or absent. Further, even though *A. citrinella* is considered rare, we found no sign of inbreeding considering the *F*_*IS*_ and *F*_*ROH*_ values, suggesting efficient gene flow across the sampled area.

### 4.2 Population Structure of the Common Fungal Species *Fomitopsis pinicola*

We found a stronger pattern of two non‐overlapping clusters in the PCA analysis compared to *A. citrinella* . Further, the two clusters formed two obvious groupings in the neighbour‐joining tree and we observed a stronger negative slope in the isolation by distance analysis. The demographic modelling additionally suggests that there are either two populations with a very shallow split time and no migration or a somewhat deeper split time and effective migration rates that are strong enough to nearly homogenise the populations, depending on the mutation rate assumed (Figures 2 and 3). On the other hand, ADMIXTURE and direct estimates of population divergence using *F*_*ST*_ and *D*_*xy*_ suggest moderate structuring. The nucleotide diversity (*π*) in *F. pinicola* of ca. 1% is similar to that reported for other Agaricomycetes (Zhao and Xu 2023), although we note that methodologically comparable results (i.e., whole‐genome‐resequencing data and geographically broad sampling scheme) are extraordinarily rare for fungi. Therefore, based on the different lines of evidence, we would argue for a moderate population structure across Europe for *F. pinicola* . Existing genetic studies of common fungi found support for a significant population structure depending on the spatial scale: In an early study, no significant population structure of *F. pinicola* was found on a regional scale across northern Europe (Högberg et al. 1999). The fact that only northern Europe has been studied in Högberg et al. (1999) might explain the contrast to our findings. Low levels of population structuring were also found for a common ectomycorrhizal species, *Russula virescens*, on the regional scale across Northern China (Wang et al. 2015). However, in a larger‐scale study across China, three populations were strongly supported for the common wood‐inhabiting fungus *Lentinula edodes* (Zhang et al. 2022). Likewise, in a global‐scale study of *Trichaptum abietinum*, multiple populations were found, and two populations were found separating northern and southern Europe (Lu et al. 2024). Thus, on the continental to global scales, if multiple biomes are covered, the signals were stronger for more than one population. Our study was on a regional scale and covered two major European biomes, the boreal as well as the southern and northern temperate zone, and we found moderate signs of population structuring supporting this view.

### 4.3 Demographic History

Our findings align poorly with many plant species that show strong population structure across Europe, with population breaks between southern and central European populations, likely resulting from vicariance during ice ages (Ansell et al. 2010; Clauss and Mitchell‐Olds 2006; Pauwels et al. 2012). Likewise, for mammals, analyses have shown a strong correlation between genetic structure and geography in European populations (Hewitt 2011; Nelis et al. 2009). Our results across Europe suggest a moderate southwest and northeast population structure for *F. pinicola* and a weak structuring for *A. citrinella* . Even though population genomic data for fruit body‐forming fungi are still scarce, we carefully suggest that the strength of population structuring across Europe might generally be less intense in fungi than in plants and mammals. Interestingly, there are still differences among fungal species as outlined below.

According to the demographic model, we found divergence ca. 527–1948 generations ago for *F. pinicola* . Unfortunately, the generation time of *F. pinicola* is poorly known. A recent publication argued for 20–40 years for the wood‐decay fungus *Phellopilus nigrolimitatus* that colonises early and fruits at late stages of decay, which might take about 40 years (Sønstebø et al. 2022). In contrast, *F. pinicola* can already produce fruit bodies in the initial stages of decay (Krah et al. 2018) and thus, a lower boundary for generation times might be only 1–2 years between colonisation and fruiting. *Fomitopsis pinicola* can sporulate throughout many years from the same, further‐growing fruit body. We expect the lifespan to depend on the substrate amount, i.e., larger logs yield longer lifespans of wood decay fungi. Since dead wood size distribution usually is right‐skewed (Juutilainen et al. 2011), we expect most fruit bodies to sporulate 2–6 years and only rarely during 10 years or longer. Considering these estimates, divergence of the two populations would correspond to ca. 4000–12,000 years ago for *F. pinicola* . These estimates could correspond to the end of the last ice age, ca. 10,000 years ago and thus, the demographic history of *F. pinicola* might be similar to that of many European plants and animals, but with less strongly pronounced structuring as outlined above. The relatively weak population structure inferred here suggests that long‐term isolation and strong inbreeding are unlikely. These results imply that gene flow has likely been sufficient over evolutionary timescales to limit strong divergence and maintain genetic connectivity between populations. Another study proposed an ice‐age‐related population structuring and co‐migration with the main host *Picea abies* for the polypore *Phellopilus nigrolimitatus* (Sønstebø et al. 2022). However, while *P. nigrolimitatus* occurs mainly on *Picea abies* as primary host, *F. pinicola* occurs on a wide host range, occurring on dead wood of angio‐ and gymnosperm tree species but with a clear host preference towards *Picea abies* (Krieglsteiner 2000). Thus, one further explanation contributing to the observed population structure in *F. pinicola* could be that co‐migration is related to different host tree species. The northern population might have remained with the spruce refugia in northeast areas (Tollefsrud et al. 2008; Tzedakis et al. 2013), while the southwestern population survived with different potential host tree species. For example, the Dinaric Alps were a refugium for many tree species, such as beech ( *Fagus sylvatica* ), oak (*Quercus* spp.) species or silver fir ( *Abies alba* ) (Brus 2010; Gentili et al. 2015; Gömöry et al. 2020).

The overall moderate structuring of *F. pinicola* across Europe, as compared to plants and animals, might result from high dispersal capabilities of fungi due to small dispersal propagules. Thus, the population of *F. pinicola* could have mixed again after the ice age and during co‐migration back from the refugia, as also discussed in the study based on *Phellopilus nigrolimitatus* (Sønstebø et al. 2022). Nevertheless, the moderate structuring of *F. pinicola* in contrast to the weak structuring observed for *A. citrinella* suggests that dispersal limitation might play a role in preventing rapid admixture to a panmictic population. The spore size of *F. pinicola* is 6–8.5 × 3–4.5 μm, while the spore size of *A. citrinella* is roughly half, with 3–3.5 × 2–2.5 μm (Ryvarden and Melo 2014). Simulations showed that a difference of 3–5 μm can have significant effects on the travelling downward speed of spores (Norros et al. 2014), indicating that even small differences in size between spores might have biologically meaningful effects on their dispersal and thus gene flow. Therefore, the structuring we observe in *F. pinicola* might be the result of a combination of host‐migratory divergence during the last ice age, combined with a limited dispersal (compared to *A. citrinella* ), also limiting gene flow on the continental scale, while *A. citrinella* could maintain gene flow through dispersal due to substantially smaller spores. This is in contrast to our expectation that the parasitic lifestyle of *A. citrinella* would lead to a subordinate population structure following the structuring of its host *F. pincola*. The population structure of the higher trophic level (mycoparasite), thus, seems to be more determined by its dispersal ability than by host relations, while the lower trophic level (saprotroph) is more structured by both the host geographic distribution as well as dispersal ability. Nevertheless, further studies should examine the dispersal ability of both species to test our above‐developed hypothesis, and whether there is a general relationship between spore size (as a proxy for dispersal ability) and population structure (as a proxy for realised dispersal) in fungi. We further found that climatic variables correlate with the latitudinal structuring of the population of *F. pinicola*, and SNPs that were significantly associated with environmental variables were more strongly correlated with climate than for *A. citrinella* in the RDA analysis. This supports the view that climatic changes, such as the ice age, had a stronger effect on *F. pinicola* , but climate might show a correlation simply due to divergence along the latitudinal axis, which reflects major climatic gradients. Divergence times showed a wide range, and considering the uncertainty regarding the generation time, divergence times might be even higher or lower. We found generally higher ancestral population size in *F. pinicola* than in *A. citrinella* , which might result from the highly frequent and dominant fruit body production (e.g., there are ca. 130,000 records on GBIF for *F. pinicola* as compared with ca. 2500 for *A. citrinella* by the time of publication). Interestingly, we found a lower population size and lower nucleotide diversity in the southeastern population than in the northwestern population, while the northwestern population remained stable after the split. This result suggests a population decline in the southwestern during the last few hundred generations, which might correspond to major episodes of deforestation and dead wood shortage (Grove 2002). At the time of ca. 1000 ad, deforestation had resulted in the almost complete loss of primary forests in central Europe, while larger extents of primary forest had remained in the boreal area and still remain in Finland today (Sabatini et al. 2021). Thus, the southwestern European population might have additionally been affected by a reduction in dead wood.

### 4.4 Concluding Remarks

We examined the population genomics of a rare and common fungal species across 13 European countries. Our findings indicate moderate support for a southwestern and northeastern population for the common polypore *Fomitopsis pinicola*, and weak, nearly absent structuring for the rare polypore *Antrodiella citrinella* across Europe. Our study suggests that postglacial population structuring is weaker than for many plant and mammal species, likely because fungal species differ considerably in their demography even across the same geographic range. We hypothesise that differences in demographic histories between fungal species result from a combination of their host relation and dispersal limitation due to differences in spore size. We recommend that conservation strategies for wood‐inhabiting fungi should consider continental‐scale strategies to maintain their genetic diversity.

## Author Contributions

C.B.: conceptualisation. F.‐S.K., M.S.: formal analysis. C.B., J.H., H.K., E.B.: methodology. J.H., K.H., E.B., A.A., D.D., J.H., R.I., K.J., I.K.‐G., V.K., S.M., J.M., A.M., O.M., K.R., P.S., Z.T., P.V., H.V., M.Z.: investigation. All authors: data curation. F.‐S.K., C.B.: writing – original draft. All authors: writing – review and editing. H.K., V.K., A.M., Z.T., R.I., K.R., J.H., M.W., C.B., F.‐S.K.: funding acquisition.

## Funding

The study was funded via the EU Program for Cross‐Border Cooperation Bavaria—Czech Republic, Objective ‘ETZ 2014–2020’ (Funga of the Bohemian Forest). Harald Kellner received funding through BMBF project CEFOX II (Grant 031B1346B). The research of Vladimír Kunca was supported by the Scientific Grant Agency of the Ministry of Education of the Slovak Republic VEGA (Grant 1/0197/24). Armin Mešić and Zdenko Tkalčec were supported by the Croatian Science Foundation for project ForFungiDNA (Grant HRZZ‐IP‐2018‐01‐1736). Reda Iršėnaitė was supported by the Mohamed bin Zayed Species Conservation Fund (Grant 202524466). Kadri Runnel was supported by the Estonian Research Council (grant PSG825 and TK232). Jan Holec was supported by the Ministry of Culture of the Czech Republic (Grant DKRVO 2024‐2028/3.I.b, 00023272). Max Zibold was supported by the Stiftung Landesbank Baden‐Württemberg (LBBW). Franz Krah and Max Zibold were supported by the Bundesministerium für Bildung und Forschung and BIODIVERSA+ (Grant 16LW0490K).

## Conflicts of Interest

The authors declare no conflicts of interest.

## Supporting information


**Data S1:** Supporting Figures and Tables.

**Supporting Information: S1** Read Alignment (BWA) results for *Antrodiella citrinella*.

**Supporting Information: S2** Read Alignment (BWA) results for *Fomitopsis pinicola*.

**Supporting Information: S3** Bioinformatics pipeline.

**Supporting Information: S4** Individual sample‐based summary statistics and NCBI Sequence Read Archive (SRA) metadata for each sample.

## Data Availability

The raw sequence reads have been made available on NCBI SRA at BioProject‐ID PRJNA1222407. R code and data for the main results and figures are provided by the DRYAD repository (https://doi.org/10.5061/dryad.dr7sqvbcd).
