# Supplementary material for: Population Genomics of a Rare and a Common Wood–Inhabiting Fungal Species Across Europe
Source: Mol Ecol. 2026 Feb 6;35(3):e70260. doi: 10.1111/mec.70260 (PMC12878558; doi:10.1111/mec.70260)
Supplement: Supplementary file 1 — Data S1: Supporting Figures and Tables. [file MEC-35-e70260-s003.pdf]

MOLECULAR ECOLOGY

**Supplemental Information for:**

# Population genomics of a rare and a common wood-inhabiting fungal species across Europe

Franz-Sebastian Krah, Mathias Scharmann, Alfons R. Weig, Jaqueline Hess, Harald Kellner, Antonis Athanasiadis, Enrico Büttner, Daniel Dvořák, Jan Holec, Reda Iršėnaitė, Kaisa Juninnen, Irmgard Krisai-Greilhuber, Vladimír Kunca, Sundy Maurice, Johannes Meier, Armin Mešić, Otto Miettinen, Kadri Runnel, Pablo Schäfer, Zdenko Tkalčec, Václav Pouska, Hermann Voglmayr, Max Zibold, Claus Bässler

## Supplementary Material

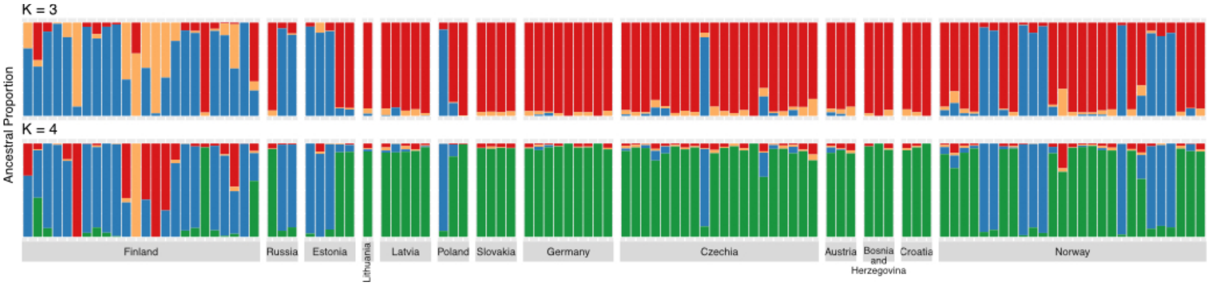


**Fig. S1** ADMIXTURE model ancestral proportion for K = 3, and 4 for *Antrodiella citrinella*.

MOLECULAR ECOLOGY

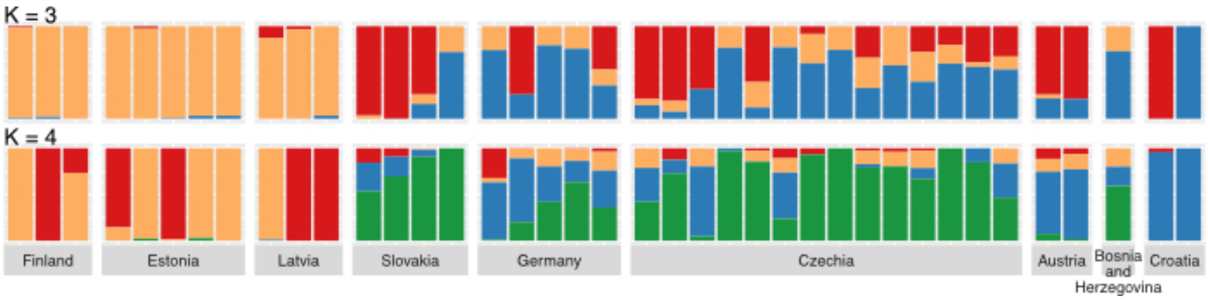


**Fig. S2** ADMIXTURE model ancestral proportion for K = 3 and 4 for *Fomitopsis pinicola*.

MOLECULAR ECOLOGY

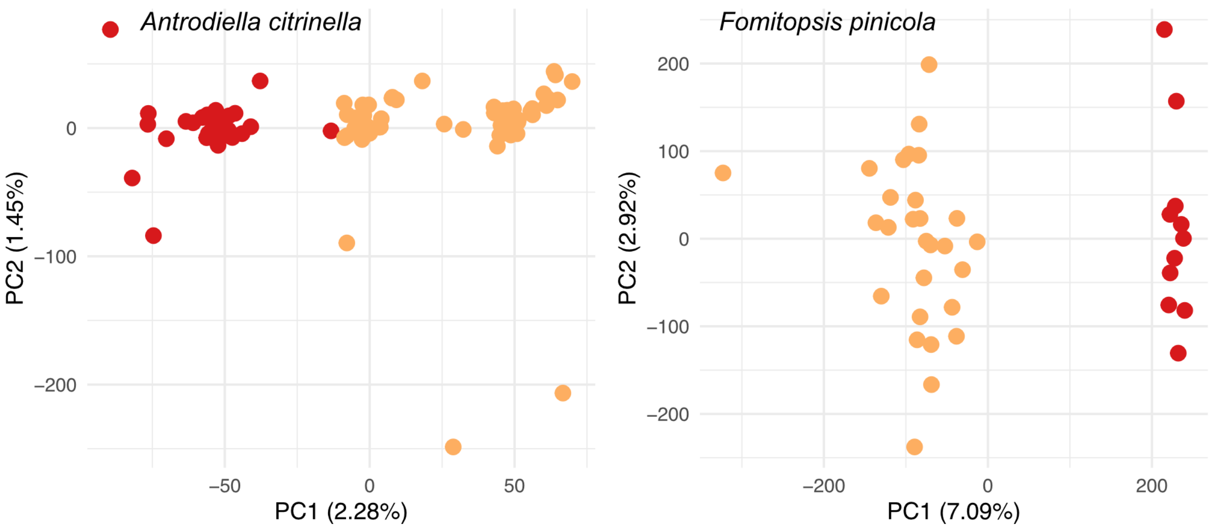


**Fig. S3** Principal component analyses using smart_pca with K = 2 clusters as color coding for a SNP dataset where 10% missing data were allowed. Note that the three clusters in *Antrodiella citrinella* are currently unclear and might be due to a supergene, which might reflect three clusters, with each cluster representing a homozygote (1/1 or 0/0) and heterozygote (0/1) as was reported for a plant species (Jay et al., 2021).

MOLECULAR ECOLOGY

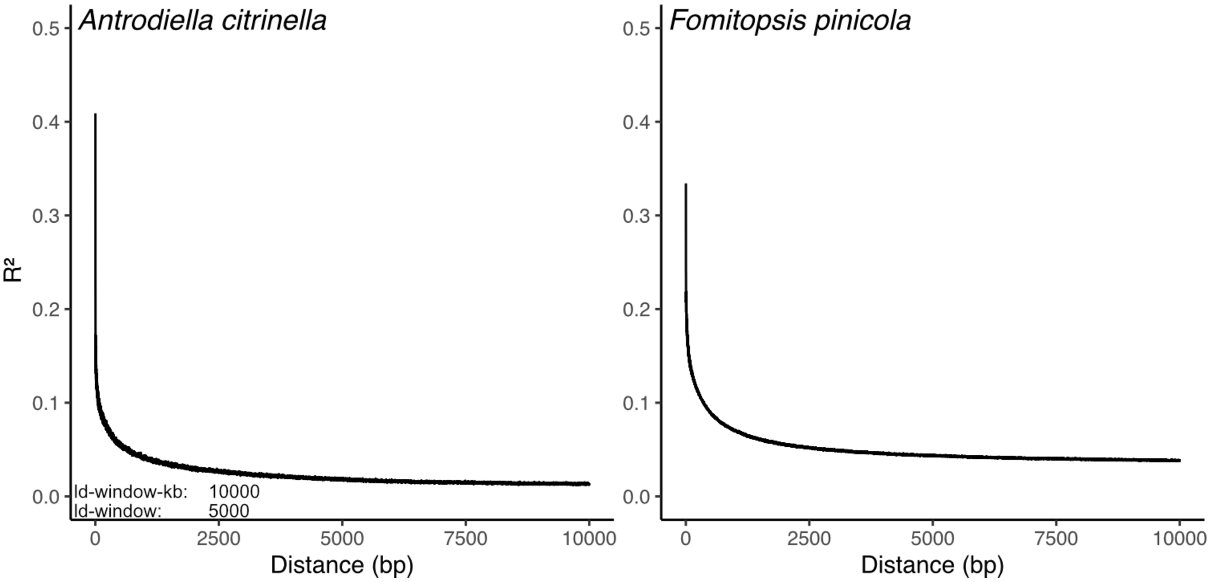


**Fig. S4** Average linkage disequilibrium plotted against distance between SNPs across the contigs for the two species.

MOLECULAR ECOLOGY

**Table S1** Overall and country-based summary statistics. Ac = *Antrodiella citrinella*, Fp = *Fomitopsis pinicola*. N SNPs = Number of single nucleotide polymorphisms; DP = depth of coverage; N individuals = Number of samples.

| Species | Country | N SNPs | Mean DP | N individuals |
|---|---|---|---|---|
| Ac | | 662,246 ± 111,342 | 32 ± 18 | 110 |
| Fp | | 1,836,778 ± 225,347 | 17 ± 9 | 39 |
| Ac | Austria | 691,802 ± 1,239 | 23 ± 4 | 3 |
| | Bosnia and Herzegovina | 693,358 ± 476 | 24 ± 3 | 3 |
| | Croatia | 672,482 ± 32,515 | 17 ± 9 | 3 |
| | Czechia | 640,259 ± 141,819 | 29 ± 20 | 20 |
| | Estonia | 688,070 ± 11,061 | 30 ± 8 | 5 |
| | Finland | 640,809 ± 156,001 | 23 ± 14 | 24 |
| | Germany | 692,206 ± 1,308 | 42 ± 16 | 9 |
| | Latvia | 692,967 ± 803 | 45 ± 18 | 5 |
| | Lithuania | 692,794 ± NA | 44 ± NA | 1 |
| | Norway | 659,297 ± 116,706 | 34 ± 17 | 27 |
| | Poland | 692,221 ± 1,495 | 44 ± 31 | 3 |
| | Russia | 672,666 ± 34,318 | 27 ± 20 | 3 |
| | Slovakia | 691,508 ± 1,478 | 65 ± 20 | 4 |
| Fp | Austria | 1,901,528 ± 2,174 | 20 ± 2 | 2 |
| | Bosnia and Herzegovina | 1,900,009 ± NA | 17 | 1 |
| | Croatia | 1,392,476 ± 713,916 | 13 ± 11 | 2 |
| | Czechia | 1,811,759 ± 269,499 | 19 ± 13 | 14 |
| | Estonia | 1,888,348 ± 14,102 | 15 ± 5 | 5 |
| | Finland | 1,898,791 ± 3,281 | 17 ± 4 | 3 |
| | Germany | 1,886,493 ± 16,773 | 12 ± 3 | 5 |
| | Latvia | 1,892,359 ± 14,890 | 16 ± 5 | 3 |
| | Slovakia | 1,883,514 ± 27,585 | 17 ± 8 | 4 |

MOLECULAR ECOLOGY

**Table S2 Redundancy analysis (RDA) showing the relationship between SNP composition and WorldClim climate variables.** The significance of the overall model and individual environmental terms was tested using permutation-based ANOVA (anova.cca).

| | Variable | Df | Variance | F value | P value |
|---|---|---|---|---|---|
| *Antrodiella citrinella* | Mean Annual Temperature | 1 | 1063,8 | 1,29 | **0,010** |
| | Mean Diurnal Range | 1 | 894,0 | 1,08 | **0,020** |
| | Isothermality | 1 | 1296,5 | 1,57 | **0,010** |
| | Mean Temperature of Wettest Quarter | 1 | 850,7 | 1,03 | 0,150 |
| | Mean Temperature of Driest Quarter | 1 | 866,0 | 1,05 | 0,110 |
| | Mean Temperature of Warmest Quarter | 1 | 991,4 | 1,20 | **0,010** |
| | Precipitation Seasonality | 1 | 925,0 | 1,12 | **0,010** |
| *Fomitopsis pinicola* | Mean annual temperature | 1 | 9385,3 | 1,1 | **0,040** |
| | Mean Diurnal Range | 1 | 9301,1 | 1,1 | 0,090 |
| | Mean Temperature of Wettest Quarter | 1 | 10767,5 | 1,3 | **0,010** |
| | Mean Temperature of Driest Quarter | 1 | 9539,0 | 1,2 | 0,070 |
| | Mean Temperature of Warmest Quarter | 1 | 16010,1 | 1,9 | **0,010** |
| | Precipitation Seasonality | 1 | 8505,8 | 1,0 | 0,300 |
| | Precipitation of Warmest Quarter | 1 | 8406,1 | 1,0 | 0,240 |
