# Supplementary material for: Population Genomics of a Rare and a Common Wood–Inhabiting Fungal Species Across Europe
Source: Mol Ecol. 2026 Feb 6;35(3):e70260. doi: 10.1111/mec.70260 (PMC12878558; doi:10.1111/mec.70260)
Supplement: Supplementary file 2 — Supporting Information: S1 Read Alignment (BWA) results for Antrodiella citrinella. [file MEC-35-e70260-s005.pdf]

Read Alignment (BWA) Results

Input 1: Reference Genome Sequences

Antrodiella_citrinella_reassembly_enz_corr

Sequences	Minimum Length	Maximum Length	Average Length	Total Length
1,228	437	546,823	26,887	33,018,101

Input 2: Sequencing Data

A total of 130 libraries have been processed.

Sample Name	Files	Sequencing	Format
AT_AT1Ac	AT_AT1Ac.F.fq.gz, AT_AT1Ac.R.fq.gz	Paired-End	FASTQ
AT_AT2Ac	AT_AT2Ac.F.fq.gz, AT_AT2Ac.R.fq.gz	Paired-End	FASTQ
AT_AT3Ac	AT_AT3Ac.F.fq.gz, AT_AT3Ac.R.fq.gz	Paired-End	FASTQ
CZ_CZ1Ac	CZ_CZ1Ac.F.fq.gz, CZ_CZ1Ac.R.fq.gz	Paired-End	FASTQ
CZ_CZ2Ac	CZ_CZ2Ac.F.fq.gz, CZ_CZ2Ac.R.fq.gz	Paired-End	FASTQ
CZ_CZ3Ac	CZ_CZ3Ac.F.fq.gz, CZ_CZ3Ac.R.fq.gz	Paired-End	FASTQ
CZ_CZ4Ac	CZ_CZ4Ac.F.fq.gz, CZ_CZ4Ac.R.fq.gz	Paired-End	FASTQ
CZ_CZ5Ac	CZ_CZ5Ac.F.fq.gz, CZ_CZ5Ac.R.fq.gz	Paired-End	FASTQ
CZ_CZ6Ac	CZ_CZ6Ac.F.fq.gz, CZ_CZ6Ac.R.fq.gz	Paired-End	FASTQ
CZ_CZ7Ac	CZ_CZ7Ac.F.fq.gz, CZ_CZ7Ac.R.fq.gz	Paired-End	FASTQ
CZ_CZ8Ac	CZ_CZ8Ac.F.fq.gz, CZ_CZ8Ac.R.fq.gz	Paired-End	FASTQ
CZ_CZ9Ac	CZ_CZ9Ac.F.fq.gz, CZ_CZ9Ac.R.fq.gz	Paired-End	FASTQ
CZ_CZ14Ac	CZ_CZ14Ac.F.fq.gz, CZ_CZ14Ac.R.fq.gz	Paired-End	FASTQ
CZ_CZ15Ac	CZ_CZ15Ac.F.fq.gz, CZ_CZ15Ac.R.fq.gz	Paired-End	FASTQ
CZ_CZ16Ac	CZ_CZ16Ac.F.fq.gz, CZ_CZ16Ac.R.fq.gz	Paired-End	FASTQ
CZ_CZ17Ac	CZ_CZ17Ac.F.fq.gz, CZ_CZ17Ac.R.fq.gz	Paired-End	FASTQ
CZ_CZ18Ac	CZ_CZ18Ac.F.fq.gz, CZ_CZ18Ac.R.fq.gz	Paired-End	FASTQ
CZ_CZ19Ac	CZ_CZ19Ac.F.fq.gz, CZ_CZ19Ac.R.fq.gz	Paired-End	FASTQ
CZ_CZ20Ac	CZ_CZ20Ac.F.fq.gz, CZ_CZ20Ac.R.fq.gz	Paired-End	FASTQ
CZ_CZ21Ac	CZ_CZ21Ac.F.fq.gz, CZ_CZ21Ac.R.fq.gz	Paired-End	FASTQ
CZ_CZ22Ac	CZ_CZ22Ac.F.fq.gz, CZ_CZ22Ac.R.fq.gz	Paired-End	FASTQ
CZ_CZ23Ac	CZ_CZ23Ac.F.fq.gz, CZ_CZ23Ac.R.fq.gz	Paired-End	FASTQ
CZ_CZ24Ac	CZ_CZ24Ac.F.fq.gz, CZ_CZ24Ac.R.fq.gz	Paired-End	FASTQ
DE_DE1Ac	DE_DE1Ac.F.fq.gz, DE_DE1Ac.R.fq.gz	Paired-End	FASTQ
DE_DE2Ac	DE_DE2Ac.F.fq.gz, DE_DE2Ac.R.fq.gz	Paired-End	FASTQ
DE_DE3Ac	DE_DE3Ac.F.fq.gz, DE_DE3Ac.R.fq.gz	Paired-End	FASTQ
DE_DE4Ac	DE_DE4Ac.F.fq.gz, DE_DE4Ac.R.fq.gz	Paired-End	FASTQ
DE_DE5Ac	DE_DE5Ac.F.fq.gz, DE_DE5Ac.R.fq.gz	Paired-End	FASTQ
DE_DE6Ac	DE_DE6Ac.F.fq.gz, DE_DE6Ac.R.fq.gz	Paired-End	FASTQ
DE_DE7Ac	DE_DE7Ac.F.fq.gz, DE_DE7Ac.R.fq.gz	Paired-End	FASTQ
DE_DE8Ac	DE_DE8Ac.F.fq.gz, DE_DE8Ac.R.fq.gz	Paired-End	FASTQ
DE_DE9Ac	DE_DE9Ac.F.fq.gz, DE_DE9Ac.R.fq.gz	Paired-End	FASTQ
DE_DE10Ac	DE_DE10Ac.F.fq.gz, DE_DE10Ac.R.fq.gz	Paired-End	FASTQ
DE_DE11Ac	DE_DE11Ac.F.fq.gz, DE_DE11Ac.R.fq.gz	Paired-End	FASTQ
DE_DE12Ac	DE_DE12Ac.F.fq.gz, DE_DE12Ac.R.fq.gz	Paired-End	FASTQ
DE_DE13Ac	DE_DE13Ac.F.fq.gz, DE_DE13Ac.R.fq.gz	Paired-End	FASTQ
EE_EE1Ac	EE_EE1Ac.F.fq.gz, EE_EE1Ac.R.fq.gz	Paired-End	FASTQ
EE_EE2Ac	EE_EE2Ac.F.fq.gz, EE_EE2Ac.R.fq.gz	Paired-End	FASTQ
EE_EE3Ac	EE_EE3Ac.F.fq.gz, EE_EE3Ac.R.fq.gz	Paired-End	FASTQ

EE_EE4Ac	EE_EE4Ac.F.fq.gz, EE_EE4Ac.R.fq.gz	Paired-End	FASTQ
EE_EE5Ac	EE_EE5Ac.F.fq.gz, EE_EE5Ac.R.fq.gz	Paired-End	FASTQ
FI_FI1Ac	FI_FI1Ac.F.fq.gz, FI_FI1Ac.R.fq.gz	Paired-End	FASTQ
FI_FI2Ac	FI_FI2Ac.F.fq.gz, FI_FI2Ac.R.fq.gz	Paired-End	FASTQ
FI_FI3Ac	FI_FI3Ac.F.fq.gz, FI_FI3Ac.R.fq.gz	Paired-End	FASTQ
FI_FI4Ac	FI_FI4Ac.F.fq.gz, FI_FI4Ac.R.fq.gz	Paired-End	FASTQ
FI_FI5Ac	FI_FI5Ac.F.fq.gz, FI_FI5Ac.R.fq.gz	Paired-End	FASTQ
FI_FI6Ac	FI_FI6Ac.F.fq.gz, FI_FI6Ac.R.fq.gz	Paired-End	FASTQ
FI_FI7Ac	FI_FI7Ac.F.fq.gz, FI_FI7Ac.R.fq.gz	Paired-End	FASTQ
FI_FI8Ac	FI_FI8Ac.F.fq.gz, FI_FI8Ac.R.fq.gz	Paired-End	FASTQ
FI_FI9Ac	FI_FI9Ac.F.fq.gz, FI_FI9Ac.R.fq.gz	Paired-End	FASTQ
FI_FI10Ac	FI_FI10Ac.F.fq.gz, FI_FI10Ac.R.fq.gz	Paired-End	FASTQ
FI_FI11Ac	FI_FI11Ac.F.fq.gz, FI_FI11Ac.R.fq.gz	Paired-End	FASTQ
FI_FI12Ac	FI_FI12Ac.F.fq.gz, FI_FI12Ac.R.fq.gz	Paired-End	FASTQ
FI_FI13Ac	FI_FI13Ac.F.fq.gz, FI_FI13Ac.R.fq.gz	Paired-End	FASTQ
FI_FI14Ac	FI_FI14Ac.F.fq.gz, FI_FI14Ac.R.fq.gz	Paired-End	FASTQ
FI_FI15Ac	FI_FI15Ac.F.fq.gz, FI_FI15Ac.R.fq.gz	Paired-End	FASTQ
FI_FI16Ac	FI_FI16Ac.F.fq.gz, FI_FI16Ac.R.fq.gz	Paired-End	FASTQ
FI_FI17Ac	FI_FI17Ac.F.fq.gz, FI_FI17Ac.R.fq.gz	Paired-End	FASTQ
FI_FI19Ac	FI_FI19Ac.F.fq.gz, FI_FI19Ac.R.fq.gz	Paired-End	FASTQ
FI_FI20Ac	FI_FI20Ac.F.fq.gz, FI_FI20Ac.R.fq.gz	Paired-End	FASTQ
FI_FI21Ac	FI_FI21Ac.F.fq.gz, FI_FI21Ac.R.fq.gz	Paired-End	FASTQ
FI_FI22Ac	FI_FI22Ac.F.fq.gz, FI_FI22Ac.R.fq.gz	Paired-End	FASTQ
FI_FI23Ac	FI_FI23Ac.F.fq.gz, FI_FI23Ac.R.fq.gz	Paired-End	FASTQ
FI_FI24Ac	FI_FI24Ac.F.fq.gz, FI_FI24Ac.R.fq.gz	Paired-End	FASTQ
FI_FI25Ac	FI_FI25Ac.F.fq.gz, FI_FI25Ac.R.fq.gz	Paired-End	FASTQ
FI_FI26Ac	FI_FI26Ac.F.fq.gz, FI_FI26Ac.R.fq.gz	Paired-End	FASTQ
FI_FI27Ac	FI_FI27Ac.F.fq.gz, FI_FI27Ac.R.fq.gz	Paired-End	FASTQ
GR_GR1Ac	GR_GR1Ac.F.fq.gz, GR_GR1Ac.R.fq.gz	Paired-End	FASTQ
HR_HR1Ac	HR_HR1Ac.F.fq.gz, HR_HR1Ac.R.fq.gz	Paired-End	FASTQ
HR_HR4Ac	HR_HR4Ac.F.fq.gz, HR_HR4Ac.R.fq.gz	Paired-End	FASTQ
HR_HR5Ac	HR_HR5Ac.F.fq.gz, HR_HR5Ac.R.fq.gz	Paired-End	FASTQ
HR_HR6Ac	HR_HR6Ac.F.fq.gz, HR_HR6Ac.R.fq.gz	Paired-End	FASTQ
HR_HR7Ac	HR_HR7Ac.F.fq.gz, HR_HR7Ac.R.fq.gz	Paired-End	FASTQ
HR_HR9Ac	HR_HR9Ac.F.fq.gz, HR_HR9Ac.R.fq.gz	Paired-End	FASTQ
HR_HR12Ac	HR_HR12Ac.F.fq.gz, HR_HR12Ac.R.fq.gz	Paired-End	FASTQ
LT_LT1Ac	LT_LT1Ac.F.fq.gz, LT_LT1Ac.R.fq.gz	Paired-End	FASTQ
LT_LT2Ac	LT_LT2Ac.F.fq.gz, LT_LT2Ac.R.fq.gz	Paired-End	FASTQ
LT_LT3Ac	LT_LT3Ac.F.fq.gz, LT_LT3Ac.R.fq.gz	Paired-End	FASTQ
LT_LT4Ac	LT_LT4Ac.F.fq.gz, LT_LT4Ac.R.fq.gz	Paired-End	FASTQ
LT_LT5Ac	LT_LT5Ac.F.fq.gz, LT_LT5Ac.R.fq.gz	Paired-End	FASTQ
LT_LT6Ac	LT_LT6Ac.F.fq.gz, LT_LT6Ac.R.fq.gz	Paired-End	FASTQ
NO_NO1Ac	NO_NO1Ac.F.fq.gz, NO_NO1Ac.R.fq.gz	Paired-End	FASTQ
NO_NO2Ac	NO_NO2Ac.F.fq.gz, NO_NO2Ac.R.fq.gz	Paired-End	FASTQ
NO_NO3Ac	NO_NO3Ac.F.fq.gz, NO_NO3Ac.R.fq.gz	Paired-End	FASTQ
NO_NO4Ac	NO_NO4Ac.F.fq.gz, NO_NO4Ac.R.fq.gz	Paired-End	FASTQ
NO_NO5Ac	NO_NO5Ac.F.fq.gz, NO_NO5Ac.R.fq.gz	Paired-End	FASTQ
NO_NO6Ac	NO_NO6Ac.F.fq.gz, NO_NO6Ac.R.fq.gz	Paired-End	FASTQ
NO_NO7Ac	NO_NO7Ac.F.fq.gz, NO_NO7Ac.R.fq.gz	Paired-End	FASTQ
NO_NO8Ac	NO_NO8Ac.F.fq.gz, NO_NO8Ac.R.fq.gz	Paired-End	FASTQ
NO_NO9Ac	NO_NO9Ac.F.fq.gz, NO_NO9Ac.R.fq.gz	Paired-End	FASTQ
NO_NO10Ac	NO_NO10Ac.F.fq.gz, NO_NO10Ac.R.fq.gz	Paired-End	FASTQ

NO_NO11Ac	NO_NO11Ac.F.fq.gz, NO_NO11Ac.R.fq.gz	Paired-End	FASTQ
NO_NO12Ac	NO_NO12Ac.F.fq.gz, NO_NO12Ac.R.fq.gz	Paired-End	FASTQ
NO_NO13Ac	NO_NO13Ac.F.fq.gz, NO_NO13Ac.R.fq.gz	Paired-End	FASTQ
NO_NO14Ac	NO_NO14Ac.F.fq.gz, NO_NO14Ac.R.fq.gz	Paired-End	FASTQ
NO_NO15Ac	NO_NO15Ac.F.fq.gz, NO_NO15Ac.R.fq.gz	Paired-End	FASTQ
NO_NO16Ac	NO_NO16Ac.F.fq.gz, NO_NO16Ac.R.fq.gz	Paired-End	FASTQ
NO_NO17Ac	NO_NO17Ac.F.fq.gz, NO_NO17Ac.R.fq.gz	Paired-End	FASTQ
NO_NO18Ac	NO_NO18Ac.F.fq.gz, NO_NO18Ac.R.fq.gz	Paired-End	FASTQ
NO_NO19Ac	NO_NO19Ac.F.fq.gz, NO_NO19Ac.R.fq.gz	Paired-End	FASTQ
NO_NO20Ac	NO_NO20Ac.F.fq.gz, NO_NO20Ac.R.fq.gz	Paired-End	FASTQ
NO_NO21Ac	NO_NO21Ac.F.fq.gz, NO_NO21Ac.R.fq.gz	Paired-End	FASTQ
NO_NO22Ac	NO_NO22Ac.F.fq.gz, NO_NO22Ac.R.fq.gz	Paired-End	FASTQ
NO_NO23Ac	NO_NO23Ac.F.fq.gz, NO_NO23Ac.R.fq.gz	Paired-End	FASTQ
NO_NO24Ac	NO_NO24Ac.F.fq.gz, NO_NO24Ac.R.fq.gz	Paired-End	FASTQ
NO_NO25Ac	NO_NO25Ac.F.fq.gz, NO_NO25Ac.R.fq.gz	Paired-End	FASTQ
NO_NO27Ac	NO_NO27Ac.F.fq.gz, NO_NO27Ac.R.fq.gz	Paired-End	FASTQ
NO_NO28Ac	NO_NO28Ac.F.fq.gz, NO_NO28Ac.R.fq.gz	Paired-End	FASTQ
NO_NO29Ac	NO_NO29Ac.F.fq.gz, NO_NO29Ac.R.fq.gz	Paired-End	FASTQ
NO_NO30Ac	NO_NO30Ac.F.fq.gz, NO_NO30Ac.R.fq.gz	Paired-End	FASTQ
NO_NO31Ac	NO_NO31Ac.F.fq.gz, NO_NO31Ac.R.fq.gz	Paired-End	FASTQ
NO_NO32Ac	NO_NO32Ac.F.fq.gz, NO_NO32Ac.R.fq.gz	Paired-End	FASTQ
NO_NO33Ac	NO_NO33Ac.F.fq.gz, NO_NO33Ac.R.fq.gz	Paired-End	FASTQ
NO_NO34Ac	NO_NO34Ac.F.fq.gz, NO_NO34Ac.R.fq.gz	Paired-End	FASTQ
NO_NO35Ac	NO_NO35Ac.F.fq.gz, NO_NO35Ac.R.fq.gz	Paired-End	FASTQ
PL_PL1Ac	PL_PL1Ac.F.fq.gz, PL_PL1Ac.R.fq.gz	Paired-End	FASTQ
PL_PL2Ac	PL_PL2Ac.F.fq.gz, PL_PL2Ac.R.fq.gz	Paired-End	FASTQ
PL_PL3Ac	PL_PL3Ac.F.fq.gz, PL_PL3Ac.R.fq.gz	Paired-End	FASTQ
RU_RU1Ac	RU_RU1Ac.F.fq.gz, RU_RU1Ac.R.fq.gz	Paired-End	FASTQ
RU_RU2Ac	RU_RU2Ac.F.fq.gz, RU_RU2Ac.R.fq.gz	Paired-End	FASTQ
RU_RU3Ac	RU_RU3Ac.F.fq.gz, RU_RU3Ac.R.fq.gz	Paired-End	FASTQ
RU_RU4Ac	RU_RU4Ac.F.fq.gz, RU_RU4Ac.R.fq.gz	Paired-End	FASTQ
RU_RU5Ac	RU_RU5Ac.F.fq.gz, RU_RU5Ac.R.fq.gz	Paired-End	FASTQ
RU_RU6Ac	RU_RU6Ac.F.fq.gz, RU_RU6Ac.R.fq.gz	Paired-End	FASTQ
SK_SK1Ac	SK_SK1Ac.F.fq.gz, SK_SK1Ac.R.fq.gz	Paired-End	FASTQ
SK_SK2Ac	SK_SK2Ac.F.fq.gz, SK_SK2Ac.R.fq.gz	Paired-End	FASTQ
SK_SK3Ac	SK_SK3Ac.F.fq.gz, SK_SK3Ac.R.fq.gz	Paired-End	FASTQ
SK_SK4Ac	SK_SK4Ac.F.fq.gz, SK_SK4Ac.R.fq.gz	Paired-End	FASTQ
SK_SK5Ac	SK_SK5Ac.F.fq.gz, SK_SK5Ac.R.fq.gz	Paired-End	FASTQ
US_US1Ac	US_US1Ac.F.fq.gz, US_US1Ac.R.fq.gz	Paired-End	FASTQ

Results Overview

Globals

Sample	Total Alignments	Mapped	Supplementary	Unmapped	Duplicated Reads (estimated)	Duplication Rate
AT_AT2Ac	6,424,582	6,085,403 / 94.721%	45,050 / 0.701%	339,179 / 5.279%	2,238,776 / 34.847%	31.28
AT_AT3Ac	8,269,480	8,128,253 / 98.292%	75,862 / 0.917%	141,227 / 1.708%	3,436,822 / 41.56%	34.43
AT_AT1Ac	18,802,867	5,608,075 / 29.826%	40,179 / 0.214%	13,194,792 / 70.174%	2,393,393 / 12.729%	30.29
CZ_CZ1Ac	13,838,797	3,133,570 / 22.643%	24,443 / 0.177%	10,705,227 / 77.357%	1,023,756 / 7.398%	27.66

CZ_CZ4Ac	389,884	331,739 / 85.087%	2,420 / 0.621%	58,145 / 14.913%	65,334 / 16.757%	18.59
CZ_CZ2Ac	12,671,698	7,663,040 / 60.474%	58,000 / 0.458%	5,008,658 / 39.526%	3,371,128 / 26.604%	34.18
CZ_CZ3Ac	16,268,919	15,941,178 / 97.985%	117,077 / 0.72%	327,741 / 2.015%	10,054,106 / 61.799%	58.63
CZ_CZ5Ac	14,544,512	13,966,908 / 96.029%	89,752 / 0.617%	577,604 / 3.971%	7,699,778 / 52.939%	41.43
CZ_CZ6Ac	12,337,908	9,147,466 / 74.141%	70,844 / 0.574%	3,190,442 / 25.859%	5,893,226 / 47.765%	61.03
CZ_CZ7Ac	12,692,452	2,542,491 / 20.032%	17,786 / 0.14%	10,149,961 / 79.968%	838,710 / 6.608%	26.87
CZ_CZ8Ac	10,276,410	6,248,004 / 60.799%	46,458 / 0.452%	4,028,406 / 39.201%	2,402,578 / 23.38%	33.77
CZ_CZ9Ac	15,483,738	14,243,125 / 91.988%	101,950 / 0.658%	1,240,613 / 8.012%	7,820,062 / 50.505%	50.31
CZ_CZ14Ac	15,458,932	10,619,789 / 68.697%	81,826 / 0.529%	4,839,143 / 31.303%	4,742,999 / 30.681%	36.92
CZ_CZ16Ac	11,910,592	8,152,809 / 68.45%	68,136 / 0.572%	3,757,783 / 31.55%	3,400,580 / 28.551%	33.66
CZ_CZ15Ac	22,437,189	16,370,793 / 72.963%	111,487 / 0.497%	6,066,396 / 27.037%	8,708,991 / 38.815%	42.38
CZ_CZ18Ac	7,395,948	2,376,552 / 32.133%	28,906 / 0.391%	5,019,396 / 67.867%	998,979 / 13.507%	44.04
CZ_CZ17Ac	12,691,365	1,066,150 / 8.401%	8,157 / 0.064%	11,625,215 / 91.599%	283,383 / 2.233%	23.19
CZ_CZ19Ac	17,523,769	9,785,764 / 55.843%	73,429 / 0.419%	7,738,005 / 44.157%	4,376,653 / 24.976%	36.06
CZ_CZ20Ac	15,566,303	7,061,546 / 45.364%	53,665 / 0.345%	8,504,757 / 54.636%	2,804,930 / 18.019%	32.68
CZ_CZ21Ac	13,694,629	4,731,691 / 34.551%	38,519 / 0.281%	8,962,938 / 65.449%	1,816,715 / 13.266%	29.33
CZ_CZ22Ac	19,394,500	3,353,660 / 17.292%	24,332 / 0.125%	16,040,840 / 82.708%	1,069,114 / 5.512%	26.14
CZ_CZ24Ac	10,998,209	4,445,780 / 40.423%	32,819 / 0.298%	6,552,429 / 59.577%	1,424,945 / 12.956%	25.52
DE_DE1Ac	11,610,375	374,211 / 3.223%	2,585 / 0.022%	11,236,164 / 96.777%	323,967 / 2.79%	69.95
CZ_CZ23Ac	19,813,159	17,269,736 / 87.163%	131,245 / 0.662%	2,543,423 / 12.837%	9,291,646 / 46.896%	43.5
DE_DE2Ac	8,726,253	7,096,030 / 81.318%	56,639 / 0.649%	1,630,223 / 18.682%	2,801,528 / 32.105%	34.44
DE_DE3Ac	9,461,351	9,253,685 / 97.805%	82,493 / 0.872%	207,666 / 2.195%	3,844,625 / 40.635%	35.26
DE_DE4Ac	11,670,848	11,278,973 / 96.642%	107,750 / 0.923%	391,875 / 3.358%	5,230,466 / 44.817%	39.39
DE_DE5Ac	12,335,616	11,598,468 / 94.024%	95,798 / 0.777%	737,148 / 5.976%	5,377,763 / 43.595%	37.53
DE_DE6Ac	12,664,973	11,277,618 / 89.046%	93,407 / 0.738%	1,387,355 / 10.954%	5,052,636 / 39.895%	36.71
DE_DE7Ac	14,228,219	468,008 / 3.289%	3,347 / 0.024%	13,760,211 / 96.711%	410,523 / 2.885%	69.12
DE_DE8Ac	10,174,201	9,797,962 / 96.302%	75,841 / 0.745%	376,239 / 3.698%	4,205,475 / 41.335%	35.79
DE_DE9Ac	19,953,242	106,577 / 0.534%	1,018 / 0.005%	19,846,665 / 99.466%	72,732 / 0.365%	88.73
DE_DE10Ac	23,145,727	22,852,881 / 98.735%	171,003 / 0.739%	292,846 / 1.265%	13,523,357 / 58.427%	48.44
DE_DE11Ac	14,611,125	14,422,820 / 98.711%	107,887 / 0.738%	188,305 / 1.289%	7,287,869 / 49.879%	41.61
DE_DE12Ac	16,056,516	434,566 / 2.706%	3,376 / 0.021%	15,621,950 / 97.294%	380,788 / 2.372%	68.37

EE_EE1Ac	11,608,057	11,127,578 / 95.861%	90,565 / 0.78%	480,479 / 4.139%	5,018,156 / 43.23%	38.03
EE_EE2Ac	9,638,668	7,993,570 / 82.932%	56,182 / 0.583%	1,645,098 / 17.068%	6,627,821 / 68.763%	89.19
DE_DE13Ac	19,844,183	10,831,396 / 54.582%	84,375 / 0.425%	9,012,787 / 45.418%	5,035,934 / 25.377%	37.21
EE_EE3Ac	10,005,322	9,066,166 / 90.613%	68,194 / 0.682%	939,156 / 9.387%	3,669,535 / 36.676%	34.89
EE_EE4Ac	15,451,052	7,818,147 / 50.599%	57,024 / 0.369%	7,632,905 / 49.401%	3,324,125 / 21.514%	33.64
EE_EE5Ac	13,220,276	6,842,922 / 51.761%	53,516 / 0.405%	6,377,354 / 48.239%	2,633,215 / 19.918%	33.15
FI_FI1Ac	16,694,250	12,940,740 / 77.516%	96,000 / 0.575%	3,753,510 / 22.484%	6,148,423 / 36.83%	39.38
FI_FI2Ac	25,353,376	16,073,530 / 63.398%	121,934 / 0.481%	9,279,846 / 36.602%	8,447,445 / 33.319%	42.69
FI_FI3Ac	17,953,537	8,569,549 / 47.732%	65,835 / 0.367%	9,383,988 / 52.268%	3,628,440 / 20.21%	34.64
FI_FI4Ac	11,039,483	8,549,595 / 77.446%	59,375 / 0.538%	2,489,888 / 22.554%	3,470,179 / 31.434%	32.41
FI_FI5Ac	9,902,262	8,728,869 / 88.15%	74,646 / 0.754%	1,173,393 / 11.85%	4,097,261 / 41.377%	41.78
FI_FI6Ac	6,354,718	5,682,377 / 89.42%	43,476 / 0.684%	672,341 / 10.58%	2,166,695 / 34.096%	33.31
FI_FI7Ac	6,903,535	2,713,933 / 39.312%	20,399 / 0.295%	4,189,602 / 60.688%	1,042,378 / 15.099%	37.54
FI_FI9Ac	1,243,171	677,052 / 54.462%	4,161 / 0.335%	566,119 / 45.538%	664,509 / 53.453%	85.22
FI_FI8Ac	7,709,694	5,015,844 / 65.059%	35,106 / 0.455%	2,693,850 / 34.941%	1,913,945 / 24.825%	33.43
FI_FI11Ac	5,616,196	921,136 / 16.401%	6,312 / 0.112%	4,695,060 / 83.599%	297,829 / 5.303%	27.18
FI_FI10Ac	13,051,844	12,884,088 / 98.715%	91,692 / 0.703%	167,756 / 1.285%	6,555,648 / 50.228%	40.57
FI_FI12Ac	7,882,946	4,911,775 / 62.309%	36,136 / 0.458%	2,971,171 / 37.691%	1,934,949 / 24.546%	34.26
FI_FI13Ac	7,549,045	6,182,947 / 81.904%	47,017 / 0.623%	1,366,098 / 18.096%	2,358,265 / 31.239%	33.62
FI_FI14Ac	7,832,664	5,179,983 / 66.133%	43,416 / 0.554%	2,652,681 / 33.867%	1,934,635 / 24.7%	33.87
FI_FI15Ac	8,589,840	7,323,205 / 85.254%	61,656 / 0.718%	1,266,635 / 14.746%	3,014,951 / 35.099%	35.22
FI_FI16Ac	5,175,219	3,745,970 / 72.383%	28,751 / 0.556%	1,429,249 / 27.617%	1,297,066 / 25.063%	32.59
FI_FI17Ac	4,788,748	2,467,146 / 51.52%	18,770 / 0.392%	2,321,602 / 48.48%	929,437 / 19.409%	34.48
FI_FI19Ac	8,297,653	4,983,621 / 60.061%	36,181 / 0.436%	3,314,032 / 39.939%	1,879,392 / 22.65%	32.68
FI_FI21Ac	6,958,971	161,092 / 2.315%	1,157 / 0.017%	6,797,879 / 97.685%	139,681 / 2.007%	88.59
FI_FI20Ac	9,614,585	9,475,362 / 98.552%	69,069 / 0.718%	139,223 / 1.448%	4,271,741 / 44.43%	37.01
FI_FI23Ac	6,907,033	6,798,053 / 98.422%	54,003 / 0.782%	108,980 / 1.578%	2,770,368 / 40.109%	35.87
FI_FI24Ac	5,061,945	234,795 / 4.638%	891 / 0.018%	4,827,150 / 95.362%	191,513 / 3.783%	73.16
FI_FI25Ac	7,993,731	7,882,530 / 98.609%	60,379 / 0.755%	111,201 / 1.391%	3,585,248 / 44.851%	36.1
FI_FI22Ac	14,191,813	13,954,676 / 98.329%	93,201 / 0.657%	237,137 / 1.671%	7,567,634 / 53.324%	41.05
FI_FI26Ac	6,034,951	5,955,085 / 98.677%	41,161 / 0.682%	79,866 / 1.323%	2,524,833 / 41.837%	32.4

HR_HR1Ac	879	252 / 28.669%	1 / 0.114%	627 / 71.331%	15 / 1.706%	6.33
FI_FI27Ac	8,179,653	8,062,109 / 98.563%	57,559 / 0.704%	117,544 / 1.437%	3,544,301 / 43.331%	35.51
HR_HR4Ac	5,529,819	3,295,277 / 59.591%	17,931 / 0.324%	2,234,542 / 40.409%	1,487,913 / 26.907%	45.22
GR_GR1Ac	10,671,694	472,512 / 4.428%	3,252 / 0.03%	10,199,182 / 95.572%	411,983 / 3.861%	64.61
HR_HR5Ac	6,723,500	6,341,669 / 94.321%	38,644 / 0.575%	381,831 / 5.679%	3,362,191 / 50.007%	38.86
HR_HR6Ac	8,748,753	8,046,796 / 91.976%	61,743 / 0.706%	701,957 / 8.024%	3,656,457 / 41.794%	41.3
HR_HR7Ac	8,001,852	6,005,477 / 75.051%	42,958 / 0.537%	1,996,375 / 24.949%	2,334,382 / 29.173%	33.97
HR_HR9Ac	8,227,004	7,772,874 / 94.48%	53,292 / 0.648%	454,130 / 5.52%	3,261,139 / 39.639%	37.03
HR_HR12Ac	9,724,591	7,207,223 / 74.113%	52,269 / 0.537%	2,517,368 / 25.887%	3,083,979 / 31.713%	36.39
LT_LT1Ac	12,599,212	11,878,228 / 94.278%	85,890 / 0.682%	720,984 / 5.722%	5,319,017 / 42.217%	38.43
LT_LT3Ac	14,692,832	11,157,811 / 75.941%	93,794 / 0.638%	3,535,021 / 24.059%	4,794,383 / 32.631%	37.32
LT_LT2Ac	25,075,665	20,696,292 / 82.535%	156,449 / 0.624%	4,379,373 / 17.465%	11,299,071 / 45.06%	45.87
LT_LT4Ac	16,644,063	8,457,484 / 50.814%	67,299 / 0.404%	8,186,579 / 49.186%	3,534,308 / 21.235%	33.53
LT_LT6Ac	10,601,288	8,668,958 / 81.773%	62,544 / 0.59%	1,932,330 / 18.227%	3,699,477 / 34.896%	33.5
LT_LT5Ac	18,543,917	12,961,998 / 69.899%	96,887 / 0.522%	5,581,919 / 30.101%	6,320,538 / 34.084%	40.11
NO_NO1Ac	11,465,900	10,345,698 / 90.23%	78,704 / 0.686%	1,120,202 / 9.77%	4,429,882 / 38.635%	36.88
NO_NO3Ac	9,214,148	81,428 / 0.884%	390 / 0.004%	9,132,720 / 99.116%	35,163 / 0.382%	44.3
NO_NO2Ac	14,892,337	227,194 / 1.526%	1,281 / 0.009%	14,665,143 / 98.474%	73,069 / 0.491%	30.05
NO_NO5Ac	7,353,924	5,185,583 / 70.515%	43,028 / 0.585%	2,168,341 / 29.485%	1,826,120 / 24.832%	30.16
NO_NO4Ac	8,454,932	2,101,599 / 24.856%	16,732 / 0.198%	6,353,333 / 75.144%	561,044 / 6.636%	23.54
NO_NO7Ac	7,504,812	4,283,672 / 57.079%	32,948 / 0.439%	3,221,140 / 42.921%	1,500,641 / 19.996%	28.82
NO_NO6Ac	18,201,493	5,265,633 / 28.93%	41,235 / 0.227%	12,935,860 / 71.07%	1,906,227 / 10.473%	30.57
NO_NO8Ac	10,716,030	8,921,949 / 83.258%	70,040 / 0.654%	1,794,081 / 16.742%	3,693,243 / 34.465%	35.29
NO_NO9Ac	13,560,839	13,253,665 / 97.735%	98,375 / 0.725%	307,174 / 2.265%	6,683,524 / 49.285%	37.92
NO_NO11Ac	10,360,453	987,363 / 9.53%	8,263 / 0.08%	9,373,090 / 90.47%	293,481 / 2.833%	25.79
NO_NO10Ac	12,615,949	12,022,587 / 95.297%	102,493 / 0.812%	593,362 / 4.703%	5,538,113 / 43.898%	38.02
NO_NO12Ac	11,835,656	9,563,268 / 80.8%	81,226 / 0.686%	2,272,388 / 19.2%	4,199,321 / 35.48%	35.5
NO_NO13Ac	9,147,485	6,963,804 / 76.128%	62,485 / 0.683%	2,183,681 / 23.872%	2,636,946 / 28.827%	32.86
NO_NO14Ac	8,844,106	4,118,617 / 46.569%	33,288 / 0.376%	4,725,489 / 53.431%	1,518,225 / 17.167%	29.28
NO_NO15Ac	11,559,233	9,076,226 / 78.519%	72,859 / 0.63%	2,483,007 / 21.481%	3,937,972 / 34.068%	35.77
NO_NO16Ac	21,369,044	12,754,814 / 59.688%	100,452 / 0.47%	8,614,230 / 40.312%	6,019,859 / 28.171%	41.45

NO_NO17Ac	19,806,382	16,112,267 / 81.349%	131,728 / 0.665%	3,694,115 / 18.651%	8,099,014 / 40.891%	42.56
NO_NO18Ac	19,100,775	99,087 / 0.519%	383 / 0.002%	19,001,688 / 99.481%	76,184 / 0.399%	63.81
NO_NO19Ac	18,251,407	12,177,739 / 66.722%	95,537 / 0.523%	6,073,668 / 33.278%	5,698,803 / 31.224%	39.32
NO_NO20Ac	18,787,113	8,353,038 / 44.462%	67,205 / 0.358%	10,434,075 / 55.538%	3,383,777 / 18.011%	34.64
NO_NO21Ac	15,507,491	10,836,016 / 69.876%	84,211 / 0.543%	4,671,475 / 30.124%	4,875,375 / 31.439%	38
NO_NO22Ac	12,163,020	9,956,136 / 81.856%	80,552 / 0.662%	2,206,884 / 18.144%	4,434,401 / 36.458%	36.52
NO_NO23Ac	17,219,896	143,283 / 0.832%	1,984 / 0.012%	17,076,613 / 99.168%	85,411 / 0.496%	45.27
NO_NO24Ac	15,207,871	57,339 / 0.377%	443 / 0.003%	15,150,532 / 99.623%	26,866 / 0.177%	43.74
NO_NO25Ac	17,473,148	12,046,874 / 68.945%	92,958 / 0.532%	5,426,274 / 31.055%	5,544,068 / 31.729%	39.29
NO_NO27Ac	14,856,795	13,004,333 / 87.531%	110,849 / 0.746%	1,852,462 / 12.469%	6,190,302 / 41.666%	39.7
NO_NO28Ac	17,620,362	17,282,568 / 98.083%	145,374 / 0.825%	337,794 / 1.917%	8,861,290 / 50.29%	43.66
NO_NO29Ac	17,254,917	14,867,637 / 86.165%	117,509 / 0.681%	2,387,280 / 13.835%	7,557,932 / 43.802%	41.53
NO_NO30Ac	12,336,502	10,853,895 / 87.982%	85,378 / 0.692%	1,482,607 / 12.018%	5,031,978 / 40.789%	36.66
NO_NO31Ac	13,705,517	10,000,606 / 72.968%	80,447 / 0.587%	3,704,911 / 27.032%	4,473,266 / 32.638%	36.29
NO_NO32Ac	20,095,666	9,018,516 / 44.878%	69,570 / 0.346%	11,077,150 / 55.122%	3,806,328 / 18.941%	35.95
NO_NO33Ac	13,722,522	11,809,758 / 86.061%	98,962 / 0.721%	1,912,764 / 13.939%	5,407,677 / 39.407%	38.55
NO_NO34Ac	30,518,104	145,283 / 0.476%	902 / 0.003%	30,372,821 / 99.524%	68,255 / 0.224%	45.11
NO_NO35Ac	22,909,141	590,092 / 2.576%	8,017 / 0.035%	22,319,049 / 97.424%	243,082 / 1.061%	36.35
PL_PL2Ac	9,122,364	8,245,540 / 90.388%	79,380 / 0.87%	876,824 / 9.612%	3,368,651 / 36.927%	33.96
PL_PL3Ac	9,681,069	8,879,540 / 91.721%	105,233 / 1.087%	801,529 / 8.279%	3,865,559 / 39.929%	39.2
PL_PL1Ac	23,321,428	22,010,548 / 94.379%	176,344 / 0.756%	1,310,880 / 5.621%	12,496,512 / 53.584%	48.24
RU_RU3Ac	421,721	97,604 / 23.144%	1,171 / 0.278%	324,117 / 76.856%	59,454 / 14.098%	67.42
RU_RU1Ac	13,721,269	11,397,746 / 83.066%	141,351 / 1.03%	2,323,523 / 16.934%	5,495,839 / 40.053%	46.38
RU_RU4Ac	3,241,569	2,560,654 / 78.994%	18,301 / 0.565%	680,915 / 21.006%	941,617 / 29.048%	32.92
RU_RU2Ac	15,549,393	13,239,124 / 85.142%	108,021 / 0.695%	2,310,269 / 14.858%	6,526,927 / 41.975%	40.87
RU_RU5Ac	8,601,823	7,396,411 / 85.987%	79,651 / 0.926%	1,205,412 / 14.013%	3,140,529 / 36.51%	35.53
RU_RU6Ac	6,900,816	5,144,212 / 74.545%	51,120 / 0.741%	1,756,604 / 25.455%	2,064,750 / 29.92%	33.89
SK_SK1Ac	26,198,898	24,381,766 / 93.064%	186,912 / 0.713%	1,817,132 / 6.936%	14,158,945 / 54.044%	49.26
SK_SK2Ac	21,954,709	17,059,821 / 77.705%	132,911 / 0.605%	4,894,888 / 22.295%	8,944,898 / 40.743%	43.23
SK_SK3Ac	18,009,392	12,095,446 / 67.162%	91,316 / 0.507%	5,913,946 / 32.838%	5,651,625 / 31.382%	37.5
US_US1Ac	4,486,526	3,239,339 / 72.201%	37,670 / 0.84%	1,247,187 / 27.799%	1,169,572 / 26.069%	30.59

SK_SK4Ac	21,049,810	16,363,782 / 77.738%	124,252 / 0.59%	4,686,028 / 22.262%	8,530,666 / 40.526%	42.6
SK_SK5Ac	17,841,637	15,873,594 / 88.969%	118,251 / 0.663%	1,968,043 / 11.031%	8,317,688 / 46.62%	42.12

Paired Information

Sample	First in pair	Second in pair	Both in pair	Singletons
AT_AT2Ac	3,046,286 / 47.416%	3,039,117 / 47.305%	6,065,013 / 94.403%	20,390 / 0.317%
AT_AT3Ac	4,071,985 / 49.241%	4,056,268 / 49.051%	8,094,081 / 97.879%	34,172 / 0.413%
AT_AT1Ac	2,807,181 / 14.93%	2,800,894 / 14.896%	5,583,762 / 29.696%	24,313 / 0.129%
CZ_CZ1Ac	1,567,998 / 11.33%	1,565,572 / 11.313%	3,096,911 / 22.378%	36,659 / 0.265%
CZ_CZ4Ac	166,153 / 42.616%	165,586 / 42.471%	330,478 / 84.763%	1,261 / 0.323%
CZ_CZ2Ac	3,837,591 / 30.285%	3,825,449 / 30.189%	7,625,055 / 60.174%	37,985 / 0.3%
CZ_CZ3Ac	7,977,155 / 49.033%	7,964,023 / 48.952%	15,883,608 / 97.632%	57,570 / 0.354%
CZ_CZ5Ac	6,990,674 / 48.064%	6,976,234 / 47.965%	13,918,148 / 95.693%	48,760 / 0.335%
CZ_CZ6Ac	4,576,953 / 37.097%	4,570,513 / 37.044%	9,115,857 / 73.885%	31,609 / 0.256%
CZ_CZ7Ac	1,274,538 / 10.042%	1,267,953 / 9.99%	2,526,690 / 19.907%	15,801 / 0.124%
CZ_CZ8Ac	3,127,004 / 30.429%	3,121,000 / 30.371%	6,225,359 / 60.579%	22,645 / 0.22%
CZ_CZ9Ac	7,133,681 / 46.072%	7,109,444 / 45.916%	14,184,917 / 91.612%	58,208 / 0.376%
CZ_CZ14Ac	5,314,609 / 34.379%	5,305,180 / 34.318%	10,582,784 / 68.457%	37,005 / 0.239%
CZ_CZ16Ac	4,080,066 / 34.256%	4,072,743 / 34.194%	8,119,957 / 68.174%	32,852 / 0.276%
CZ_CZ15Ac	8,192,634 / 36.514%	8,178,159 / 36.449%	16,314,015 / 72.71%	56,778 / 0.253%
CZ_CZ18Ac	1,189,204 / 16.079%	1,187,348 / 16.054%	2,360,498 / 31.916%	16,054 / 0.217%
CZ_CZ17Ac	533,518 / 4.204%	532,632 / 4.197%	1,060,347 / 8.355%	5,803 / 0.046%
CZ_CZ19Ac	4,902,706 / 27.977%	4,883,058 / 27.865%	9,735,391 / 55.555%	50,373 / 0.287%
CZ_CZ20Ac	3,535,390 / 22.712%	3,526,156 / 22.652%	7,033,331 / 45.183%	28,215 / 0.181%
CZ_CZ21Ac	2,371,125 / 17.314%	2,360,566 / 17.237%	4,698,773 / 34.311%	32,918 / 0.24%
CZ_CZ22Ac	1,679,137 / 8.658%	1,674,523 / 8.634%	3,336,455 / 17.203%	17,205 / 0.089%
CZ_CZ24Ac	2,225,178 / 20.232%	2,220,602 / 20.191%	4,431,057 / 40.289%	14,723 / 0.134%
DE_DE1Ac	187,645 / 1.616%	186,566 / 1.607%	320,535 / 2.761%	53,676 / 0.462%
CZ_CZ23Ac	8,650,331 / 43.66%	8,619,405 / 43.503%	17,195,097 / 86.786%	74,639 / 0.377%
DE_DE2Ac	3,552,125 / 40.706%	3,543,905 / 40.612%	7,074,328 / 81.069%	21,702 / 0.249%
DE_DE3Ac	4,630,640 / 48.943%	4,623,045 / 48.862%	9,229,100 / 97.545%	24,585 / 0.26%
DE_DE4Ac	5,643,527 / 48.356%	5,635,446 / 48.287%	11,251,695 / 96.409%	27,278 / 0.234%
DE_DE5Ac	5,804,587 / 47.056%	5,793,881 / 46.969%	11,565,277 / 93.755%	33,191 / 0.269%
DE_DE6Ac	5,643,445 / 44.559%	5,634,173 / 44.486%	11,244,332 / 88.783%	33,286 / 0.263%
DE_DE7Ac	233,909 / 1.644%	234,099 / 1.645%	410,756 / 2.887%	57,252 / 0.402%
DE_DE8Ac	4,904,414 / 48.204%	4,893,548 / 48.098%	9,766,505 / 95.993%	31,457 / 0.309%
DE_DE9Ac	53,363 / 0.267%	53,214 / 0.267%	102,777 / 0.515%	3,800 / 0.019%
DE_DE10Ac	11,437,764 / 49.416%	11,415,117 / 49.318%	22,781,303 / 98.426%	71,578 / 0.309%
DE_DE11Ac	7,217,754 / 49.399%	7,205,066 / 49.312%	14,379,876 / 98.417%	42,944 / 0.294%
DE_DE12Ac	218,276 / 1.359%	216,290 / 1.347%	358,546 / 2.233%	76,020 / 0.473%
EE_EE1Ac	5,569,738 / 47.982%	5,557,840 / 47.879%	11,087,186 / 95.513%	40,392 / 0.348%
EE_EE2Ac	4,000,437 / 41.504%	3,993,133 / 41.428%	7,977,029 / 82.761%	16,541 / 0.172%
DE_DE13Ac	5,423,630 / 27.331%	5,407,766 / 27.251%	10,778,169 / 54.314%	53,227 / 0.268%
EE_EE3Ac	4,537,603 / 45.352%	4,528,563 / 45.262%	9,033,778 / 90.29%	32,388 / 0.324%
EE_EE4Ac	3,913,237 / 25.327%	3,904,910 / 25.273%	7,786,452 / 50.394%	31,695 / 0.205%
EE_EE5Ac	3,425,933 / 25.914%	3,416,989 / 25.847%	6,812,818 / 51.533%	30,104 / 0.228%
FI_FI1Ac	6,478,424 / 38.806%	6,462,316 / 38.71%	12,883,884 / 77.176%	56,856 / 0.341%
FI_FI2Ac	8,052,767 / 31.762%	8,020,763 / 31.636%	15,989,105 / 63.065%	84,425 / 0.333%
FI_FI3Ac	4,292,593 / 23.909%	4,276,956 / 23.822%	8,524,411 / 47.48%	45,138 / 0.251%
FI_FI4Ac	4,278,788 / 38.759%	4,270,807 / 38.687%	8,521,761 / 77.193%	27,834 / 0.252%
FI_FI5Ac	4,369,645 / 44.128%	4,359,224 / 44.023%	8,704,827 / 87.907%	24,042 / 0.243%

FI_FI6Ac	2,844,584 / 44.763%	2,837,793 / 44.656%	5,666,096 / 89.164%	16,281 / 0.256%
FI_FI7Ac	1,358,330 / 19.676%	1,355,603 / 19.636%	2,707,541 / 39.22%	6,392 / 0.093%
FI_FI9Ac	339,038 / 27.272%	338,014 / 27.19%	675,262 / 54.318%	1,790 / 0.144%
FI_FI8Ac	2,510,609 / 32.564%	2,505,235 / 32.495%	5,002,334 / 64.884%	13,510 / 0.175%
FI_FI11Ac	460,859 / 8.206%	460,277 / 8.196%	919,086 / 16.365%	2,050 / 0.037%
FI_FI10Ac	6,449,007 / 49.411%	6,435,081 / 49.304%	12,844,568 / 98.412%	39,520 / 0.303%
FI_FI12Ac	2,458,688 / 31.19%	2,453,087 / 31.119%	4,897,893 / 62.133%	13,882 / 0.176%
FI_FI13Ac	3,095,583 / 41.006%	3,087,364 / 40.897%	6,164,315 / 81.657%	18,632 / 0.247%
FI_FI14Ac	2,593,608 / 33.113%	2,586,375 / 33.02%	5,162,837 / 65.914%	17,146 / 0.219%
FI_FI15Ac	3,664,598 / 42.662%	3,658,607 / 42.592%	7,305,949 / 85.053%	17,256 / 0.201%
FI_FI16Ac	1,874,856 / 36.228%	1,871,114 / 36.155%	3,737,117 / 72.212%	8,853 / 0.171%
FI_FI17Ac	1,234,876 / 25.787%	1,232,270 / 25.733%	2,460,976 / 51.391%	6,170 / 0.129%
FI_FI19Ac	2,495,569 / 30.076%	2,488,052 / 29.985%	4,965,239 / 59.839%	18,382 / 0.222%
FI_FI21Ac	80,606 / 1.158%	80,486 / 1.157%	155,735 / 2.238%	5,357 / 0.077%
FI_FI20Ac	4,746,630 / 49.369%	4,728,732 / 49.183%	9,440,876 / 98.193%	34,486 / 0.359%
FI_FI23Ac	3,403,260 / 49.272%	3,394,793 / 49.15%	6,778,791 / 98.143%	19,262 / 0.279%
FI_FI24Ac	117,636 / 2.324%	117,159 / 2.315%	217,720 / 4.301%	17,075 / 0.337%
FI_FI25Ac	3,945,640 / 49.359%	3,936,890 / 49.25%	7,859,037 / 98.315%	23,493 / 0.294%
FI_FI22Ac	6,988,703 / 49.245%	6,965,973 / 49.084%	13,908,832 / 98.006%	45,844 / 0.323%
FI_FI26Ac	2,981,875 / 49.41%	2,973,210 / 49.267%	5,935,740 / 98.356%	19,345 / 0.321%
HR_HR1Ac	126 / 14.334%	126 / 14.334%	251 / 28.555%	1 / 0.114%
FI_FI27Ac	4,038,385 / 49.371%	4,023,724 / 49.192%	8,032,865 / 98.205%	29,244 / 0.358%
HR_HR4Ac	1,649,288 / 29.825%	1,645,989 / 29.766%	3,290,283 / 59.501%	4,994 / 0.09%
GR_GR1Ac	236,484 / 2.216%	236,028 / 2.212%	430,981 / 4.039%	41,531 / 0.389%
HR_HR5Ac	3,173,381 / 47.198%	3,168,288 / 47.123%	6,329,842 / 94.145%	11,827 / 0.176%
HR_HR6Ac	4,027,635 / 46.037%	4,019,161 / 45.94%	8,023,969 / 91.716%	22,827 / 0.261%
HR_HR7Ac	3,006,915 / 37.578%	2,998,562 / 37.473%	5,986,028 / 74.808%	19,449 / 0.243%
HR_HR9Ac	3,890,287 / 47.287%	3,882,587 / 47.193%	7,751,958 / 94.226%	20,916 / 0.254%
HR_HR12Ac	3,607,378 / 37.095%	3,599,845 / 37.018%	7,185,048 / 73.885%	22,175 / 0.228%
LT_LT1Ac	5,943,684 / 47.175%	5,934,544 / 47.103%	11,842,905 / 93.997%	35,323 / 0.28%
LT_LT3Ac	5,585,230 / 38.013%	5,572,581 / 37.927%	11,113,214 / 75.637%	44,597 / 0.304%
LT_LT2Ac	10,357,395 / 41.305%	10,338,897 / 41.231%	20,625,119 / 82.252%	71,173 / 0.284%
LT_LT4Ac	4,233,649 / 25.436%	4,223,835 / 25.377%	8,418,501 / 50.58%	38,983 / 0.234%
LT_LT6Ac	4,338,790 / 40.927%	4,330,168 / 40.846%	8,641,220 / 81.511%	27,738 / 0.262%
LT_LT5Ac	6,487,638 / 34.985%	6,474,360 / 34.914%	12,913,659 / 69.638%	48,339 / 0.261%
NO_NO1Ac	5,179,679 / 45.175%	5,166,019 / 45.056%	10,309,807 / 89.917%	35,891 / 0.313%
NO_NO3Ac	40,732 / 0.442%	40,696 / 0.442%	80,405 / 0.873%	1,023 / 0.011%
NO_NO2Ac	113,664 / 0.763%	113,530 / 0.762%	225,478 / 1.514%	1,716 / 0.012%
NO_NO5Ac	2,595,178 / 35.29%	2,590,405 / 35.225%	5,168,538 / 70.283%	17,045 / 0.232%
NO_NO4Ac	1,051,910 / 12.441%	1,049,689 / 12.415%	2,092,443 / 24.748%	9,156 / 0.108%
NO_NO7Ac	2,143,982 / 28.568%	2,139,690 / 28.511%	4,269,676 / 56.893%	13,996 / 0.186%
NO_NO6Ac	2,634,910 / 14.476%	2,630,723 / 14.453%	5,248,025 / 28.833%	17,608 / 0.097%
NO_NO8Ac	4,468,881 / 41.703%	4,453,068 / 41.555%	8,881,313 / 82.879%	40,636 / 0.379%
NO_NO9Ac	6,640,113 / 48.965%	6,613,552 / 48.769%	13,197,457 / 97.32%	56,208 / 0.414%
NO_NO11Ac	494,376 / 4.772%	492,987 / 4.758%	976,129 / 9.422%	11,234 / 0.108%
NO_NO10Ac	6,019,460 / 47.713%	6,003,127 / 47.584%	11,976,522 / 94.932%	46,065 / 0.365%
NO_NO12Ac	4,786,725 / 40.443%	4,776,543 / 40.357%	9,524,406 / 80.472%	38,862 / 0.328%
NO_NO13Ac	3,485,119 / 38.099%	3,478,685 / 38.029%	6,939,871 / 75.866%	23,933 / 0.262%
NO_NO14Ac	2,060,633 / 23.3%	2,057,984 / 23.27%	4,107,222 / 46.44%	11,395 / 0.129%
NO_NO15Ac	4,543,459 / 39.306%	4,532,767 / 39.213%	9,038,694 / 78.195%	37,532 / 0.325%
NO_NO16Ac	6,393,135 / 29.918%	6,361,679 / 29.771%	12,678,350 / 59.33%	76,464 / 0.358%
NO_NO17Ac	8,077,541 / 40.783%	8,034,726 / 40.566%	16,017,287 / 80.869%	94,980 / 0.48%

NO_NO18Ac	49,545 / 0.259%	49,542 / 0.259%	96,624 / 0.506%	2,463 / 0.013%
NO_NO19Ac	6,096,060 / 33.4%	6,081,679 / 33.322%	12,134,794 / 66.487%	42,945 / 0.235%
NO_NO20Ac	4,181,578 / 22.258%	4,171,460 / 22.204%	8,309,690 / 44.231%	43,348 / 0.231%
NO_NO21Ac	5,424,735 / 34.981%	5,411,281 / 34.895%	10,791,499 / 69.589%	44,517 / 0.287%
NO_NO22Ac	4,982,627 / 40.965%	4,973,509 / 40.89%	9,925,219 / 81.602%	30,917 / 0.254%
NO_NO23Ac	71,818 / 0.417%	71,465 / 0.415%	133,198 / 0.774%	10,085 / 0.059%
NO_NO24Ac	28,742 / 0.189%	28,597 / 0.188%	55,225 / 0.363%	2,114 / 0.014%
NO_NO25Ac	6,033,291 / 34.529%	6,013,583 / 34.416%	11,989,627 / 68.617%	57,247 / 0.328%
NO_NO27Ac	6,509,376 / 43.814%	6,494,957 / 43.717%	12,954,271 / 87.194%	50,062 / 0.337%
NO_NO28Ac	8,653,933 / 49.113%	8,628,635 / 48.97%	17,212,670 / 97.686%	69,898 / 0.397%
NO_NO29Ac	7,446,322 / 43.155%	7,421,315 / 43.01%	14,794,118 / 85.739%	73,519 / 0.426%
NO_NO30Ac	5,434,216 / 44.05%	5,419,679 / 43.932%	10,811,058 / 87.635%	42,837 / 0.347%
NO_NO31Ac	5,006,204 / 36.527%	4,994,402 / 36.441%	9,962,150 / 72.687%	38,456 / 0.281%
NO_NO32Ac	4,512,267 / 22.454%	4,506,249 / 22.424%	8,981,507 / 44.694%	37,009 / 0.184%
NO_NO33Ac	5,913,968 / 43.097%	5,895,790 / 42.964%	11,761,266 / 85.708%	48,492 / 0.353%
NO_NO34Ac	72,813 / 0.239%	72,470 / 0.237%	142,094 / 0.466%	3,189 / 0.01%
NO_NO35Ac	295,683 / 1.291%	294,409 / 1.285%	570,255 / 2.489%	19,837 / 0.087%
PL_PL2Ac	4,126,488 / 45.235%	4,119,052 / 45.153%	8,219,122 / 90.099%	26,418 / 0.29%
PL_PL3Ac	4,442,701 / 45.891%	4,436,839 / 45.83%	8,859,478 / 91.513%	20,062 / 0.207%
PL_PL1Ac	11,019,970 / 47.253%	10,990,578 / 47.127%	21,925,070 / 94.013%	85,478 / 0.367%
RU_RU3Ac	48,807 / 11.573%	48,797 / 11.571%	97,210 / 23.051%	394 / 0.093%
RU_RU1Ac	5,702,768 / 41.562%	5,694,978 / 41.505%	11,369,618 / 82.861%	28,128 / 0.205%
RU_RU4Ac	1,281,533 / 39.534%	1,279,121 / 39.46%	2,555,661 / 78.84%	4,993 / 0.154%
RU_RU2Ac	6,625,876 / 42.612%	6,613,248 / 42.531%	13,195,187 / 84.86%	43,937 / 0.283%
RU_RU5Ac	3,700,967 / 43.025%	3,695,444 / 42.961%	7,365,107 / 85.623%	31,304 / 0.364%
RU_RU6Ac	2,574,490 / 37.307%	2,569,722 / 37.238%	5,122,641 / 74.232%	21,571 / 0.313%
SK_SK1Ac	12,225,437 / 46.664%	12,156,329 / 46.4%	24,237,783 / 92.515%	143,983 / 0.55%
SK_SK2Ac	8,585,709 / 39.106%	8,474,112 / 38.598%	16,891,219 / 76.937%	168,602 / 0.768%
SK_SK3Ac	6,069,737 / 33.703%	6,025,709 / 33.459%	12,010,709 / 66.691%	84,737 / 0.471%
US_US1Ac	1,621,106 / 36.133%	1,618,233 / 36.069%	3,224,241 / 71.865%	15,098 / 0.337%
SK_SK4Ac	8,195,487 / 38.934%	8,168,295 / 38.805%	16,280,230 / 77.341%	83,552 / 0.397%
SK_SK5Ac	7,959,695 / 44.613%	7,913,899 / 44.356%	15,786,103 / 88.479%	87,491 / 0.49%

ACTG Content

Sample	A's	C's	T's	G's	N's	GC (%)
AT_AT2Ac	217,259,653 / 24.351%	229,281,661 / 25.698%	217,823,742 / 24.414%	227,843,303 / 25.537%	33,980 / 0.004%	51.24
AT_AT3Ac	282,053,671 / 23.708%	313,646,059 / 26.364%	282,637,193 / 23.758%	311,336,622 / 26.17%	45,677 / 0.004%	52.53
AT_AT1Ac	213,232,339 / 26.011%	195,609,083 / 23.862%	215,191,275 / 26.25%	195,732,318 / 23.877%	32,094 / 0.004%	47.74
CZ_CZ1Ac	111,083,979 / 24.611%	115,167,923 / 25.516%	110,767,940 / 24.541%	114,338,503 / 25.332%	15,498 / 0.003%	50.85
CZ_CZ4Ac	11,828,504 / 24.448%	12,381,085 / 25.591%	11,868,729 / 24.532%	12,303,092 / 25.429%	1,794 / 0.004%	51.02
CZ_CZ2Ac	285,777,000 / 25.486%	274,166,711 / 24.451%	287,551,411 / 25.644%	273,810,201 / 24.419%	43,470 / 0.004%	48.87
CZ_CZ3Ac	580,824,886 / 24.642%	598,207,278 / 25.379%	582,389,664 / 24.708%	595,627,635 / 25.27%	92,978 / 0.004%	50.65
CZ_CZ5Ac	537,036,338 / 26.062%	490,902,854 / 23.824%	541,970,565 / 26.302%	490,670,764 / 23.812%	81,086 / 0.004%	47.64
CZ_CZ6Ac	332,882,986 / 24.66%	342,733,751 / 25.39%	333,392,089 / 24.698%	340,858,129 / 25.251%	53,449 / 0.004%	50.64

CZ_CZ7Ac	93,176,379 / 25.064%	92,733,499 / 24.945%	93,395,064 / 25.123%	92,453,549 / 24.869%	15,347 / 0.004%	49.81
CZ_CZ8Ac	226,086,268 / 24.523%	235,237,327 / 25.516%	226,316,020 / 24.548%	234,294,157 / 25.413%	35,220 / 0.004%	50.93
CZ_CZ9Ac	509,957,490 / 24.33%	539,009,254 / 25.716%	510,679,674 / 24.365%	536,334,050 / 25.589%	79,364 / 0.004%	51.31
CZ_CZ14Ac	388,069,015 / 24.928%	390,450,258 / 25.081%	389,665,039 / 25.031%	388,566,078 / 24.96%	59,476 / 0.004%	50.04
CZ_CZ16Ac	299,138,633 / 25.027%	298,626,459 / 24.984%	300,139,171 / 25.11%	297,373,802 / 24.879%	47,083 / 0.004%	49.86
CZ_CZ15Ac	611,645,990 / 25.404%	590,708,463 / 24.535%	615,953,002 / 25.583%	589,356,607 / 24.478%	89,953 / 0.004%	49.01
CZ_CZ18Ac	67,025,187 / 24.515%	69,771,488 / 25.519%	67,196,757 / 24.578%	69,412,442 / 25.388%	9,807 / 0.004%	50.91
CZ_CZ17Ac	35,204,803 / 24.477%	36,766,242 / 25.562%	35,246,023 / 24.505%	36,612,448 / 25.455%	4,795 / 0.003%	51.02
CZ_CZ19Ac	359,146,108 / 24.988%	359,523,932 / 25.014%	360,491,039 / 25.082%	358,104,316 / 24.916%	53,956 / 0.004%	49.93
CZ_CZ20Ac	256,868,554 / 24.867%	259,605,401 / 25.132%	257,729,393 / 24.951%	258,750,886 / 25.05%	39,084 / 0.004%	50.18
CZ_CZ21Ac	174,786,525 / 25.321%	170,041,387 / 24.633%	175,690,299 / 25.452%	169,772,543 / 24.594%	27,283 / 0.004%	49.23
CZ_CZ22Ac	120,090,020 / 24.812%	121,909,127 / 25.188%	120,463,138 / 24.889%	121,535,116 / 25.111%	18,956 / 0.004%	50.3
CZ_CZ24Ac	161,445,353 / 24.913%	162,433,794 / 25.065%	162,024,417 / 25.002%	162,135,010 / 25.019%	23,932 / 0.004%	50.08
DE_DE1Ac	7,699,394 / 25.899%	7,306,159 / 24.577%	8,206,657 / 27.606%	6,516,004 / 21.919%	450 / 0.002%	46.5
CZ_CZ23Ac	641,672,725 / 25.223%	629,214,826 / 24.733%	645,020,891 / 25.355%	628,100,449 / 24.689%	99,408 / 0.004%	49.42
DE_DE2Ac	245,874,544 / 24.7%	251,974,703 / 25.313%	246,847,856 / 24.798%	250,750,246 / 25.19%	38,594 / 0.004%	50.5
DE_DE3Ac	334,208,716 / 24.655%	343,519,663 / 25.342%	335,577,106 / 24.756%	342,234,114 / 25.247%	52,169 / 0.004%	50.59
DE_DE4Ac	407,056,513 / 24.89%	410,941,543 / 25.128%	408,635,816 / 24.987%	408,766,136 / 24.995%	63,698 / 0.004%	50.12
DE_DE5Ac	427,976,135 / 25.117%	424,362,124 / 24.904%	429,678,433 / 25.216%	421,946,121 / 24.763%	64,212 / 0.004%	49.67
DE_DE6Ac	413,882,611 / 25.007%	414,198,464 / 25.027%	415,637,888 / 25.114%	411,318,626 / 24.853%	64,670 / 0.004%	49.88
DE_DE7Ac	9,201,129 / 25.258%	9,520,264 / 26.134%	9,598,158 / 26.348%	8,108,686 / 22.259%	913 / 0.003%	48.39
DE_DE8Ac	357,456,128 / 24.76%	364,126,456 / 25.222%	359,276,396 / 24.886%	362,841,712 / 25.133%	56,679 / 0.004%	50.35
DE_DE9Ac	608,850 / 20.473%	889,659 / 29.915%	658,709 / 22.149%	816,715 / 27.462%	18 / 0.001%	57.38
DE_DE10Ac	857,220,926 / 25.435%	826,071,549 / 24.511%	862,107,097 / 25.58%	824,861,675 / 24.475%	128,230 / 0.004%	48.99
DE_DE11Ac	529,959,544 / 24.932%	532,418,872 / 25.047%	531,954,322 / 25.025%	531,326,876 / 24.996%	81,204 / 0.004%	50.04
DE_DE12Ac	9,266,905 / 25.637%	9,164,500 / 25.354%	9,749,056 / 26.971%	7,965,602 / 22.037%	1,009 / 0.003%	47.39
EE_EE1Ac	404,946,439 / 24.724%	413,992,220 / 25.276%	406,250,165 / 24.804%	412,671,777 / 25.196%	63,114 / 0.004%	50.47
EE_EE2Ac	276,202,644 / 24.814%	280,580,576 / 25.207%	277,279,147 / 24.911%	279,029,514 / 25.068%	43,712 / 0.004%	50.28
DE_DE13Ac	400,285,045 / 25.226%	392,651,757 / 24.745%	402,376,081 / 25.357%	391,509,560 / 24.673%	60,127 / 0.004%	49.42
EE_EE3Ac	324,761,896 / 24.334%	342,908,582 / 25.693%	325,531,016 / 24.391%	341,424,277 / 25.582%	52,738 / 0.004%	51.28
EE_EE4Ac	287,701,104 / 25.137%	283,912,658 / 24.806%	289,489,745 / 25.293%	283,449,508 / 24.765%	44,208 / 0.004%	49.57

EE_EE5Ac	245,721,337 / 24.552%	254,803,752 / 25.46%	246,103,290 / 24.59%	254,189,895 / 25.398%	38,571 / 0.004%	50.86
FI_FI1Ac	472,676,375 / 24.789%	480,418,111 / 25.195%	474,341,427 / 24.877%	479,334,099 / 25.139%	73,751 / 0.004%	50.33
FI_FI2Ac	592,041,322 / 25.04%	589,283,415 / 24.924%	594,676,413 / 25.152%	588,360,576 / 24.885%	88,794 / 0.004%	49.81
FI_FI3Ac	313,798,807 / 24.956%	314,737,269 / 25.03%	315,212,341 / 25.068%	313,675,961 / 24.946%	48,647 / 0.004%	49.98
FI_FI4Ac	313,971,283 / 24.904%	316,256,162 / 25.085%	315,241,025 / 25.005%	315,250,829 / 25.006%	40,926 / 0.003%	50.09
FI_FI5Ac	307,399,633 / 24.588%	318,758,322 / 25.497%	307,770,942 / 24.618%	316,257,304 / 25.297%	46,711 / 0.004%	50.79
FI_FI6Ac	196,256,784 / 24.521%	203,994,071 / 25.487%	196,836,537 / 24.593%	203,287,790 / 25.399%	30,560 / 0.004%	50.89
FI_FI7Ac	84,250,211 / 24.375%	88,560,064 / 25.622%	84,478,038 / 24.441%	88,354,439 / 25.562%	14,223 / 0.004%	51.18
FI_FI9Ac	23,505,149 / 25.522%	22,411,230 / 24.335%	23,740,618 / 25.778%	22,438,872 / 24.365%	3,197 / 0.003%	48.7
FI_FI8Ac	174,944,590 / 24.613%	180,526,039 / 25.399%	175,371,067 / 24.673%	179,928,457 / 25.315%	27,577 / 0.004%	50.71
FI_FI11Ac	28,601,147 / 24.298%	30,308,677 / 25.748%	28,653,023 / 24.342%	30,147,841 / 25.612%	4,636 / 0.004%	51.36
FI_FI10Ac	470,087,842 / 24.998%	470,152,550 / 25.001%	471,693,145 / 25.083%	468,603,393 / 24.919%	71,320 / 0.004%	49.92
FI_FI12Ac	169,821,798 / 24.794%	172,707,112 / 25.215%	170,520,189 / 24.896%	171,886,124 / 25.095%	24,763 / 0.004%	50.31
FI_FI13Ac	214,625,487 / 24.548%	222,675,629 / 25.469%	215,241,188 / 24.618%	221,764,956 / 25.365%	34,514 / 0.004%	50.83
FI_FI14Ac	174,958,425 / 24.318%	184,919,221 / 25.703%	175,209,045 / 24.353%	184,367,291 / 25.626%	27,027 / 0.004%	51.33
FI_FI15Ac	256,680,056 / 24.665%	263,609,233 / 25.331%	257,779,388 / 24.771%	262,596,787 / 25.234%	40,648 / 0.004%	50.56
FI_FI16Ac	124,479,349 / 24.268%	132,230,364 / 25.779%	124,497,823 / 24.271%	131,738,153 / 25.683%	20,044 / 0.004%	51.46
FI_FI17Ac	81,140,850 / 24.809%	82,222,404 / 25.14%	81,490,767 / 24.916%	82,204,833 / 25.135%	11,899 / 0.004%	50.27
FI_FI19Ac	174,599,293 / 24.645%	179,644,087 / 25.357%	175,018,157 / 24.704%	179,186,590 / 25.293%	28,727 / 0.004%	50.65
FI_FI21Ac	1,865,930 / 24.823%	1,979,506 / 26.334%	2,009,535 / 26.733%	1,661,987 / 22.11%	217 / 0.003%	48.44
FI_FI20Ac	337,704,784 / 24.752%	344,032,642 / 25.216%	339,189,046 / 24.861%	343,428,669 / 25.172%	51,598 / 0.004%	50.39
FI_FI23Ac	234,400,942 / 24.444%	245,375,659 / 25.589%	235,283,896 / 24.536%	243,860,620 / 25.431%	37,239 / 0.004%	51.02
FI_FI24Ac	3,785,581 / 25.488%	3,687,222 / 24.826%	3,998,460 / 26.921%	3,381,240 / 22.765%	286 / 0.002%	47.59
FI_FI25Ac	284,188,989 / 24.989%	284,006,566 / 24.973%	285,624,687 / 25.116%	283,419,585 / 24.922%	43,710 / 0.004%	49.9
FI_FI22Ac	517,761,570 / 25.812%	482,046,043 / 24.032%	523,634,606 / 26.105%	482,442,139 / 24.051%	77,554 / 0.004%	48.08
FI_FI26Ac	216,648,904 / 25.209%	212,132,198 / 24.683%	218,073,293 / 25.375%	212,557,464 / 24.733%	32,851 / 0.004%	49.42
HR_HR1Ac	8,990 / 25.066%	9,227 / 25.727%	8,577 / 23.915%	9,071 / 25.292%	0	51.02
FI_FI27Ac	290,421,484 / 24.938%	291,707,769 / 25.048%	291,627,483 / 25.041%	290,834,607 / 24.973%	45,089 / 0.004%	50.02
HR_HR4Ac	105,240,459 / 24.034%	113,816,529 / 25.993%	105,312,466 / 24.051%	113,503,952 / 25.922%	17,404 / 0.004%	51.91
GR_GR1Ac	10,048,457 / 25.812%	9,990,907 / 25.664%	10,422,364 / 26.773%	8,467,560 / 21.751%	1,008 / 0.003%	47.42
HR_HR5Ac	244,711,344 / 27.066%	205,898,818 / 22.773%	247,225,291 / 27.344%	206,300,800 / 22.817%	35,235 / 0.004%	45.59

HR_HR6Ac	279,112,911 / 24.327%	294,979,419 / 25.71%	279,677,589 / 24.376%	293,568,532 / 25.587%	44,981 / 0.004%	51.3
HR_HR7Ac	213,440,679 / 24.6%	220,383,679 / 25.4%	213,958,768 / 24.659%	219,875,260 / 25.341%	33,278 / 0.004%	50.74
HR_HR9Ac	273,431,118 / 24.522%	284,005,962 / 25.471%	274,125,738 / 24.584%	283,472,506 / 25.423%	42,348 / 0.004%	50.89
HR_HR12Ac	256,852,260 / 24.686%	263,246,892 / 25.301%	257,938,331 / 24.791%	262,428,558 / 25.222%	37,942 / 0.004%	50.52
LT_LT1Ac	426,846,579 / 24.466%	445,232,803 / 25.52%	428,354,111 / 24.552%	444,228,234 / 25.462%	64,177 / 0.004%	50.98
LT_LT3Ac	399,441,440 / 24.355%	421,149,201 / 25.678%	399,768,231 / 24.375%	419,732,379 / 25.592%	64,944 / 0.004%	51.27
LT_LT2Ac	752,933,473 / 24.755%	767,159,137 / 25.222%	755,403,071 / 24.836%	766,085,583 / 25.187%	111,380 / 0.004%	50.41
LT_LT4Ac	309,376,631 / 24.965%	310,205,149 / 25.032%	310,486,086 / 25.055%	309,155,732 / 24.948%	47,894 / 0.004%	49.98
LT_LT6Ac	322,472,287 / 25.246%	315,446,338 / 24.696%	324,365,866 / 25.394%	315,045,099 / 24.664%	50,938 / 0.004%	49.36
LT_LT5Ac	477,265,266 / 25.019%	475,880,655 / 24.946%	479,494,091 / 25.136%	474,982,656 / 24.899%	75,656 / 0.004%	49.85
NO_NO1Ac	369,480,157 / 24.463%	385,748,958 / 25.54%	370,603,010 / 24.537%	384,541,267 / 25.46%	58,372 / 0.004%	51
NO_NO3Ac	1,461,472 / 23.745%	1,621,591 / 26.347%	1,478,026 / 24.014%	1,593,746 / 25.894%	125 / 0.002%	52.24
NO_NO2Ac	6,420,531 / 25.17%	6,326,261 / 24.8%	6,474,990 / 25.383%	6,287,288 / 24.647%	687 / 0.003%	49.45
NO_NO5Ac	185,637,856 / 24.476%	193,626,899 / 25.529%	186,142,483 / 24.543%	193,040,953 / 25.452%	28,767 / 0.004%	50.98
NO_NO4Ac	72,778,475 / 24.338%	76,851,021 / 25.7%	72,890,972 / 24.376%	76,507,239 / 25.585%	11,644 / 0.004%	51.29
NO_NO7Ac	154,642,847 / 24.795%	156,956,439 / 25.166%	155,413,225 / 24.918%	156,679,279 / 25.121%	23,510 / 0.004%	50.29
NO_NO6Ac	184,115,770 / 24.732%	188,043,853 / 25.259%	184,900,299 / 24.837%	187,397,242 / 25.172%	27,149 / 0.004%	50.43
NO_NO8Ac	316,163,480 / 24.487%	329,321,668 / 25.506%	317,300,510 / 24.575%	328,378,291 / 25.433%	48,342 / 0.004%	50.94
NO_NO9Ac	502,737,939 / 25.783%	469,920,777 / 24.1%	507,393,744 / 26.021%	469,864,259 / 24.097%	72,853 / 0.004%	48.2
NO_NO11Ac	30,522,017 / 24.313%	32,335,057 / 25.757%	30,640,184 / 24.407%	32,042,618 / 25.524%	4,691 / 0.004%	51.28
NO_NO10Ac	440,036,417 / 24.894%	443,608,416 / 25.096%	441,860,198 / 24.997%	442,115,999 / 25.012%	67,436 / 0.004%	50.11
NO_NO12Ac	350,393,791 / 25.005%	349,815,855 / 24.964%	352,290,973 / 25.14%	348,800,192 / 24.891%	53,830 / 0.004%	49.85
NO_NO13Ac	247,833,836 / 24.409%	259,703,560 / 25.578%	248,474,867 / 24.472%	259,319,875 / 25.54%	40,037 / 0.004%	51.12
NO_NO14Ac	148,429,797 / 25.173%	146,091,655 / 24.776%	149,040,281 / 25.277%	146,077,264 / 24.774%	24,296 / 0.004%	49.55
NO_NO15Ac	331,095,473 / 24.884%	334,201,236 / 25.118%	332,334,559 / 24.977%	332,904,790 / 25.02%	51,955 / 0.004%	50.14
NO_NO16Ac	449,548,106 / 24.302%	475,727,244 / 25.717%	450,370,522 / 24.347%	474,185,464 / 25.634%	71,290 / 0.004%	51.35
NO_NO17Ac	579,818,850 / 24.487%	604,713,044 / 25.539%	580,707,484 / 24.525%	602,592,686 / 25.449%	90,164 / 0.004%	50.99
NO_NO18Ac	758,269 / 22.242%	947,299 / 27.786%	775,098 / 22.735%	928,547 / 27.236%	97 / 0.003%	55.02
NO_NO19Ac	435,453,889 / 24.451%	455,797,192 / 25.593%	436,244,127 / 24.495%	453,453,475 / 25.461%	70,557 / 0.004%	51.05
NO_NO20Ac	297,992,732 / 24.385%	313,501,995 / 25.654%	298,709,946 / 24.444%	311,825,562 / 25.517%	47,745 / 0.004%	51.17
NO_NO21Ac	390,949,877 / 24.609%	403,932,893 / 25.426%	391,915,362 / 24.669%	401,874,462 / 25.296%	62,383 / 0.004%	50.72

NO_NO22Ac	363,241,260 / 24.87%	366,752,514 / 25.11%	364,596,603 / 24.963%	365,981,251 / 25.057%	56,441 / 0.004%	50.17
NO_NO23Ac	2,885,103 / 24.662%	3,050,426 / 26.075%	3,059,265 / 26.15%	2,704,003 / 23.114%	512 / 0.004%	49.19
NO_NO24Ac	995,732 / 23.936%	1,085,509 / 26.094%	1,002,979 / 24.11%	1,075,711 / 25.859%	104 / 0.003%	51.95
NO_NO25Ac	433,523,168 / 24.569%	448,655,525 / 25.426%	434,656,786 / 24.633%	447,694,916 / 25.372%	64,429 / 0.004%	50.8
NO_NO27Ac	472,996,228 / 24.812%	480,507,037 / 25.206%	474,674,991 / 24.9%	478,160,926 / 25.083%	73,143 / 0.004%	50.29
NO_NO28Ac	624,647,229 / 24.548%	648,110,737 / 25.47%	625,420,903 / 24.578%	646,456,743 / 25.405%	98,421 / 0.004%	50.87
NO_NO29Ac	546,139,493 / 24.967%	546,729,670 / 24.994%	548,602,800 / 25.08%	545,935,692 / 24.958%	84,446 / 0.004%	49.95
NO_NO30Ac	400,301,162 / 25.115%	396,119,013 / 24.853%	402,283,255 / 25.239%	395,173,263 / 24.793%	63,089 / 0.004%	49.65
NO_NO31Ac	367,331,042 / 25.046%	365,676,432 / 24.934%	368,951,618 / 25.157%	364,638,324 / 24.863%	57,747 / 0.004%	49.8
NO_NO32Ac	322,291,941 / 24.686%	330,589,305 / 25.321%	323,462,230 / 24.776%	329,228,082 / 25.217%	46,615 / 0.004%	50.54
NO_NO33Ac	427,525,750 / 24.661%	439,350,909 / 25.343%	428,617,741 / 24.724%	438,093,194 / 25.271%	67,504 / 0.004%	50.61
NO_NO34Ac	2,583,352 / 24.624%	2,662,496 / 25.378%	2,609,236 / 24.871%	2,636,097 / 25.127%	235 / 0.002%	50.51
NO_NO35Ac	18,650,313 / 25.366%	18,185,120 / 24.733%	18,790,059 / 25.556%	17,899,431 / 24.345%	2,161 / 0.003%	49.08
PL_PL2Ac	298,821,851 / 24.924%	300,726,174 / 25.083%	299,973,421 / 25.02%	299,390,934 / 24.972%	47,983 / 0.004%	50.06
PL_PL3Ac	306,098,228 / 24.523%	318,352,141 / 25.505%	306,696,539 / 24.571%	317,040,394 / 25.4%	46,736 / 0.004%	50.91
PL_PL1Ac	796,841,830 / 24.6%	823,758,352 / 25.431%	798,234,609 / 24.643%	820,325,649 / 25.325%	127,292 / 0.004%	50.76
RU_RU3Ac	2,729,825 / 24.071%	2,935,986 / 25.889%	2,750,275 / 24.251%	2,924,752 / 25.79%	434 / 0.004%	51.68
RU_RU1Ac	369,480,650 / 24.464%	386,134,428 / 25.567%	370,129,267 / 24.507%	384,541,693 / 25.462%	49,700 / 0.003%	51.03
RU_RU4Ac	86,985,818 / 24.541%	90,306,404 / 25.477%	87,287,321 / 24.626%	89,877,886 / 25.356%	13,443 / 0.004%	50.83
RU_RU2Ac	484,829,106 / 24.999%	484,176,635 / 24.966%	487,022,103 / 25.112%	483,335,556 / 24.922%	77,067 / 0.004%	49.89
RU_RU5Ac	255,726,433 / 24.339%	269,993,085 / 25.696%	256,384,892 / 24.401%	268,600,205 / 25.564%	37,245 / 0.004%	51.26
RU_RU6Ac	176,675,414 / 24.42%	184,984,444 / 25.569%	177,385,676 / 24.518%	184,432,256 / 25.492%	27,976 / 0.004%	51.06
SK_SK1Ac	883,663,125 / 24.606%	913,676,824 / 25.442%	884,998,190 / 24.643%	908,918,415 / 25.309%	136,024 / 0.004%	50.75
SK_SK2Ac	624,251,996 / 24.912%	629,106,329 / 25.106%	626,212,818 / 24.99%	626,270,167 / 24.992%	95,361 / 0.004%	50.1
SK_SK3Ac	442,075,838 / 24.88%	445,788,998 / 25.089%	443,566,064 / 24.964%	445,390,913 / 25.067%	68,239 / 0.004%	50.16
US_US1Ac	112,620,469 / 24.808%	114,301,167 / 25.178%	113,305,808 / 24.959%	113,737,273 / 25.054%	16,698 / 0.004%	50.23
SK_SK4Ac	602,050,919 / 24.986%	602,577,351 / 25.008%	604,644,318 / 25.093%	600,304,056 / 24.913%	90,262 / 0.004%	49.92
SK_SK5Ac	587,916,250 / 25.158%	580,119,039 / 24.824%	590,708,781 / 25.277%	578,190,616 / 24.741%	90,778 / 0.004%	49.57

Coverage

Sample	Mean	Standard Deviation
AT_AT2Ac	27.044X	48.896X
AT_AT3Ac	36.061X	79.756X

AT_AT1Ac	24.848X	104.09X
CZ_CZ1Ac	13.681X	30.066X
CZ_CZ4Ac	1.466X	2.804X
CZ_CZ2Ac	33.987X	100.856X
CZ_CZ3Ac	71.445X	111.224X
CZ_CZ5Ac	62.459X	278.502X
CZ_CZ6Ac	40.917X	70.045X
CZ_CZ7Ac	11.268X	29.475X
CZ_CZ8Ac	27.945X	34.073X
CZ_CZ9Ac	63.532X	85.409X
CZ_CZ14Ac	47.188X	101.051X
CZ_CZ16Ac	36.231X	79.3X
CZ_CZ15Ac	72.978X	219.469X
CZ_CZ18Ac	8.287X	17.242X
CZ_CZ17Ac	4.36X	9.179X
CZ_CZ19Ac	43.566X	96.617X
CZ_CZ20Ac	31.311X	62.446X
CZ_CZ21Ac	20.924X	57.32X
CZ_CZ22Ac	14.67X	30.47X
CZ_CZ24Ac	19.643X	39.211X
DE_DE1Ac	0.901X	34.634X
CZ_CZ23Ac	77.112X	192.062X
DE_DE2Ac	30.173X	47.655X
DE_DE3Ac	41.085X	67.177X
DE_DE4Ac	49.571X	98.788X
DE_DE5Ac	51.65X	120.168X
DE_DE6Ac	50.164X	111.577X
DE_DE7Ac	1.105X	50.466X
DE_DE8Ac	43.759X	80.553X
DE_DE9Ac	0.09X	2.67X
DE_DE10Ac	102.157X	317.231X
DE_DE11Ac	64.431X	131.59X
DE_DE12Ac	1.096X	45.644X
EE_EE1Ac	49.646X	82.281X
EE_EE2Ac	33.74X	69.274X
DE_DE13Ac	48.098X	119.397X
EE_EE3Ac	40.454X	56.352X
EE_EE4Ac	34.693X	94.664X
EE_EE5Ac	30.336X	43.619X
FI_FI1Ac	57.797X	104.894X
FI_FI2Ac	71.669X	155.391X
FI_FI3Ac	38.114X	81.269X
FI_FI4Ac	38.214X	78.247X
FI_FI5Ac	37.896X	80.217X
FI_FI6Ac	24.261X	39.646X
FI_FI7Ac	10.477X	17.455X
FI_FI9Ac	2.792X	20.47X
FI_FI8Ac	21.544X	36.851X
FI_FI11Ac	3.568X	6.031X
FI_FI10Ac	57.003X	185.107X
FI_FI12Ac	20.761X	43.732X
FI_FI13Ac	26.501X	40.08X

FI_FI14Ac	21.808X	30.091X
FI_FI15Ac	31.544X	57.616X
FI_FI16Ac	15.548X	21.586X
FI_FI17Ac	9.913X	24.205X
FI_FI19Ac	21.474X	37.333X
FI_FI21Ac	0.228X	13.94X
FI_FI20Ac	41.356X	100.797X
FI_FI23Ac	29.065X	50.222X
FI_FI24Ac	0.45X	15.704X
FI_FI25Ac	34.472X	94.409X
FI_FI22Ac	60.801X	260.792X
FI_FI26Ac	26.049X	87.183X
HR_HR1Ac	0.001X	0.037X
FI_FI27Ac	35.302X	93.578X
HR_HR4Ac	13.272X	19.648X
GR_GR1Ac	1.18X	56.091X
HR_HR5Ac	27.406X	169.381X
HR_HR6Ac	34.778X	50.759X
HR_HR7Ac	26.3X	38.934X
HR_HR9Ac	33.798X	52.296X
HR_HR12Ac	31.539X	53.962X
LT_LT1Ac	52.883X	72.121X
LT_LT3Ac	49.714X	66.324X
LT_LT2Ac	92.193X	166.314X
LT_LT4Ac	37.563X	78.923X
LT_LT6Ac	38.719X	97.317X
LT_LT5Ac	57.824X	122.261X
NO_NO1Ac	45.782X	59.831X
NO_NO3Ac	0.187X	1.027X
NO_NO2Ac	0.773X	2.76X
NO_NO5Ac	22.99X	34.618X
NO_NO4Ac	9.064X	14.049X
NO_NO7Ac	18.906X	40.408X
NO_NO6Ac	22.566X	42.14X
NO_NO8Ac	39.136X	56.179X
NO_NO9Ac	59.107X	221.445X
NO_NO11Ac	3.805X	9.935X
NO_NO10Ac	53.579X	100.937X
NO_NO12Ac	42.475X	93.447X
NO_NO13Ac	30.776X	42.021X
NO_NO14Ac	17.873X	52.636X
NO_NO15Ac	40.329X	87.28X
NO_NO16Ac	56.071X	66.279X
NO_NO17Ac	71.772X	101.424X
NO_NO18Ac	0.103X	1.655X
NO_NO19Ac	53.984X	100.36X
NO_NO20Ac	37.041X	53.505X
NO_NO21Ac	48.154X	79.203X
NO_NO22Ac	44.27X	95.43X
NO_NO23Ac	0.355X	12.131X
NO_NO24Ac	0.126X	0.964X
NO_NO25Ac	53.483X	72.962X

NO_NO27Ac	57.786X	109.928X
NO_NO28Ac	77.131X	116.475X
NO_NO29Ac	66.302X	138.162X
NO_NO30Ac	48.313X	122.514X
NO_NO31Ac	44.454X	100.723X
NO_NO32Ac	39.575X	68.773X
NO_NO33Ac	52.549X	83.303X
NO_NO34Ac	0.318X	1.772X
NO_NO35Ac	2.229X	11.457X
PL_PL2Ac	36.341X	73.35X
PL_PL3Ac	37.836X	54.822X
PL_PL1Ac	98.183X	171.409X
RU_RU3Ac	0.344X	1.701X
RU_RU1Ac	45.778X	60.982X
RU_RU4Ac	10.744X	25.247X
RU_RU2Ac	58.785X	120.775X
RU_RU5Ac	31.869X	61.993X
RU_RU6Ac	21.944X	40.02X
SK_SK1Ac	108.859X	202.457X
SK_SK2Ac	75.957X	161.887X
SK_SK3Ac	53.859X	110.91X
US_US1Ac	13.777X	29.623X
SK_SK4Ac	73.038X	164.893X
SK_SK5Ac	70.837X	180.888X

Mappping Quality

Sample	Mean Mapping Quality
AT_AT2Ac	51.446
AT_AT3Ac	51.335
AT_AT1Ac	51.846
CZ_CZ1Ac	51.265
CZ_CZ4Ac	51.291
CZ_CZ2Ac	51.089
CZ_CZ3Ac	52.25
CZ_CZ5Ac	52.025
CZ_CZ6Ac	52.09
CZ_CZ7Ac	51.577
CZ_CZ8Ac	51.764
CZ_CZ9Ac	51.443
CZ_CZ14Ac	51.995
CZ_CZ16Ac	51.723
CZ_CZ15Ac	51.456
CZ_CZ18Ac	51.04
CZ_CZ17Ac	51.411
CZ_CZ19Ac	51.557
CZ_CZ20Ac	51.791
CZ_CZ21Ac	51.241
CZ_CZ22Ac	51.466
CZ_CZ24Ac	51.788
DE_DE1Ac	32.394
CZ_CZ23Ac	51.22
DE_DE2Ac	51.509

DE_DE3Ac	51.847
DE_DE4Ac	52.002
DE_DE5Ac	51.786
DE_DE6Ac	51.937
DE_DE7Ac	33.472
DE_DE8Ac	51.902
DE_DE9Ac	32.885
DE_DE10Ac	51.851
DE_DE11Ac	51.059
DE_DE12Ac	32.357
EE_EE1Ac	51.46
EE_EE2Ac	51.382
DE_DE13Ac	51.323
EE_EE3Ac	51.464
EE_EE4Ac	51.481
EE_EE5Ac	51.627
FI_FI1Ac	51.033
FI_FI2Ac	51.727
FI_FI3Ac	51.938
FI_FI4Ac	51.666
FI_FI5Ac	51.843
FI_FI6Ac	51.482
FI_FI7Ac	50.428
FI_FI9Ac	36.984
FI_FI8Ac	51.616
FI_FI11Ac	51.553
FI_FI10Ac	50.654
FI_FI12Ac	51.636
FI_FI13Ac	51.485
FI_FI14Ac	51.25
FI_FI15Ac	50.938
FI_FI16Ac	51.355
FI_FI17Ac	51.531
FI_FI19Ac	51.31
FI_FI21Ac	30.079
FI_FI20Ac	50.517
FI_FI23Ac	51.13
FI_FI24Ac	32.419
FI_FI25Ac	50.179
FI_FI22Ac	51.335
FI_FI26Ac	50.71
HR_HR1Ac	4.056
FI_FI27Ac	50.902
HR_HR4Ac	51.675
GR_GR1Ac	36.933
HR_HR5Ac	51.706
HR_HR6Ac	52.188
HR_HR7Ac	52.386
HR_HR9Ac	51.597
HR_HR12Ac	51.709
LT_LT1Ac	51.321
LT_LT3Ac	51.409

LT_LT2Ac	51.94
LT_LT4Ac	51.656
LT_LT6Ac	51.59
LT_LT5Ac	51.322
NO_NO1Ac	51.99
NO_NO3Ac	45.417
NO_NO2Ac	50.045
NO_NO5Ac	52.061
NO_NO4Ac	51.444
NO_NO7Ac	51.933
NO_NO6Ac	51.855
NO_NO8Ac	51.721
NO_NO9Ac	51.166
NO_NO11Ac	51.2
NO_NO10Ac	51.434
NO_NO12Ac	51.229
NO_NO13Ac	51.867
NO_NO14Ac	52.151
NO_NO15Ac	51.763
NO_NO16Ac	51.372
NO_NO17Ac	51.599
NO_NO18Ac	36.66
NO_NO19Ac	51.41
NO_NO20Ac	51.719
NO_NO21Ac	51.648
NO_NO22Ac	51.572
NO_NO23Ac	45.826
NO_NO24Ac	43.105
NO_NO25Ac	51.333
NO_NO27Ac	51.703
NO_NO28Ac	51.241
NO_NO29Ac	51.306
NO_NO30Ac	51.669
NO_NO31Ac	51.258
NO_NO32Ac	51.756
NO_NO33Ac	51.659
NO_NO34Ac	46.406
NO_NO35Ac	50.684
PL_PL2Ac	51.502
PL_PL3Ac	51.435
PL_PL1Ac	51.653
RU_RU3Ac	45.087
RU_RU1Ac	51.138
RU_RU4Ac	51.781
RU_RU2Ac	51.558
RU_RU5Ac	50.896
RU_RU6Ac	50.045
SK_SK1Ac	50.911
SK_SK2Ac	51.098
SK_SK3Ac	51.472
US_US1Ac	49.76
SK_SK4Ac	51.361

SK_SK5Ac	51.099

Insert Size

Sample	Mean	Standard Deviation	Median
AT_AT2Ac	375.786	440.333	345
AT_AT3Ac	375.81	644.676	333
AT_AT1Ac	391.955	460.918	366
CZ_CZ1Ac	388.871	469.766	370
CZ_CZ4Ac	420.324	455.532	389
CZ_CZ2Ac	458.408	544.844	446
CZ_CZ3Ac	434.854	431.178	413
CZ_CZ5Ac	427.799	524.208	416
CZ_CZ6Ac	427.211	632.685	412
CZ_CZ7Ac	517.956	520.854	511
CZ_CZ8Ac	451.228	481.491	437
CZ_CZ9Ac	452.677	435.366	418
CZ_CZ14Ac	360.351	446.583	328
CZ_CZ16Ac	378.55	452.321	347
CZ_CZ15Ac	392.119	454.296	352
CZ_CZ18Ac	146.852	414.572	136
CZ_CZ17Ac	315.827	562.991	306
CZ_CZ19Ac	456.358	562.447	409
CZ_CZ20Ac	390.874	538.838	346
CZ_CZ21Ac	441.041	607.375	385
CZ_CZ22Ac	421.673	520.075	372
CZ_CZ24Ac	329.372	379.196	317
DE_DE1Ac	231.258	644.163	226
CZ_CZ23Ac	435.887	570.503	386
DE_DE2Ac	300.245	390.381	281
DE_DE3Ac	308.403	555.878	288
DE_DE4Ac	273.492	414.263	259
DE_DE5Ac	348.625	450.315	325
DE_DE6Ac	353.268	415.625	335
DE_DE7Ac	202.694	395.536	195
DE_DE8Ac	382.815	512.893	351
DE_DE9Ac	46.803	94.941	19
DE_DE10Ac	385.725	516.342	353
DE_DE11Ac	373.864	456.536	346
DE_DE12Ac	263.592	411.153	266
EE_EE1Ac	407.84	545.269	376
EE_EE2Ac	247.144	327.247	232
DE_DE13Ac	410.149	500.539	385
EE_EE3Ac	382.921	435.603	354
EE_EE4Ac	388.284	409.922	361
EE_EE5Ac	387.944	408.705	364
FI_FI1Ac	430.748	475.464	401
FI_FI2Ac	451.14	501.876	405
FI_FI3Ac	450.903	556.818	406
FI_FI4Ac	360.346	415.103	322
FI_FI5Ac	252.064	340.96	231
FI_FI6Ac	288.866	383.182	255
FI_FI7Ac	181.972	269.895	165

FI_FI9Ac	262.049	327.933	241
FI_FI8Ac	295.665	355.083	267
FI_FI11Ac	227.299	416.993	211
FI_FI10Ac	368.146	511.149	319
FI_FI12Ac	274.193	372.351	244
FI_FI13Ac	303.258	466.301	268
FI_FI14Ac	266.37	441.772	233
FI_FI15Ac	275.904	414.694	244
FI_FI16Ac	234.491	314.357	214
FI_FI17Ac	228.921	409.074	192
FI_FI19Ac	322.153	463.984	276
FI_FI21Ac	84.727	120.744	20
FI_FI20Ac	353.924	443.952	304
FI_FI23Ac	288.147	377.351	253
FI_FI24Ac	229.746	2,097.982	46
FI_FI25Ac	337.212	456.248	293
FI_FI22Ac	332.239	428.326	287
FI_FI26Ac	353.628	486.73	306
HR_HR1Ac	324.153	146.334	311
FI_FI27Ac	360.031	467.842	308
HR_HR4Ac	185.152	313.303	173
GR_GR1Ac	227.926	1,393	174
HR_HR5Ac	262.547	405.557	249
HR_HR6Ac	292.074	412.19	268
HR_HR7Ac	327.754	420.118	297
HR_HR9Ac	303.652	457.768	275
HR_HR12Ac	343.432	428.498	319
LT_LT1Ac	364.689	494.543	338
LT_LT3Ac	394.54	463.35	356
LT_LT2Ac	382.779	550.13	341
LT_LT4Ac	395.129	459.42	358
LT_LT6Ac	377.234	423.202	348
LT_LT5Ac	413.115	504.629	377
NO_NO1Ac	362.486	464.382	329
NO_NO3Ac	172.816	213.252	21
NO_NO2Ac	268.115	408.242	289
NO_NO5Ac	325.131	629.297	303
NO_NO4Ac	305.566	343.241	287
NO_NO7Ac	310.827	363.099	296
NO_NO6Ac	269.438	456.762	254
NO_NO8Ac	371.263	584.974	334
NO_NO9Ac	417.253	567.114	372
NO_NO11Ac	288.824	401.095	276
NO_NO10Ac	427.458	608.048	388
NO_NO12Ac	374.223	550.667	338
NO_NO13Ac	330.123	610.964	304
NO_NO14Ac	274.398	407.818	250
NO_NO15Ac	393.132	531.052	370
NO_NO16Ac	400.421	678.782	361
NO_NO17Ac	458.676	571.826	405
NO_NO18Ac	62.724	175.625	19
NO_NO19Ac	354.451	396.111	313

NO_NO20Ac	422.757	502.877	388
NO_NO21Ac	381.92	505.461	344
NO_NO22Ac	340.932	509.075	312
NO_NO23Ac	236.287	502.866	237
NO_NO24Ac	187.013	273.773	20
NO_NO25Ac	398.12	575.656	361
NO_NO27Ac	378.884	572.229	342
NO_NO28Ac	438.673	544.768	395
NO_NO29Ac	456.64	565.805	411
NO_NO30Ac	399.351	483.233	360
NO_NO31Ac	377.998	474.975	338
NO_NO32Ac	331.387	563.603	296
NO_NO33Ac	400.556	553.398	356
NO_NO34Ac	179.121	439.875	20
NO_NO35Ac	326.752	448.445	323
PL_PL2Ac	305.035	556.239	286
PL_PL3Ac	213.444	401.215	201
PL_PL1Ac	410.856	495.095	367
RU_RU3Ac	156.416	150.594	146
RU_RU1Ac	174.176	548.519	167
RU_RU4Ac	227.093	299.402	210
RU_RU2Ac	346.96	471.005	324
RU_RU5Ac	272.381	382.646	252
RU_RU6Ac	272.098	419.497	247
SK_SK1Ac	505.413	554.829	447
SK_SK2Ac	528.608	634.337	461
SK_SK3Ac	477.786	589.404	415
US_US1Ac	272.422	413.295	254
SK_SK4Ac	481.388	632.391	425
SK_SK5Ac	481.196	564.059	425

Mismatches and Indels

Sample	General Error Rate	Mismatches	Insertions	Mapped Reads with Insertion (%)	Deletions	Mapped Reds with Deletion (%)	Homopolymer Indels (%)
AT_AT2Ac	0.006	5,107,183	133,571	2.08	164,973	2.53	42.05
AT_AT3Ac	0.006	6,994,216	175,842	2.03	216,995	2.48	44.23
AT_AT1Ac	0.006	4,476,459	138,831	2.31	181,034	2.85	51.31
CZ_CZ1Ac	0.006	2,616,065	64,837	1.95	82,132	2.43	41.59
CZ_CZ4Ac	0.007	295,953	7,127	2	9,031	2.51	42.47
CZ_CZ2Ac	0.006	6,530,364	180,128	2.21	234,213	2.73	49.38
CZ_CZ3Ac	0.006	13,516,115	347,121	2.05	431,525	2.51	43.1
CZ_CZ5Ac	0.006	11,582,691	345,719	2.3	480,267	3.04	53.92
CZ_CZ6Ac	0.006	7,804,838	194,558	2.01	252,371	2.55	43.64
CZ_CZ7Ac	0.007	2,554,577	58,956	2.15	71,089	2.58	45.35
CZ_CZ8Ac	0.006	5,342,851	132,041	1.99	165,345	2.46	42.33
CZ_CZ9Ac	0.007	13,528,733	295,389	1.96	373,396	2.45	40.85
CZ_CZ14Ac	0.006	8,848,672	251,689	2.24	310,391	2.68	45.87
CZ_CZ16Ac	0.006	6,885,578	195,928	2.25	241,104	2.71	45.21
CZ_CZ15Ac	0.007	14,392,524	379,350	2.17	490,431	2.68	47.44
CZ_CZ18Ac	0.006	1,525,880	40,576	1.61	49,704	1.97	42.37

CZ_CZ17Ac	0.006	802,401	22,126	1.95	26,778	2.35	41.85
CZ_CZ19Ac	0.007	9,129,830	230,401	2.21	286,905	2.67	45.7
CZ_CZ20Ac	0.006	6,003,205	163,793	2.18	207,837	2.68	43.99
CZ_CZ21Ac	0.007	4,379,392	115,949	2.29	145,753	2.78	46.52
CZ_CZ22Ac	0.007	3,101,257	76,561	2.14	94,076	2.57	44.69
CZ_CZ24Ac	0.006	3,675,187	100,954	2.14	125,894	2.6	44.14
DE_DE1Ac	0.059	1,716,416	17,027	3.85	18,730	4.63	30.98
CZ_CZ23Ac	0.006	15,176,564	398,085	2.17	515,622	2.69	46.61
DE_DE2Ac	0.006	5,451,140	155,517	2.06	187,694	2.45	42.97
DE_DE3Ac	0.006	6,820,933	188,843	1.93	240,114	2.4	43.03
DE_DE4Ac	0.006	8,608,577	246,492	2.07	312,555	2.53	45.52
DE_DE5Ac	0.006	9,213,014	268,542	2.19	345,059	2.7	46.99
DE_DE6Ac	0.006	8,826,968	235,669	1.97	310,316	2.52	45.14
DE_DE7Ac	0.045	1,608,009	15,993	3.02	23,241	4.51	31.44
DE_DE8Ac	0.006	7,848,578	214,793	2.07	268,529	2.51	44.4
DE_DE9Ac	0.022	62,661	940	0.83	1,408	1.1	23.55
DE_DE10Ac	0.006	19,817,993	535,455	2.19	706,359	2.8	48.8
DE_DE11Ac	0.006	11,715,986	340,522	2.22	418,656	2.65	45.15
DE_DE12Ac	0.053	1,899,415	17,776	3.52	22,803	4.8	29.87
EE_EE1Ac	0.006	9,546,767	247,692	2.1	312,071	2.58	42.63
EE_EE2Ac	0.006	5,982,585	176,146	2.09	223,223	2.55	44.68
DE_DE13Ac	0.006	9,018,216	257,631	2.23	317,237	2.66	45.75
EE_EE3Ac	0.006	7,874,866	199,920	2.08	246,250	2.53	40.94
EE_EE4Ac	0.006	6,311,961	199,038	2.38	231,281	2.71	47.35
EE_EE5Ac	0.006	5,863,366	156,260	2.15	182,692	2.5	41.59
FI_FI1Ac	0.006	11,273,494	310,567	2.25	374,121	2.65	44.54
FI_FI2Ac	0.007	14,781,803	384,862	2.25	480,137	2.71	46.06
FI_FI3Ac	0.007	7,794,278	200,607	2.19	250,876	2.67	44.51
FI_FI4Ac	0.009	10,656,958	198,938	2.2	251,008	2.68	44.54
FI_FI5Ac	0.006	6,940,852	199,631	2.17	245,217	2.62	41.48
FI_FI6Ac	0.006	4,580,540	123,607	2.05	156,650	2.57	42.52
FI_FI7Ac	0.006	1,949,972	53,140	1.85	64,181	2.2	42.58
FI_FI9Ac	0.006	487,751	14,904	2.01	18,495	2.58	48.34
FI_FI8Ac	0.006	3,949,598	111,720	2.09	136,305	2.51	44.43
FI_FI11Ac	0.006	619,656	17,713	1.81	21,127	2.14	41.63
FI_FI10Ac	0.006	10,911,523	329,831	2.37	375,122	2.69	46.53
FI_FI12Ac	0.006	3,705,989	108,004	2.06	135,098	2.53	44.02
FI_FI13Ac	0.006	4,959,389	136,171	2.08	165,674	2.47	43.35
FI_FI14Ac	0.007	4,396,122	111,879	2.04	134,238	2.42	40.81
FI_FI15Ac	0.006	5,893,999	161,429	2.08	199,163	2.5	43.4
FI_FI16Ac	0.006	2,833,803	75,974	1.92	94,555	2.36	40.83
FI_FI17Ac	0.006	1,709,554	51,702	1.97	63,470	2.38	45.87
FI_FI19Ac	0.006	4,047,668	109,986	2.07	136,571	2.54	43.45
FI_FI21Ac	0.046	336,099	3,867	1.66	7,762	3.31	53.59
FI_FI20Ac	0.007	8,294,274	224,746	2.22	267,581	2.6	45
FI_FI23Ac	0.006	5,322,446	141,530	1.98	177,616	2.43	42.23
FI_FI24Ac	0.052	763,580	6,179	2.27	9,060	3.55	25.31
FI_FI25Ac	0.006	6,335,224	184,153	2.19	231,585	2.72	46.48
FI_FI22Ac	0.006	11,306,080	337,022	2.24	435,070	2.75	51.12
FI_FI26Ac	0.006	4,689,001	150,870	2.38	176,593	2.67	49.31
HR_HR1Ac	0.006	196	6	1.98	3	1.19	33.33
FI_FI27Ac	0.007	6,943,692	212,444	2.45	236,729	2.68	47.03

HR_HR4Ac	0.006	2,279,364	68,076	1.95	78,540	2.23	41.92
GR_GR1Ac	0.041	1,561,443	16,166	3.17	18,779	3.82	35.03
HR_HR5Ac	0.005	4,315,989	169,986	2.51	235,326	3.06	59.37
HR_HR6Ac	0.006	6,381,725	181,569	2.14	217,902	2.52	40.9
HR_HR7Ac	0.006	5,006,816	133,521	2.1	166,144	2.55	44.4
HR_HR9Ac	0.006	6,204,311	166,543	2.02	208,826	2.49	42.49
HR_HR12Ac	0.006	5,825,337	165,471	2.17	203,662	2.6	43.72
LT_LT1Ac	0.007	10,681,248	258,045	2.05	324,427	2.54	41.41
LT_LT3Ac	0.007	9,834,715	248,613	2.1	301,792	2.52	41.27
LT_LT2Ac	0.007	20,914,082	472,556	2.15	587,331	2.61	43.52
LT_LT4Ac	0.006	7,265,660	199,801	2.22	248,864	2.7	44.74
LT_LT6Ac	0.006	7,147,095	214,197	2.32	268,983	2.8	47.92
LT_LT5Ac	0.006	10,964,458	305,978	2.21	388,366	2.73	45.43
NO_NO1Ac	0.006	8,831,901	229,883	2.1	280,509	2.52	40.62
NO_NO3Ac	0.009	49,088	937	1.07	1,290	1.41	40.46
NO_NO2Ac	0.006	148,055	4,122	1.7	5,421	2.13	48.78
NO_NO5Ac	0.006	4,166,071	122,218	2.22	142,336	2.56	41.34
NO_NO4Ac	0.006	1,709,124	45,917	2.06	55,124	2.45	41.21
NO_NO7Ac	0.006	3,411,867	100,247	2.2	125,510	2.68	44.85
NO_NO6Ac	0.008	5,218,923	120,151	2.15	145,619	2.53	44.28
NO_NO8Ac	0.006	7,684,847	192,490	2.04	234,162	2.44	41.67
NO_NO9Ac	0.007	11,763,383	336,801	2.37	435,670	2.91	50.67
NO_NO11Ac	0.007	756,324	18,871	1.81	22,862	2.15	40
NO_NO10Ac	0.006	10,310,045	278,644	2.18	348,582	2.65	45.18
NO_NO12Ac	0.006	7,969,436	227,878	2.25	279,037	2.66	45.93
NO_NO13Ac	0.006	5,597,154	153,674	2.09	185,255	2.49	41.01
NO_NO14Ac	0.006	3,113,238	96,094	2.18	118,471	2.64	47.6
NO_NO15Ac	0.006	7,513,871	206,052	2.15	256,602	2.61	44.56
NO_NO16Ac	0.007	12,325,385	282,167	2.1	335,285	2.46	40.22
NO_NO17Ac	0.007	16,090,977	374,418	2.18	438,558	2.53	41.95
NO_NO18Ac	0.01	30,954	773	0.76	742	0.71	27.52
NO_NO19Ac	0.007	10,821,427	281,661	2.17	339,894	2.6	41.47
NO_NO20Ac	0.007	7,294,204	183,157	2.06	222,515	2.48	40.52
NO_NO21Ac	0.006	9,112,043	247,427	2.15	293,221	2.51	42.07
NO_NO22Ac	0.006	7,792,461	228,741	2.16	277,292	2.56	45.34
NO_NO23Ac	0.024	272,469	3,448	2.21	7,214	4.65	44.12
NO_NO24Ac	0.009	35,078	670	1.07	788	1.23	39.23
NO_NO25Ac	0.007	12,108,077	259,515	2.04	319,185	2.46	42.96
NO_NO27Ac	0.006	11,141,349	327,746	2.37	377,619	2.69	44.1
NO_NO28Ac	0.007	15,665,098	391,033	2.13	475,660	2.56	42.54
NO_NO29Ac	0.007	13,083,530	338,731	2.14	425,508	2.63	44.65
NO_NO30Ac	0.006	9,048,629	253,004	2.18	318,536	2.67	45.1
NO_NO31Ac	0.006	8,280,690	235,161	2.22	291,348	2.65	45.27
NO_NO32Ac	0.007	9,031,290	202,693	2.12	259,458	2.65	43.28
NO_NO33Ac	0.007	10,520,665	275,756	2.19	336,452	2.63	43.3
NO_NO34Ac	0.008	78,154	1,586	1.03	2,328	1.43	45.96
NO_NO35Ac	0.009	621,947	14,735	2.31	18,104	2.76	43.18
PL_PL2Ac	0.006	6,543,243	186,547	2.14	236,873	2.63	44.8
PL_PL3Ac	0.006	7,062,901	199,745	2.13	236,242	2.48	42.34
PL_PL1Ac	0.007	19,509,807	488,718	2.1	617,659	2.6	42.22
RU_RU3Ac	0.011	115,410	3,376	3.21	3,662	3.51	42.78
RU_RU1Ac	0.008	11,632,340	236,543	1.96	267,931	2.21	41.45

RU_RU4Ac	0.006	1,841,114	55,370	2.04	72,059	2.55	45.4
RU_RU2Ac	0.006	10,645,740	314,239	2.24	387,929	2.68	45.99
RU_RU5Ac	0.013	12,686,280	332,640	4.19	362,608	4.49	41.98
RU_RU6Ac	0.012	7,671,354	231,339	4.19	255,275	4.52	42.29
SK_SK1Ac	0.008	25,091,051	563,632	2.18	692,096	2.63	42.64
SK_SK2Ac	0.008	18,697,175	396,768	2.19	509,218	2.73	45.15
SK_SK3Ac	0.008	12,598,622	291,580	2.26	355,137	2.68	44.88
US_US1Ac	0.013	5,022,369	168,651	4.79	185,885	5.19	43.09
SK_SK4Ac	0.007	16,214,616	375,008	2.17	479,249	2.67	45.17
SK_SK5Ac	0.007	15,305,504	379,461	2.26	487,870	2.79	46.84

Analysis Parameters

Parameter	Value
Upstream Files Pattern	.F
Downstream Files Pattern	.R
Minimum Seed Length	19
Band Width	100
Z-dropoff	100
Trigger Re-seeding	1.5
Seed Occurrence	20
Skip Seeds	500
Drop Chains	0.5
Discard Chains	0
Mate Rescue Rounds	50
Skip Mate Rescue	false
Skip Pairing	false
Matching Score	1
Mismatch Penalty	4
Gap Open Penalty (DEL)	6
Gap Open Penalty (INS)	6
Gap Extension Penalty (DEL)	1
Gap Extension Penalty (INS)	1
5'-end Clipping Penalty	5
3'-end Clipping Penalty	5
Unpaired Read Penalty	17
Minimum Score	30
Split Alignments as Primary	false
MapQ of Supp. Alignments	false
Output All Alignments	false
Soft Clipping for Supp.	false
Shorter Split Hits as Secondary	false
Sort BAM File	By Coordinates
Add Read Group Information	false

References

- Li H. and Durbin R. (2009). Fast and accurate short read alignment with Burrows-Wheeler transform. *Bioinformatics (Oxford, England), 25*(14), 1754-60.
- Li H., Handsaker B., Wysoker A., Fennell T., Ruan J., Homer N., Marth G., Abecasis G. and Durbin R. (2009). The Sequence Alignment/Map format and SAMtools. *Bioinformatics (Oxford, England), 25*(16), 2078-9.

- Okonechnikov K., Conesa A. and Garcia-Alcalde F. (2016). Qualimap 2: advanced multi-sample quality control for high-throughput sequencing data. *Bioinformatics (Oxford, England), 32*(2), 292-4.
- OmicsBox - Bioinformatics made easy. BioBam Bioinformatics (Version 3.2.4). March 3, 2019. www.biobam.com/omicsbox.
