# Supplementary material for: Population Genomics of a Rare and a Common Wood–Inhabiting Fungal Species Across Europe
Source: Mol Ecol. 2026 Feb 6;35(3):e70260. doi: 10.1111/mec.70260 (PMC12878558; doi:10.1111/mec.70260)
Supplement: Supplementary file 3 — Supporting Information: S2 Read Alignment (BWA) results for Fomitopsis pinicola. [file MEC-35-e70260-s002.pdf]

Read Alignment (BWA) Results

Input 1: Reference Genome Sequences

Fompi3_AssemblyScaffolds

Sequences	Minimum Length	Maximum Length	Average Length	Total Length
504	1,004	1,378,269	82,568	41,614,358

Input 2: Sequencing Data

A total of 41 libraries have been processed.

Sample Name	Files	Sequencing	Format
AT_AT1Fp	AT_AT1Fp.F.fq.gz, AT_AT1Fp.R.fq.gz	Paired-End	FASTQ
AT_AT2Fp	AT_AT2Fp.F.fq.gz, AT_AT2Fp.R.fq.gz	Paired-End	FASTQ
CZ_CZ1Fp	CZ_CZ1Fp.F.fq.gz, CZ_CZ1Fp.R.fq.gz	Paired-End	FASTQ
CZ_CZ2Fp	CZ_CZ2Fp.F.fq.gz, CZ_CZ2Fp.R.fq.gz	Paired-End	FASTQ
CZ_CZ4Fp	CZ_CZ4Fp.F.fq.gz, CZ_CZ4Fp.R.fq.gz	Paired-End	FASTQ
CZ_CZ5Fp	CZ_CZ5Fp.F.fq.gz, CZ_CZ5Fp.R.fq.gz	Paired-End	FASTQ
CZ_CZ14Fp	CZ_CZ14Fp.F.fq.gz, CZ_CZ14Fp.R.fq.gz	Paired-End	FASTQ
CZ_CZ15Fp	CZ_CZ15Fp.F.fq.gz, CZ_CZ15Fp.R.fq.gz	Paired-End	FASTQ
CZ_CZ16Fp	CZ_CZ16Fp.F.fq.gz, CZ_CZ16Fp.R.fq.gz	Paired-End	FASTQ
CZ_CZ17Fp	CZ_CZ17Fp.F.fq.gz, CZ_CZ17Fp.R.fq.gz	Paired-End	FASTQ
CZ_CZ19Fp	CZ_CZ19Fp.F.fq.gz, CZ_CZ19Fp.R.fq.gz	Paired-End	FASTQ
CZ_CZ20Fp	CZ_CZ20Fp.F.fq.gz, CZ_CZ20Fp.R.fq.gz	Paired-End	FASTQ
CZ_CZ21Fp	CZ_CZ21Fp.F.fq.gz, CZ_CZ21Fp.R.fq.gz	Paired-End	FASTQ
CZ_CZ22Fp	CZ_CZ22Fp.F.fq.gz, CZ_CZ22Fp.R.fq.gz	Paired-End	FASTQ
CZ_CZ23Fp	CZ_CZ23Fp.F.fq.gz, CZ_CZ23Fp.R.fq.gz	Paired-End	FASTQ
CZ_CZ24Fp	CZ_CZ24Fp.F.fq.gz, CZ_CZ24Fp.R.fq.gz	Paired-End	FASTQ
DE_DE1Fp	DE_DE1Fp.F.fq.gz, DE_DE1Fp.R.fq.gz	Paired-End	FASTQ
DE_DE2Fp	DE_DE2Fp.F.fq.gz, DE_DE2Fp.R.fq.gz	Paired-End	FASTQ
DE_DE8Fp	DE_DE8Fp.F.fq.gz, DE_DE8Fp.R.fq.gz	Paired-End	FASTQ
DE_DE9Fp	DE_DE9Fp.F.fq.gz, DE_DE9Fp.R.fq.gz	Paired-End	FASTQ
DE_DE13Fp	DE_DE13Fp.F.fq.gz, DE_DE13Fp.R.fq.gz	Paired-End	FASTQ
EE_EE1Fp	EE_EE1Fp.F.fq.gz, EE_EE1Fp.R.fq.gz	Paired-End	FASTQ
EE_EE2Fp	EE_EE2Fp.F.fq.gz, EE_EE2Fp.R.fq.gz	Paired-End	FASTQ
EE_EE3Fp	EE_EE3Fp.F.fq.gz, EE_EE3Fp.R.fq.gz	Paired-End	FASTQ
EE_EE4Fp	EE_EE4Fp.F.fq.gz, EE_EE4Fp.R.fq.gz	Paired-End	FASTQ
EE_EE5Fp	EE_EE5Fp.F.fq.gz, EE_EE5Fp.R.fq.gz	Paired-End	FASTQ
FI_FI1Fp	FI_FI1Fp.F.fq.gz, FI_FI1Fp.R.fq.gz	Paired-End	FASTQ
FI_FI2Fp	FI_FI2Fp.F.fq.gz, FI_FI2Fp.R.fq.gz	Paired-End	FASTQ
FI_FI3Fp	FI_FI3Fp.F.fq.gz, FI_FI3Fp.R.fq.gz	Paired-End	FASTQ
HR_HR1Fp	HR_HR1Fp.F.fq.gz, HR_HR1Fp.R.fq.gz	Paired-End	FASTQ
HR_HR5Fp	HR_HR5Fp.F.fq.gz, HR_HR5Fp.R.fq.gz	Paired-End	FASTQ
HR_HR12Fp	HR_HR12Fp.F.fq.gz, HR_HR12Fp.R.fq.gz	Paired-End	FASTQ
LT_LT1Fp	LT_LT1Fp.F.fq.gz, LT_LT1Fp.R.fq.gz	Paired-End	FASTQ
LT_LT2Fp	LT_LT2Fp.F.fq.gz, LT_LT2Fp.R.fq.gz	Paired-End	FASTQ
LT_LT3Fp	LT_LT3Fp.F.fq.gz, LT_LT3Fp.R.fq.gz	Paired-End	FASTQ
LT_LT4Fp	LT_LT4Fp.F.fq.gz, LT_LT4Fp.R.fq.gz	Paired-End	FASTQ
SK_SK1Fp	SK_SK1Fp.F.fq.gz, SK_SK1Fp.R.fq.gz	Paired-End	FASTQ
SK_SK2Fp	SK_SK2Fp.F.fq.gz, SK_SK2Fp.R.fq.gz	Paired-End	FASTQ
SK_SK3Fp	SK_SK3Fp.F.fq.gz, SK_SK3Fp.R.fq.gz	Paired-End	FASTQ
SK_SK4Fp	SK_SK4Fp.F.fq.gz, SK_SK4Fp.R.fq.gz	Paired-End	FASTQ
SK_SK5Fp	SK_SK5Fp.F.fq.gz, SK_SK5Fp.R.fq.gz	Paired-End	FASTQ

Results Overview

Globals

Sample	Total Alignments	Mapped	Supplementary	Unmapped	Duplicated Reads (estimated)	Duplication Rate
AT_AT1Fp	9,376,120	7,790,604 / 83.09%	129,994 / 1.386%	1,585,516 / 16.91%	3,514,665 / 37.485%	34.97
AT_AT2Fp	7,981,294	6,674,355 / 83.625%	146,140 / 1.831%	1,306,939 / 16.375%	2,983,983 / 37.387%	34.44

CZ_CZ1Fp	10,710,992	9,030,170 / 84.308%	143,524 / 1.34%	1,680,822 / 15.692%	4,110,404 / 38.376%	35.29
CZ_CZ14Fp	8,604,069	7,275,674 / 84.561%	148,101 / 1.721%	1,328,395 / 15.439%	3,326,239 / 38.659%	34.89
CZ_CZ16Fp	6,721,615	5,571,949 / 82.896%	109,099 / 1.623%	1,149,666 / 17.104%	2,512,384 / 37.378%	35.92
CZ_CZ2Fp	20,136,223	16,963,116 / 84.242%	282,273 / 1.402%	3,173,107 / 15.758%	9,236,729 / 45.871%	42.7
CZ_CZ4Fp	10,616,678	8,835,404 / 83.222%	151,114 / 1.423%	1,781,274 / 16.778%	4,117,851 / 38.787%	34.62
CZ_CZ5Fp	16,098,574	13,798,314 / 85.711%	216,532 / 1.345%	2,300,260 / 14.289%	6,968,770 / 43.288%	39.46
CZ_CZ15Fp	8,634,629	7,104,125 / 82.275%	133,679 / 1.548%	1,530,504 / 17.725%	3,361,139 / 38.926%	35.79
CZ_CZ17Fp	8,911,829	7,323,100 / 82.173%	139,321 / 1.563%	1,588,729 / 17.827%	3,429,857 / 38.487%	36.69
CZ_CZ19Fp	5,560,611	4,623,602 / 83.149%	73,675 / 1.325%	937,009 / 16.851%	1,878,975 / 33.791%	31.96
CZ_CZ20Fp	3,210,893	2,718,476 / 84.664%	45,289 / 1.41%	492,417 / 15.336%	962,554 / 29.978%	29.08
CZ_CZ21Fp	4,458,450	3,816,752 / 85.607%	63,672 / 1.428%	641,698 / 14.393%	1,504,516 / 33.745%	31.99
CZ_CZ22Fp	3,406,538	2,664,264 / 78.21%	147,562 / 4.332%	742,274 / 21.79%	1,435,922 / 42.152%	53.27
CZ_CZ23Fp	5,634,237	4,757,251 / 84.435%	81,735 / 1.451%	876,986 / 15.565%	1,961,559 / 34.815%	33.16
CZ_CZ24Fp	9,102,293	7,733,875 / 84.966%	134,197 / 1.474%	1,368,418 / 15.034%	3,659,133 / 40.2%	37.9
DE_DE1Fp	7,593,891	6,313,483 / 83.139%	113,267 / 1.492%	1,280,408 / 16.861%	2,762,451 / 36.377%	35.13
DE_DE13Fp	5,660,670	4,623,871 / 81.684%	92,136 / 1.628%	1,036,799 / 18.316%	2,089,080 / 36.905%	38.82
DE_DE2Fp	6,461,956	5,197,722 / 80.436%	93,012 / 1.439%	1,264,234 / 19.564%	2,200,447 / 34.052%	33.33
DE_DE8Fp	5,578,204	4,708,104 / 84.402%	134,346 / 2.408%	870,100 / 15.598%	2,018,361 / 36.183%	34.7
DE_DE9Fp	6,183,509	5,028,844 / 81.327%	100,071 / 1.618%	1,154,665 / 18.673%	2,126,404 / 34.388%	34.12
EE_EE4Fp	4,860,232	4,099,671 / 84.351%	66,154 / 1.361%	760,561 / 15.649%	1,664,749 / 34.252%	33.12
EE_EE2Fp	7,226,014	6,065,016 / 83.933%	103,014 / 1.426%	1,160,998 / 16.067%	2,616,803 / 36.214%	32.5
EE_EE3Fp	6,371,340	5,264,156 / 82.622%	106,700 / 1.675%	1,107,184 / 17.378%	2,540,283 / 39.87%	40.87
EE_EE5Fp	7,923,431	6,745,967 / 85.139%	113,195 / 1.429%	1,177,464 / 14.861%	3,094,757 / 39.058%	35.52
FI_FI2Fp	6,972,922	5,860,102 / 84.041%	98,234 / 1.409%	1,112,820 / 15.959%	2,523,477 / 36.19%	32.82
HR_HR1Fp	4,476,284	3,324,463 / 74.268%	79,652 / 1.779%	1,151,821 / 25.732%	2,868,851 / 64.09%	88.14
EE_EE1Fp	10,277,268	8,574,533 / 83.432%	141,022 / 1.372%	1,702,735 / 16.568%	4,269,216 / 41.54%	37.65
FI_FI1Fp	9,812,783	8,242,351 / 83.996%	139,513 / 1.422%	1,570,432 / 16.004%	4,011,059 / 40.876%	36.73
FI_FI3Fp	6,943,162	5,797,213 / 83.495%	102,996 / 1.483%	1,145,949 / 16.505%	2,547,870 / 36.696%	33.08
HR_HR5Fp	8,102,870	7,158,143 / 88.341%	109,578 / 1.352%	944,727 / 11.659%	3,324,007 / 41.023%	39.18
LT_LT1Fp	5,212,405	207,307 / 3.977%	6,719 / 0.129%	5,005,098 / 96.023%	167,710 / 3.218%	87.65
HR_HR12Fp	7,942,686	6,749,227 / 84.974%	108,540 / 1.367%	1,193,459 / 15.026%	3,088,948 / 38.89%	36.68

LT_LT2Fp	7,966,394	6,756,258 / 84.809%	120,042 / 1.507%	1,210,136 / 15.191%	3,135,918 / 39.364%	35.09
LT_LT3Fp	5,503,527	4,060,468 / 73.779%	73,461 / 1.335%	1,443,059 / 26.221%	1,652,237 / 30.021%	32.66
SK_SK1Fp	4,047,436	3,415,901 / 84.397%	58,322 / 1.441%	631,535 / 15.603%	1,308,212 / 32.322%	30.04
LT_LT4Fp	9,207,134	7,786,093 / 84.566%	159,174 / 1.729%	1,421,041 / 15.434%	3,690,488 / 40.083%	36.04
SK_SK2Fp	11,078,156	9,346,560 / 84.369%	158,372 / 1.43%	1,731,596 / 15.631%	4,618,619 / 41.691%	38.18
SK_SK3Fp	6,460,099	5,425,275 / 83.981%	106,253 / 1.645%	1,034,824 / 16.019%	2,352,675 / 36.419%	35.45
SK_SK4Fp	7,613,668	312,937 / 4.11%	12,362 / 0.162%	7,300,731 / 95.89%	250,654 / 3.292%	57.53
SK_SK5Fp	9,818,908	8,433,736 / 85.893%	124,068 / 1.264%	1,385,172 / 14.107%	4,006,682 / 40.806%	37.23

Paired Information

Sample	First in pair	Second in pair	Both in pair	Singletons
AT_AT1Fp	3,890,637 / 41.495%	3,899,967 / 41.595%	7,631,095 / 81.389%	159,509 / 1.701%
AT_AT2Fp	3,312,861 / 41.508%	3,361,494 / 42.117%	6,515,814 / 81.639%	158,541 / 1.986%
CZ_CZ1Fp	4,513,243 / 42.137%	4,516,927 / 42.171%	8,824,500 / 82.387%	205,670 / 1.92%
CZ_CZ14Fp	3,620,098 / 42.074%	3,655,576 / 42.487%	7,163,818 / 83.261%	111,856 / 1.3%
CZ_CZ16Fp	2,783,437 / 41.41%	2,788,512 / 41.486%	5,511,958 / 82.003%	59,991 / 0.893%
CZ_CZ2Fp	8,483,703 / 42.132%	8,479,413 / 42.11%	16,557,258 / 82.226%	405,858 / 2.016%
CZ_CZ4Fp	4,410,047 / 41.539%	4,425,357 / 41.683%	8,622,178 / 81.214%	213,226 / 2.008%
CZ_CZ5Fp	6,902,639 / 42.877%	6,895,675 / 42.834%	13,514,577 / 83.949%	283,737 / 1.762%
CZ_CZ15Fp	3,544,504 / 41.05%	3,559,621 / 41.225%	6,982,877 / 80.871%	121,248 / 1.404%
CZ_CZ17Fp	3,659,586 / 41.064%	3,663,514 / 41.108%	7,198,183 / 80.771%	124,917 / 1.402%
CZ_CZ19Fp	2,309,165 / 41.527%	2,314,437 / 41.622%	4,541,684 / 81.676%	81,918 / 1.473%
CZ_CZ20Fp	1,358,056 / 42.295%	1,360,420 / 42.369%	2,679,208 / 83.441%	39,268 / 1.223%
CZ_CZ21Fp	1,906,951 / 42.772%	1,909,801 / 42.836%	3,771,819 / 84.599%	44,933 / 1.008%
CZ_CZ22Fp	1,345,688 / 39.503%	1,318,576 / 38.707%	2,643,291 / 77.595%	20,973 / 0.616%
CZ_CZ23Fp	2,376,055 / 42.172%	2,381,196 / 42.263%	4,684,326 / 83.14%	72,925 / 1.294%
CZ_CZ24Fp	3,863,235 / 42.442%	3,870,640 / 42.524%	7,626,560 / 83.787%	107,315 / 1.179%
DE_DE1Fp	3,151,571 / 41.501%	3,161,912 / 41.638%	6,229,098 / 82.028%	84,385 / 1.111%
DE_DE13Fp	2,311,460 / 40.834%	2,312,411 / 40.85%	4,583,581 / 80.972%	40,290 / 0.712%
DE_DE2Fp	2,596,695 / 40.184%	2,601,027 / 40.251%	5,106,225 / 79.02%	91,497 / 1.416%
DE_DE8Fp	2,332,853 / 41.821%	2,375,251 / 42.581%	4,645,068 / 83.272%	63,036 / 1.13%
DE_DE9Fp	2,513,514 / 40.649%	2,515,330 / 40.678%	4,965,315 / 80.299%	63,529 / 1.027%
EE_EE4Fp	2,048,160 / 42.141%	2,051,511 / 42.21%	4,036,872 / 83.059%	62,799 / 1.292%
EE_EE2Fp	3,028,645 / 41.913%	3,036,371 / 42.02%	5,954,926 / 82.41%	110,090 / 1.524%
EE_EE3Fp	2,630,636 / 41.289%	2,633,520 / 41.334%	5,210,517 / 81.781%	53,639 / 0.842%
EE_EE5Fp	3,370,973 / 42.544%	3,374,994 / 42.595%	6,642,551 / 83.834%	103,416 / 1.305%
FI_FI2Fp	2,927,079 / 41.978%	2,933,023 / 42.063%	5,759,477 / 82.598%	100,625 / 1.443%
HR_HR1Fp	1,663,785 / 37.169%	1,660,678 / 37.099%	3,302,141 / 73.77%	22,322 / 0.499%
EE_EE1Fp	4,283,286 / 41.677%	4,291,247 / 41.755%	8,402,300 / 81.756%	172,233 / 1.676%
FI_FI1Fp	4,121,955 / 42.006%	4,120,396 / 41.99%	8,065,207 / 82.191%	177,144 / 1.805%
FI_FI3Fp	2,898,222 / 41.742%	2,898,991 / 41.753%	5,668,391 / 81.64%	128,822 / 1.855%
HR_HR5Fp	3,577,334 / 44.149%	3,580,809 / 44.192%	7,075,247 / 87.318%	82,896 / 1.023%
LT_LT1Fp	102,405 / 1.965%	104,902 / 2.013%	189,991 / 3.645%	17,316 / 0.332%
HR_HR12Fp	3,370,206 / 42.432%	3,379,021 / 42.543%	6,671,620 / 83.997%	77,607 / 0.977%
LT_LT2Fp	3,373,055 / 42.341%	3,383,203 / 42.468%	6,627,667 / 83.195%	128,591 / 1.614%
LT_LT3Fp	2,029,222 / 36.871%	2,031,246 / 36.908%	3,988,412 / 72.47%	72,056 / 1.309%
SK_SK1Fp	1,705,790 / 42.145%	1,710,111 / 42.252%	3,345,998 / 82.67%	69,903 / 1.727%
LT_LT4Fp	3,866,648 / 41.996%	3,919,445 / 42.57%	7,648,390 / 83.07%	137,703 / 1.496%
SK_SK2Fp	4,674,949 / 42.2%	4,671,611 / 42.17%	9,134,374 / 82.454%	212,186 / 1.915%
SK_SK3Fp	2,710,083 / 41.951%	2,715,192 / 42.03%	5,366,696 / 83.075%	58,579 / 0.907%
SK_SK4Fp	149,714 / 1.966%	163,223 / 2.144%	255,252 / 3.353%	57,685 / 0.758%
SK_SK5Fp	4,216,778 / 42.945%	4,216,958 / 42.947%	8,300,888 / 84.54%	132,848 / 1.353%

ACTG Content

Sample	A's	C's	T's	G's	N's	GC (%)
AT_AT1Fp	230,182,367 / 21.961%	294,569,291 / 28.104%	230,440,229 / 21.986%	292,944,834 / 27.949%	37,022 / 0.004%	56.05
AT_AT2Fp	196,391,339 / 21.956%	252,014,110 / 28.175%	196,498,562 / 21.968%	249,558,853 / 27.9%	30,119 / 0.003%	56.08

CZ_CZ1Fp	278,536,867 / 22.227%	348,652,519 / 27.822%	278,564,538 / 22.229%	347,413,542 / 27.723%	42,727 / 0.003%	55.54
CZ_CZ14Fp	213,104,787 / 22.043%	271,175,475 / 28.049%	213,296,449 / 22.062%	269,207,314 / 27.846%	34,258 / 0.004%	55.89
CZ_CZ16Fp	159,690,677 / 22.212%	200,336,798 / 27.866%	159,947,897 / 22.248%	198,957,046 / 27.674%	24,793 / 0.003%	55.54
CZ_CZ2Fp	523,161,613 / 22.269%	652,601,569 / 27.779%	523,428,115 / 22.28%	650,103,406 / 27.672%	82,157 / 0.003%	55.45
CZ_CZ4Fp	266,320,450 / 22.065%	337,766,353 / 27.984%	266,533,764 / 22.083%	336,356,935 / 27.868%	38,215 / 0.003%	55.85
CZ_CZ5Fp	422,676,132 / 22.111%	534,066,726 / 27.938%	422,844,101 / 22.119%	532,057,450 / 27.832%	66,695 / 0.003%	55.77
CZ_CZ15Fp	206,930,247 / 22.127%	261,247,898 / 27.936%	207,005,926 / 22.136%	259,990,231 / 27.801%	30,547 / 0.003%	55.74
CZ_CZ17Fp	212,376,932 / 22.064%	269,498,516 / 27.998%	212,739,605 / 22.101%	267,943,634 / 27.837%	32,796 / 0.003%	55.83
CZ_CZ19Fp	136,243,738 / 21.717%	177,707,912 / 28.326%	136,345,272 / 21.733%	177,074,113 / 28.225%	21,484 / 0.003%	56.55
CZ_CZ20Fp	77,772,260 / 21.483%	103,439,051 / 28.573%	77,787,488 / 21.488%	103,011,777 / 28.455%	12,614 / 0.003%	57.03
CZ_CZ21Fp	107,518,799 / 21.492%	142,978,206 / 28.58%	107,532,564 / 21.495%	142,235,615 / 28.432%	16,092 / 0.003%	57.01
CZ_CZ22Fp	53,253,190 / 21.823%	70,566,123 / 28.918%	53,347,062 / 21.861%	66,857,210 / 27.398%	7,855 / 0.003%	56.32
CZ_CZ23Fp	138,961,507 / 22.074%	176,164,630 / 27.984%	139,120,458 / 22.1%	175,265,517 / 27.841%	19,740 / 0.003%	55.83
CZ_CZ24Fp	226,418,104 / 22.145%	285,395,610 / 27.914%	226,674,957 / 22.171%	283,927,712 / 27.77%	35,782 / 0.003%	55.68
DE_DE1Fp	182,623,957 / 22.063%	231,867,753 / 28.012%	182,950,006 / 22.102%	230,304,756 / 27.823%	28,218 / 0.003%	55.84
DE_DE13Fp	123,549,983 / 21.817%	160,197,823 / 28.288%	123,590,985 / 21.824%	158,970,672 / 28.071%	19,688 / 0.003%	56.36
DE_DE2Fp	152,112,478 / 22.136%	191,858,424 / 27.92%	152,303,809 / 22.164%	190,905,407 / 27.781%	23,923 / 0.003%	55.7
DE_DE8Fp	130,212,219 / 21.939%	167,671,161 / 28.251%	130,348,814 / 21.962%	165,280,104 / 27.848%	21,382 / 0.004%	56.1
DE_DE9Fp	142,838,769 / 22.057%	181,593,831 / 28.042%	142,949,581 / 22.075%	180,195,343 / 27.826%	22,005 / 0.003%	55.87
EE_EE4Fp	119,228,490 / 21.644%	156,489,820 / 28.408%	119,261,411 / 21.65%	155,880,376 / 28.298%	19,265 / 0.003%	56.71
EE_EE2Fp	181,672,763 / 22.073%	230,180,351 / 27.967%	182,086,544 / 22.123%	229,107,033 / 27.836%	29,263 / 0.004%	55.8
EE_EE3Fp	148,865,302 / 22.128%	188,321,921 / 27.993%	149,230,615 / 22.182%	186,334,642 / 27.697%	22,024 / 0.003%	55.69
EE_EE5Fp	198,368,437 / 22.008%	252,768,603 / 28.044%	198,671,227 / 22.042%	251,532,920 / 27.907%	30,069 / 0.003%	55.95
FI_FI2Fp	172,123,375 / 21.93%	220,709,563 / 28.12%	172,183,678 / 21.938%	219,863,717 / 28.012%	26,251 / 0.003%	56.13
HR_HR1Fp	82,774,528 / 22.046%	105,800,038 / 28.178%	82,784,812 / 22.049%	104,104,870 / 27.727%	11,802 / 0.003%	55.91
EE_EE1Fp	256,621,483 / 22.055%	325,871,775 / 28.007%	256,591,977 / 22.053%	324,456,801 / 27.885%	40,666 / 0.003%	55.89
FI_FI1Fp	243,379,274 / 21.995%	310,394,480 / 28.051%	243,519,253 / 22.008%	309,234,844 / 27.946%	38,653 / 0.003%	56
FI_FI3Fp	172,051,230 / 22.078%	217,984,335 / 27.972%	172,315,747 / 22.112%	216,939,351 / 27.838%	25,243 / 0.003%	55.81
HR_HR5Fp	210,361,716 / 21.796%	272,898,929 / 28.276%	210,491,086 / 21.81%	271,370,484 / 28.118%	31,901 / 0.003%	56.39
LT_LT1Fp	755,736 / 14.486%	2,281,368 / 43.73%	742,750 / 14.237%	1,437,042 / 27.546%	68 / 0.001%	71.28
HR_HR12Fp	199,406,783 / 22.139%	251,516,773 / 27.924%	199,348,287 / 22.132%	250,451,443 / 27.806%	30,610 / 0.003%	55.73

LT_LT2Fp	199,194,595 / 22.014%	253,653,096 / 28.032%	199,595,644 / 22.058%	252,413,396 / 27.895%	31,603 / 0.003%	55.93
LT_LT3Fp	116,813,935 / 22.006%	149,017,437 / 28.072%	117,032,920 / 22.047%	147,970,768 / 27.875%	17,826 / 0.003%	55.95
SK_SK1Fp	99,602,208 / 21.587%	131,301,072 / 28.457%	99,641,275 / 21.595%	130,857,303 / 28.361%	15,362 / 0.003%	56.82
LT_LT4Fp	228,583,370 / 21.903%	294,460,130 / 28.216%	228,807,840 / 21.925%	291,755,937 / 27.956%	35,196 / 0.003%	56.17
SK_SK2Fp	280,220,145 / 22.098%	354,405,136 / 27.948%	280,088,074 / 22.088%	353,369,211 / 27.866%	42,521 / 0.003%	55.81
SK_SK3Fp	152,407,423 / 22.003%	194,459,471 / 28.074%	152,453,284 / 22.009%	193,351,443 / 27.914%	23,431 / 0.003%	55.99
SK_SK4Fp	2,925,216 / 20.126%	4,634,743 / 31.888%	2,906,924 / 20%	4,067,388 / 27.985%	133 / 0.001%	59.87
SK_SK5Fp	250,402,654 / 21.921%	321,189,098 / 28.118%	250,633,214 / 21.941%	320,056,039 / 28.019%	39,666 / 0.003%	56.14

Coverage

Sample	Mean	Standard Deviation
AT_AT1Fp	25.283X	57.386X
AT_AT2Fp	21.575X	72.619X
CZ_CZ1Fp	30.235X	56.132X
CZ_CZ14Fp	23.323X	64.752X
CZ_CZ16Fp	17.343X	48.803X
CZ_CZ2Fp	56.684X	112.69X
CZ_CZ4Fp	29.119X	66.868X
CZ_CZ5Fp	46.117X	87.006X
CZ_CZ15Fp	22.559X	60.595X
CZ_CZ17Fp	23.222X	61.41X
CZ_CZ19Fp	15.135X	31.421X
CZ_CZ20Fp	8.733X	19.398X
CZ_CZ21Fp	12.066X	31.495X
CZ_CZ22Fp	5.881X	102.721X
CZ_CZ23Fp	15.186X	34.071X
CZ_CZ24Fp	24.665X	54.99X
DE_DE1Fp	19.969X	51.401X
DE_DE13Fp	13.659X	42.932X
DE_DE2Fp	16.578X	42.129X
DE_DE8Fp	14.317X	77.515X
DE_DE9Fp	15.621X	47.731X
EE_EE4Fp	13.287X	29.23X
EE_EE2Fp	19.855X	47.199X
EE_EE3Fp	16.228X	52.349X
EE_EE5Fp	21.739X	53.877X
FI_FI2Fp	18.935X	40.036X
HR_HR1Fp	9.054X	51.558X
EE_EE1Fp	28.066X	65.451X
FI_FI1Fp	26.69X	62.861X
FI_FI3Fp	18.799X	45.556X
HR_HR5Fp	23.286X	45.298X
LT_LT1Fp	0.125X	25.355X
HR_HR12Fp	21.729X	46.084X
LT_LT2Fp	21.825X	55.805X
LT_LT3Fp	12.805X	35.713X
SK_SK1Fp	11.13X	25.505X
LT_LT4Fp	25.172X	78.725X
SK_SK2Fp	30.595X	61.063X
SK_SK3Fp	16.71X	41.552X
SK_SK4Fp	0.349X	17.836X
SK_SK5Fp	27.556X	54.624X

Mappping Quality

Sample	Mean Mapping Quality
AT_AT1Fp	50.733
AT_AT2Fp	49.953
CZ_CZ1Fp	50.329
CZ_CZ14Fp	50.086
CZ_CZ16Fp	50.826
CZ_CZ2Fp	50.616
CZ_CZ4Fp	49.249
CZ_CZ5Fp	49.644
CZ_CZ15Fp	50.336
CZ_CZ17Fp	51.328
CZ_CZ19Fp	50.193
CZ_CZ20Fp	51.006
CZ_CZ21Fp	51.231
CZ_CZ22Fp	49.952
CZ_CZ23Fp	51.401
CZ_CZ24Fp	50.1
DE_DE1Fp	51.573
DE_DE13Fp	50.514
DE_DE2Fp	50.278
DE_DE8Fp	51.115
DE_DE9Fp	51.677
EE_EE4Fp	50.786
EE_EE2Fp	50.751
EE_EE3Fp	51.307
EE_EE5Fp	50.614
FI_FI2Fp	50.817
HR_HR1Fp	50.778
EE_EE1Fp	51.199
FI_FI1Fp	50.888
FI_FI3Fp	50.508
HR_HR5Fp	49.202
LT_LT1Fp	36.865
HR_HR12Fp	50.106
LT_LT2Fp	50.472
LT_LT3Fp	51.353
SK_SK1Fp	50.613
LT_LT4Fp	51.213
SK_SK2Fp	51.021
SK_SK3Fp	51.705
SK_SK4Fp	37.908
SK_SK5Fp	50.903

Insert Size

Sample	Mean	Standard Deviation	Median
AT_AT1Fp	546.875	4,748.951	304
AT_AT2Fp	594.619	5,562.237	321
CZ_CZ1Fp	633.954	5,097.94	337
CZ_CZ14Fp	455.869	4,981.852	235
CZ_CZ16Fp	344.261	3,813.123	191
CZ_CZ2Fp	663.012	5,396.673	347
CZ_CZ4Fp	624.941	5,581.589	313
CZ_CZ5Fp	587.789	5,300.93	304
CZ_CZ15Fp	443.189	4,353.951	240
CZ_CZ17Fp	467.864	4,645.114	239
CZ_CZ19Fp	501.851	4,752.9	272
CZ_CZ20Fp	436.312	4,279.967	246
CZ_CZ21Fp	407.504	4,955.099	226
CZ_CZ22Fp	248.496	5,729.677	94
CZ_CZ23Fp	435.473	3,953.166	240
CZ_CZ24Fp	434.046	4,233.088	235
DE_DE1Fp	423.583	4,876.986	228
DE_DE13Fp	307.908	4,049.215	164
DE_DE2Fp	441.313	4,379.686	248
DE_DE8Fp	374.635	4,516.666	200
DE_DE9Fp	377.117	4,220.164	210
EE_EE4Fp	444.631	4,059.159	257
EE_EE2Fp	497.966	4,287.716	275
EE_EE3Fp	333.47	3,761.306	201
EE_EE5Fp	467.052	4,422.348	258
FI_FI2Fp	456.887	4,472.273	261
HR_HR1Fp	242.726	3,982.645	133
EE_EE1Fp	532.66	4,979.153	277
FI_FI1Fp	541.143	4,786.126	301
FI_FI3Fp	547.244	4,934.373	278
HR_HR5Fp	448.026	4,421.818	257
LT_LT1Fp	25.64	101.522	19
HR_HR12Fp	390.57	4,134.274	233
LT_LT2Fp	516.121	4,646.721	280
LT_LT3Fp	454.766	4,612.193	249
SK_SK1Fp	548.528	4,988.001	287
LT_LT4Fp	484.755	4,387.224	269
SK_SK2Fp	593.228	5,692.314	294
SK_SK3Fp	363.604	4,969.971	191
SK_SK4Fp	124.609	1,394.2	24
SK_SK5Fp	519.767	4,851.676	277

Mismatches and Indels

Sample	General Error Rate	Mismatches	Insertions	Mapped Reads with Insertion (%)	Deletions	Mapped Reds with Deletion (%)	Homopolymer Indels (%)
AT_AT1Fp	0.039	37,631,810	918,949	10.28	907,746	10.08	32.12
AT_AT2Fp	0.039	32,093,869	770,239	10.06	761,144	9.87	32.11
CZ_CZ1Fp	0.04	45,798,340	1,133,934	10.88	1,130,959	10.75	32.04
CZ_CZ14Fp	0.039	34,414,079	858,721	10.29	849,828	10.09	32.14
CZ_CZ16Fp	0.039	25,359,681	634,810	9.94	623,893	9.72	31.97
CZ_CZ2Fp	0.04	85,205,244	2,179,806	11.12	2,147,083	10.89	32.13
CZ_CZ4Fp	0.042	46,056,204	1,089,212	10.72	1,079,412	10.54	31.91
CZ_CZ5Fp	0.04	69,060,970	1,718,977	10.8	1,697,349	10.6	32.1
CZ_CZ15Fp	0.039	33,725,878	821,628	10.1	812,129	9.89	32.28
CZ_CZ17Fp	0.039	34,251,524	862,180	10.26	856,268	10.08	32.11
CZ_CZ19Fp	0.04	22,596,678	554,599	10.42	549,014	10.24	32.15
CZ_CZ20Fp	0.039	12,740,028	313,918	10.06	310,857	9.88	32.17
CZ_CZ21Fp	0.038	17,251,736	420,571	9.63	415,335	9.44	32.03
CZ_CZ22Fp	0.033	7,529,650	162,436	5.52	166,987	5.65	34.32
CZ_CZ23Fp	0.039	22,265,221	563,369	10.33	554,357	10.09	32.08
CZ_CZ24Fp	0.039	36,076,631	907,340	10.23	901,398	10.07	32.27
DE_DE1Fp	0.039	29,122,733	749,463	10.31	737,974	10.11	32.13
DE_DE13Fp	0.037	19,332,851	480,642	9.11	476,287	8.98	32.08
DE_DE2Fp	0.039	24,533,663	621,750	10.41	612,618	10.18	32.31
DE_DE8Fp	0.038	20,576,320	520,925	9.66	510,490	9.43	32.07
DE_DE9Fp	0.038	22,576,425	569,771	9.91	563,063	9.7	32.18
EE_EE4Fp	0.038	19,138,961	471,231	10.02	465,728	9.82	32.47
EE_EE2Fp	0.039	29,344,194	726,033	10.41	718,212	10.23	32.34
EE_EE3Fp	0.038	23,348,148	577,909	9.62	576,233	9.52	33.16
EE_EE5Fp	0.038	31,391,098	757,775	9.79	749,862	9.64	32.34
FI_FI2Fp	0.039	28,094,611	693,652	10.31	690,154	10.18	32.04
HR_HR1Fp	0.037	12,629,702	297,868	7.98	301,037	7.98	32.87
EE_EE1Fp	0.039	41,408,205	999,218	10.14	986,111	9.93	32.44
FI_FI1Fp	0.039	39,268,293	960,200	10.16	940,659	9.86	32.33
FI_FI3Fp	0.04	28,324,651	685,027	10.33	679,168	10.15	32.13
HR_HR5Fp	0.039	33,921,916	876,188	10.63	866,888	10.43	31.71
LT_LT1Fp	0.007	36,577	818	0.35	701	0.3	29.3
HR_HR12Fp	0.039	31,782,889	798,243	10.28	795,300	10.18	32.17
LT_LT2Fp	0.039	32,020,071	769,116	9.95	762,633	9.8	32.25
LT_LT3Fp	0.038	18,568,834	463,358	9.97	458,197	9.77	31.96
SK_SK1Fp	0.039	16,599,152	400,618	10.19	393,788	9.94	32.36
LT_LT4Fp	0.038	36,588,278	899,103	10.08	884,678	9.84	32.44
SK_SK2Fp	0.04	46,712,345	1,166,602	10.83	1,145,184	10.56	31.97
SK_SK3Fp	0.039	24,521,201	610,616	9.85	605,506	9.69	32.08
SK_SK4Fp	0.071	1,014,708	4,897	1.34	4,057	1.16	23.83
SK_SK5Fp	0.039	40,746,434	1,002,116	10.34	996,848	10.21	31.92

Analysis Parameters

Parameter	Value
Upstream Files Pattern	.F
Downstream Files Pattern	.R
Minimum Seed Length	19
Band Width	100
Z-dropoff	100
Trigger Re-seeding	1.5
Seed Occurrence	20
Skip Seeds	500
Drop Chains	0.5
Discard Chains	0
Mate Rescue Rounds	50
Skip Mate Rescue	false
Skip Pairing	false
Matching Score	1
Mismatch Penalty	4
Gap Open Penalty (DEL)	6
Gap Open Penalty (INS)	6
Gap Extension Penalty (DEL)	1
Gap Extension Penalty (INS)	1
5'-end Clipping Penalty	5
3'-end Clipping Penalty	5
Unpaired Read Penalty	17
Minimum Score	30
Split Alignments as Primary	false
MapQ of Supp. Alignments	false
Output All Alignments	false
Soft Clipping for Supp.	false
Shorter Split Hits as Secondary	false
Sort BAM File	By Coordinates
Add Read Group Information	false

References

- Li H. and Durbin R. (2009). Fast and accurate short read alignment with Burrows-Wheeler transform. *Bioinformatics (Oxford, England), 25*(14), 1754-60.
- Li H., Handsaker B., Wysoker A., Fennell T., Ruan J., Homer N., Marth G., Abecasis G. and Durbin R. (2009). The Sequence Alignment/Map format and SAMtools. *Bioinformatics (Oxford, England), 25*(16), 2078-9.
- Okonechnikov K., Conesa A. and Garcia-Alcalde F. (2016). Qualimap 2: advanced multi-sample quality control for high-throughput sequencing data. *Bioinformatics (Oxford, England), 32*(2), 292-4.
- OmicsBox - Bioinformatics made easy. BioBam Bioinformatics (Version 3.1.11). March 3, 2019. www.biobam.com/omicsbox.
