# Supplementary material for: Population Genomics of a Rare and a Common Wood–Inhabiting Fungal Species Across Europe
Source: Mol Ecol. 2026 Feb 6;35(3):e70260. doi: 10.1111/mec.70260 (PMC12878558; doi:10.1111/mec.70260)
Supplement: Supplementary file 4 — Supporting Information: S3 Bioinformatics pipeline. [file MEC-35-e70260-s004.pdf]

```
# VCF Filtering Pipeline for Antrodiella citrinella (or Fompitopsis
pinicola)
# Author: Mathias Scharman, Franz Krah
# Date: 2025-07-14

# STEP 1: Generate initial VCF from BAMs, including invariant sites
# -------------------------------------------------------------------
bcftools mpileup -Ou \
  -f Antrodiella_citrinella_reassembly_enz_corr.fasta \
  --bam-list Ac_all.bam.list \
  --ignore-RG \
  --min-MQ 0 \
  --min-BQ 1 \
  --annotate FORMAT/AD,FORMAT/DP,INFO/AD | \
  bcftools call -m --skip-variants indels -Ov | \
  bgzip -c > Ac.vcf.gz

# STEP 2: Apply quality filters and allele balance filter
# ----------------------------------------------------------
vcftools --gzvcf Ac.vcf.gz \
  --mac 1 --minQ 20 --minDP 3 --max-meanDP 250 --recode --stdout | \
  bcftools filter - -S . -e '(FMT/AD[:0]+FMT/AD[:1]) < 3' | \
  python3 vcf_filter_allele_balance.py 0.25 0.75 \
  > tmp1

# STEP 3: Hardy-Weinberg Equilibrium filter
# ------------------------------------------
vcftools --vcf tmp1 --hardy

# Extract sites failing HWE (e.g. monomorphic sites)
tail -n +2 out.hwe | \
  sed 's/\//\t/g' | \
  awk '{ if($4==$3+$4+$5) print $1"\t"$2}' \
  > bad_sites.txt

# Filter out bad HWE sites and apply missingness threshold
vcftools --vcf tmp1 \
  --exclude-positions bad_sites.txt \
  --max-missing-count 108 --recode --stdout | \
  bgzip -c > tmp.3
tabix tmp.3

# STEP 4: Extract invariant sites for neutral models
# ---------------------------------------------------
vcftools --gzvcf Ac.vcf.gz \
  --max-missing-count 108 --max-maf 0 \
  --minDP 3 --max-meanDP 250 --recode --stdout | \
  bgzip -c > tmp.2
tabix tmp.2

# STEP 5: Combine SNPs and invariant sites
# -----------------------------------------
bcftools concat --allow-overlaps \
  tmp.3 \
```

```
  tmp.2 | \
  bcftools view --exclude-uncalled --trim-alt-alleles | \
  bgzip -c > Ac_filtered.vcf.gz

# STEP 6: Individual high coverage filtering (filter out repetitive
genes)
# ---------------------------------------------------------------
cd vcf
vcftools --gzvcf Ac_filtered.vcf.gz --geno-depth > Ac.gdepth.txt
mv out.gdepth Ac.gdepth.txt

pigz -p 5 -d -c Ac_filtered.vcf.gz | \
  python vcf_drop_genotypes_exceeding_3std_indiv_mean_depth.v3.py \
  --gdepth Ac.gdepth.txt | \
  bcftools view --threads 4 - \
  -i "COUNT(GT='mis')<=108" \
  --exclude-uncalled --trim-alt-alleles | \
  bgzip -c > Ac_filtered_individual.vcf.gz

# STEP 7: Final missing data and thinning filters
# ------------------------------------------------
vcftools --gzvcf Ac_filtered_individual.vcf.gz \
  --max-missing 0.9 --recode --stdout | \
  bgzip -c > Ac_filtered_individual_MM90percent.vcf.gz
tabix Ac_filtered_individual_MM90percent.vcf.gz


# STEP 8: Output SNPs only for downstream applications
# -----------------------------------------------------
vcftools --gzvcf Ac_filtered_individual_MM90percent.vcf.gz \
  --mac 1 --recode --stdout | \
  bgzip -c > Ac_filtered_individual_MM90percent_SNPs_only.vcf.gz
```
